# Exploring additive and non-additive genetic models to decipher the genetic regulation of almond tolerance to *Diaporthe amygdali*

**DOI:** 10.3389/fpls.2025.1608958

**Published:** 2025-09-19

**Authors:** Chiara Catalano, Giorgio Gusella, Ilaria Inzirillo, Giuseppe Cannizzaro, Mario Di Guardo, Stefano La Malfa, Giancarlo Polizzi, Alessandra Gentile, Gaetano Distefano

**Affiliations:** Department of Agriculture, Food and Environment, University of Catania, Catania, Italy

**Keywords:** *Prunus dulcis*, fungal disease, canker, breeding, GWAS

## Abstract

Constriction canker (*Diaporthe amygdali*) is one of the main diseases affecting almond cultivation. To unravel the genetic basis of the tolerance to the disease, a germplasm collection of 123 almond accessions (111 selected in Sicily, Italy, complemented with widely cultivated Italian and International varieties), was employed for a Genome-Wide Association Study (GWAS). Accessions were phenotyped employing a detached-twig inoculation assay, here employed for the first time for a GWAS, ensuring high throughputness and reproducibility. The most susceptible and tolerant accessions were also inoculated *in planta* and the two phenotyping methods showed a significant correlation of 0.7. Genotyping was performed using the Axiom™ 60K almond array, resulting in the identification of 47,496 robust markers. Both additive and non-additive GWAS models were tested leading to the identification of nine SNPs significantly associated with tolerance to *D. amygdali*. Candidate genes in linkage-disequilibrium with the significant SNPs were functionally characterized and a subset of 20 were further validated through RT-qPCR in both the most tolerant (the Sicilian ‘Cuti’) and susceptible (‘Ferraduel’) genotypes at 0 and at 2 days after *in planta* inoculations. The results provide novel insights to understand the genetic regulation of the tolerance to *D. amygdali* and for the set-up of marker-assisted selection plans in almond.

## 1 Introduction

Almond [*Prunus dulcis* (Mill.) D.A. Webb; syn. *P. amygdalus* Batsch] belongs to the genus *Prunus*, family *Rosaceae*, and is one of the most important nut trees worldwide. The production is mainly located in the USA (1,858,010 tons), Australia (360,328 tons), and Spain (245,990 tons). Italy is the second almond producer in Europe with 74,590 tons (FAOSTAT, 2023). The long history of cultivation of almond, is also witnessed by the selection, over the centuries, of more than 700 varieties differing for traits of agronomic interest (e.g.: productivity, nut quality). Despite this high genetic variability, the majority of the cultivars employed in productive orchards, are derived from a few elite cultivars that were intensively employed in breeding plans worldwide (Pérez de los Cobos et al., 2021). In Sicily, almond cultivation can be dated back to 2,000 BC, this long history of cultivation, coupled with the propagation by seeds, paved the way for the selection of varieties showing high tolerance to abiotic and biotic stresses and/or fruit quality traits of interest (Willcox et al., 2008; Distefano et al., 2013; Currò et al., 2015; Di Guardo et al., 2021).

In recent years, a significant impulse toward the elucidation of the structural genomics and genetic determinism of traits of agronomic interest was given by the release of the reference genome of three almond cultivars (Sánchez-Pérez et al., 2019; Alioto et al., 2020; D’Amico-Willman et al., 2022; Castanera et al., 2024) together with the development of a 60K almond SNP-chip array (Duval et al., 2023). The availability of these genomic tools greatly helped the set-up of marker-trait association studies aimed at the identification of molecular markers that can be readily employed for marker-assisted selection (MAS) and to unravel the genetic regulation of traits of agronomic interest. To date, MAS can rely on the availability of several molecular markers predictive for traits of agronomic interest such as: self-incompatibility (Tamura et al., 2000; Ballester et al., 2001; Ortega et al., 2005; Fernández i Martí et al., 2011; Gómez et al., 2019), shell hardiness (Arús et al., 1998; Sideli et al., 2023), flowering time (Ballester et al., 2001), and kernel taste (Sánchez-Pérez et al., 2010). On the contrary, little is known on the genetic mechanisms involved in the resistance toward biotic and abiotic stress, and molecular markers are publically available only for root-knot nematode resistance (Dirlewanger et al., 2004; Duval et al., 2014).

Within biotic stress, and fungal diseases in particular, high attention is given to the canker pathogens, with *Diaporthe amygdali* being the prevalent one (Diogo et al., 2010; León et al., 2020; Gusella et al., 2023). The symptoms consist of brown/silver cankers centered around the shoot nodes, quick desiccation of buds, flowers and leaves, and gummosis in proximity to the cankers. This characteristic symptom also gave the name to the disease “constriction canker” (Gusella et al., 2023). Despite the high aggressiveness of *D. amygdali*, the genetic basis of the host resistance is not yet fully elucidated and few molecular tools are available for the selection of tolerant varieties through marker-assisted selection. In this context, previous works focused on (i) characterizing the susceptibility to the disease in almond cultivars (Beluzan et al., 2022) and (ii) in the identification of molecular markers to be implemented for varietal screening in breeding programs (Martins et al., 2002, 2005). In Martins et al. (2002), four Random Amplified Polymorphic DNA markers (RAPD) showing polymorphic bands between tolerant and sensitive genotypes were developed.

In the present research, a germplasm collection of 123 almond varieties was investigated in a genome-wide association study aimed at identifying QTLs (Quantitative Trait Loci) associated with tolerance/susceptibility towards *D. amygdali*. To achieve this goal a reliable and scalable phenotyping protocol for assessing susceptibility to *D. amygdali* was developed. A wide almond germplasm collection was phenotyped and in parallel all the accessions were genotyped employing the first SNP-chip *ad hoc* developed for almond. Phenotypic and genotypic data were implemented in a GWAS analysis postulating additive and non-additive segregation models. The QTL intervals were further *in silico* annotated and candidate genes were validated through RT-qPCR. This study represents the first step to identify almond varieties as novel sources of tolerance to *D. amygdali* to be implemented in breeding programs and to investigate the genetic determinism regulating tolerance/susceptibility to canker.

## 2 Materials and methods

### 2.1 Plant material

Tolerance towards *D. amygdali* infection was determined in 123 individuals from the *ex situ* almond germplasm collection held at the Experimental Farm of the University of Catania (Sicily, Italy, latitude: 37°24′33″N, longitude: 15°03′20″E, altitude: 10 m asl; **Supplementary Table 1**). The germplasm collection is mainly composed of Sicilian accessions complemented with some of the most widely cultivated national and international cultivars as reference (Omodei, 2007). All genotypes are of the same age (three years) and are maintained in triplicates. The accessions were grafted onto the peach x almond rootstock GF-677 and subjected to standard agronomic practices.

### 2.2 Detached-twig assay for susceptibility towards *Diaporthe amygdali*

**Figure 1:**
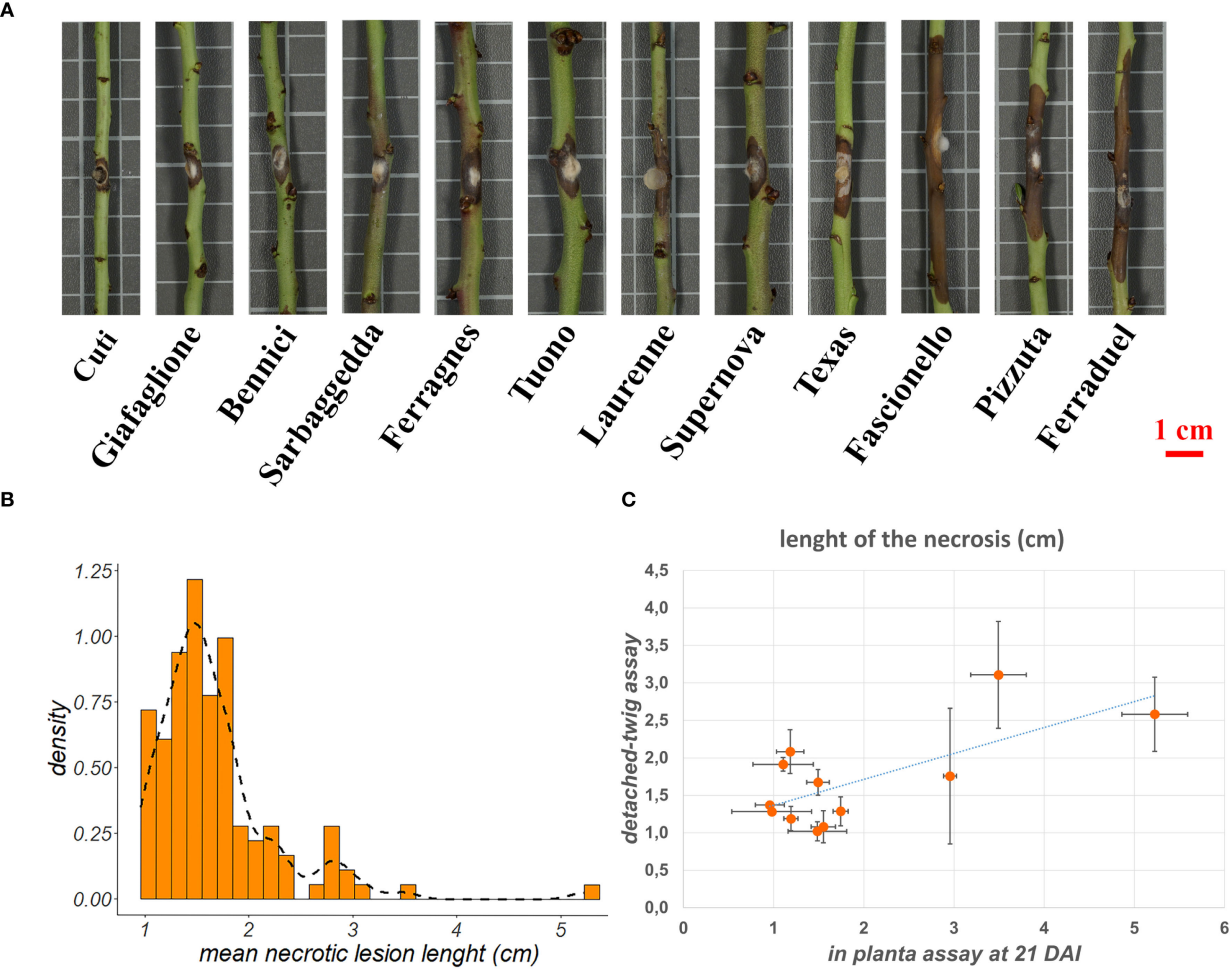
**(A)** Necrotic lesion length in a subset of twelve genotypes characterized by opposite behavior towards *Diaporthe amygdali* at 5 days after inoculation. **(B)** Distribution of the mean necrotic lesion length on the 123 almond accessions considered in the present study. **(C)** Scatter plot showing the correlation between the results obtained through detached-twig and *in planta* phenotyping for tolerance to *D*. *amygdali* on the twelve genotypes shown in section A of the figure.

To assess the differential response to *D. amygdali* infection, four growing twigs (~ 20 cm), lignified but still green (**Figure 1A**), were sampled from the selected genotypes. Twigs sterilization was conducted by dipping in a solution of ethanol (70%) for 30 s, followed by 1.5% sodium hypochlorite for 1 min, and finally in ethanol (70%) for 30 s. Once completely dried, twig’s ends were sealed by dipping into pruning wax to prevent desiccation and left to air dry overnight on a laboratory bench. Wounds were made by stinging the center of each twig with a sterile needle, instead of a cork borer which is much more invasive, to better simulate natural wounds. Mycelium agar plugs (5 mm in diameter obtained using a cork borer) were taken from an active 7-day-old colony of *D. amygdali* isolate VF1 growing on Potato dextrose agar (PDA, Lickson, Vicari, Italy). This strain was already genetically characterized and tested for pathogenicity (Gusella et al., 2023). Mycelial plugs were placed onto the twigs to favor the contact between the wounded portion of the plant and the mycelium (**Figure 1A**). A total of four twigs were tested for each almond cultivar and were kept into humid chambers (plastic boxes of 20 × 15 × 8 cm) filled with approximately 20 grams of sterile perlite at the bottom and finally filled with 200 ml of sterile water to maintain an optimal humidity. Plastic boxes were then moved into a growth chamber with a 12 hr photoperiod at 25 °C ± 1 °C for five days. At the same time, twigs from some genotypes were randomly selected for use as controls. Sterilization and preparation occurred as previously described. The wounds were created using the same technique, after which agar plugs without mycelium (sterile PDA plugs) were placed on top. Five days after inoculation, pictures of the inoculated stems were taken and lesion length was assessed using the software ImageJ (Schneider et al., 2012), by measuring the length of the necrotic lesion. Mean necrosis length was then employed as phenotypic data for the GWAS analysis. Descriptive statistical analyses (mean, standard deviation, standard error), histograms and density plots were performed using the ‘stat’ and the ‘ggplot2’ packages of the R software, respectively (Wickham, 2011; R Core Team, 2014). After lesion measurements, shoots were randomly collected among different cultivars to conduct fungal re-isolation to satisfy Koch’s postulates. Small twigs tissue (0.5 × 0.5 cm^2^) was cut from the discolored part, then the surface was sterilized with 1.5% sodium hypochlorite solution for 1 minute, rinsed in sterile distilled water, dried, and placed on PDA amended with 100 mg L-1 of streptomycin sulfate (Sigma-Aldrich, St. Louis, MO, USA) to avoid bacterial growth. Petri plates were then placed into an incubator at 25 ± 1 °C for 3–5 days with no light until fungal colonies were large enough to be examined morphologically. Finally, Pearson correlation coefficients were calculated between detached-twig phenotyping data and scores for flowering and ripening to verify any correlation between susceptibility to *D. amygdali* and flowering/ripening period as already reported in Beluzan et al. (2022).

### 2.3 In planta assay

To assess the reliability of the detached-twig phenotyping method, a subset of twelve accessions showing opposite responses, were further screened following an *in planta* assay (**Supplementary Table 1**). ‘Cuti’, ‘Giafaglione’, ‘Bennici’ and ‘Sarbaggedda’ were chosen as tolerant genotypes, while ‘Ferragnes’, ‘Tuono’, ‘Laurenne’, ‘Supernova’, ‘Texas’, ‘Fascionello’, ‘Pizzuta’ and ‘Ferraduel’ were chosen as susceptible and very susceptible genotypes. The selected genotypes showing the highest and the lowest mean necrosis length were propagated in three biological replicates by grafting onto GF-677 and held in 20 cm^3^ pots. Additionally, alphanumeric codes were assigned to genotypes and biological replicates to not influence the operator. *In planta* assays were conducted by inoculating four twigs per plant for a total of 12 inoculation points per genotype, following the same methodology described above. For each genotype, a wound was created and a uncolonized sterile PDA plugs was placed as a negative control. Plants were placed in a growth chamber with a 12-hour photoperiod at 25 °C ± 1 °C. Inoculation points were monitored for 21 days after inoculation and necrosis length was assessed by using a digital caliper. Moreover, inoculated stem segments of one genotype showing the highest tolerance (‘Cuti’) or susceptibility (‘Ferraduel’) were collected at 0 and 2 days after inoculation (D0 and D2, respectively) and stored at -80 °C for transcriptome analysis. Pearson correlation coefficient was calculated between phenotyping data obtained in the detached-twig and *in planta* assays.

### 2.4 SNP-chip array genotyping and GWAS analysis

The 123 accessions were genotyped employing the Axiom™ 60K SNP Array (Duval et al., 2023). DNA extraction and quality check were performed as described in Gentile et al., 2024. The GWAS analysis was conducted by exploring both additive and non-additive genetic models (i.e.: dominant, recessive and overdominant). For each genetic model, the original genotypic data were transformed as described in Pérez de los Cobos et al. (2023). Briefly, given a biallelic SNP characterized by alleles *a* (reference allele) and *b* (alternative allele), the codominant model was characterized by two homozygous and one heterozygous genotype (*aa*; *ab*; *bb*). In the dominant model, the *aa* and *ab* genotypes were grouped together (*aa* + *ab*; *bb*), while in the recessive model the *ab* and *bb* were instead considered as a unique genotypic class (*aa*; *ab* + *bb*). As for the overdominance genetic model, the two homozygous genotypes were grouped together and compared against the heterozygous genotype (*aa* + *bb*; *ab*).

The GWAS analysis was performed employing the Bayesian information and Linkage-disequilibrium Iteratively Nested Keyway (BLINK) model (Huang et al., 2019) implemented in the R package GAPIT (Lipka et al., 2012).

The BLINK model is based on two fixed effect models and one filtering process which identify significant SNPs that are not in LD with each other as covariates. The first model can be written as:


(1) $$y_{i}=S_{i1}^{\cdot }b_{1}+S_{i2}^{\cdot }b_{2}+\ldots +S_{ik}^{\cdot }b_{k}+S_{ij}d_{j}+e_{1}$$

In Equation 1 the *P* value of all tested markers is calculated. In particular, y_i_ refers to the phenotype of the i^th^ accession, S_i1:k_ are the genotypes of the k SNP(s) passing the significance threshold, while their corresponding effects are reported on the b_1:k_ term. S_ij_ and d_j_ are the genotype of the i^th^ accession and the corresponding effect respectively, while the residual variance is expressed as e_i_.

Then, the set of SNPs exceeding the significance threshold is further evaluated on the following equation:


(2) $$y_{i}=S_{i1}^{\cdot }+S_{i2}^{\cdot }+\ldots +S_{ik}^{\cdot }+e_{i}$$

In (2) only the SNPs exceeding the significance threshold are evaluated, the final number of covariates is selected according to the BIC method.


(3) BIC=−2LL+2kLn(n)

In which -2LL is twice the negative log of the likelihood and k is the number of SNPs exceeding the significance threshold, Ln is the natural logarithm and n is the total number of individuals in the analysis.

The total set of markers employed for the GWAS are evaluated in (1) and for each SNP the corresponding *P* value is calculated. Those SNPs exceeding the significance threshold (adjusted for multiple testing using Bonferroni with a α  = 0.01) are sorted according to their *P* value. Then, if the SNP with the second lowest *P* value shows a Pearson correlation higher than 0.7 with the SNP with the lowest *P* value, it is also discarded; and the process is repeated iteratively for all SNPs exceeding the significance threshold. In Equation 2, an increasing number of significant SNPs is included in the model and the corresponding BIC (3) is calculated. Then, the set of significant SNPs that give the best BIC is employed again as covariates in (1) and the process is repeated iteratively until the set of significant SNPs selected remains the same.

Unlike other GWAS models based on the definition of genetic windows (bin), BLINK works directly on markers and does not rely on the assumption that candidate genes are uniformly distributed across the genome. The model is based on the assumption that, in case of marker-trait association(s), the marker showing the highest significance is taken as reference while all the others exceeding the significance threshold (and in linkage disequilibrium with the reference SNP), are discarded. This process is repeated iteratively until no linkage disequilibrium occurs between the markers being significantly associated with the trait. Furthermore, the BLINK model implemented the Bayesian Information Content (BIC) in a fixed-effect model for the detection of significant marker-trait associations overcoming the limitation in computing time represented by the maximum likelihood method often employed as random effect in other GWAS models. The first 3 principal components were included in the model to take genetic stratification into adequate account. The phenotypic distribution given each significant SNP detected in the GWAS analysis was visually inspected to confirm the agreement between the phenotypic distribution and the specific genetic model tested (Pérez de los Cobos et al., 2023).

### 2.5 Annotation of the QTL interval

A genomic window of 60 Kb upstream and downstream the significant SNPs was annotated *in silico* to detect candidate genes putatively associated to tolerance/susceptibility to *D. amygdali*. The genomic interval for gene annotation was based on previous evaluation of the LD decay in almond (Di Guardo et al., 2021). The gene annotation was carried out employing the *Prunus dulcis* reference genome, cv. Texas v2.0 (Alioto et al., 2020), candidate genes were functionally annotated employing InterProScan (considering both IPR description and the gene ontology) and the Kyoto Encyclopedia of Genes and Genomes (KEGG) evaluating both orthologs and pathways (https://www.rosaceae.org/analysis/295). Furthermore, the predicted effect of each significant SNP on gene expression was assessed employing SnpEff software (Cingolani et al., 2012).

### 2.6 Expression analysis via RT-qPCR

Twenty genes were further selected for gene expression analysis according to their functional annotation (including ontology terms related to the defense response in plants) and literature reporting on their role in defense response even in other pathosystems, then cited in the Discussion section. Stems collected from ‘Cuti’ and ‘Ferraduel’ genotypes at 0 and 2 days after inoculation during the *in planta* assay and stored at -80 °C were homogenized in liquid nitrogen, and 100 mg were used for RNA extraction. Total RNA was extracted according to the protocol described in Catalano et al., 2020. One volume of extraction buffer (0.2 M TRIS pH 8.0, 0.2 M NaCl, 50 mM EDTA, 2% (w/v) SDS), one volume of phenol, and 0.02 volume of β-mercaptoethanol were added to the sample. After incubation at 50 °C for 5 min, samples were centrifuged at 4000 rpm at 4 °C for 15 min. Two cycles of centrifugation were carried out, adding to the upper aqueous phase one volume of chloroform:isoamyl alcohol (24:1, v/v). RNA was precipitated with one-half volume of 6 M LiCl to the upper phase at −20 °C overnight. After centrifugation at 8500 rpm for 40 min, the precipitated RNA was washed with 70% (v/v) ethanol and centrifuged at 7500 rpm for 20 min. The total RNA was eluted in 50 μL of RNase-free water. RNA quality and quantity were evaluated using a Nanodrop 2000c spectrophotometer (Thermo Fischer Scientific) and by gel electrophoresis (agarose 1.0% in TAE 1x). Quality was considered optimal with 260/280 ratio values between 1.8 and 2.0. cDNA synthesis was performed by using the High-Capacity cDNA reverse Transcription Kit (Thermo Fisher Scientific) following manufacturer’s instructions. Thermal cycler conditions for cDNA synthesis were: 10 min at 25 °C, 37 °C for 120 min, and 85 °C for 5 s. RT-PCR was carried out using the Rotor-Gene Q thermal cycler (Qiagen). The PCR mixture contained 50 mM MgCl2, 1x NH4, 5 μM dNTPs, 50 μM SYTO-9, 0,2 units of Taq polymerase, 10 μM of each gene-specific forward and reverse primer, and 100 ng of the cDNA sample, in a final volume of 20 μl. The standard thermal profile was used for all PCRs and consisted of 95 °C for 5 minutes, followed by 40 cycles at 95 °C for 5 seconds, 59 °C for 20 seconds, 72 °C for 2 minutes, 95 °C for 1 minute, 40 °C for 1 minute, melting from 60 °C to 92 °C holding 2 seconds between each 0.2 °C temperature step. The melting curve was useful for excluding the formation of nonspecific amplicons and dimers since only one pick was observed for each gene. Two technical replicates were assayed for each biological replicate, and a no-template negative control was routinely included in each reaction. Primers were designed using Primer 3 software with the default settings (Koressaar et al., 2018) and listed in **Supplementary Table 2**. Actin was selected as a housekeeping gene to normalize gene expression data (forward: 5’CTGGACTCTGGTGATGGTGT3’, reverse: 5’AGCAAGGTCCAGACGAAGAA3’). In preliminary assay aimed at defining the best housekeeping gene for gene expression analysis, also *Tubulin alpha 3-chain* and *Elongation factor 2-like* were tested with cDNA mix at different concentrations, but results were not satisfactory, so we proceeded by using one single housekeeping gene (Lin et al., 2018). Data analysis was carried out using the normalized 2−ΔΔCT method, and qRT-PCR results between genotype groups were compared according to the normalized Ct value for each gene. Results are also discussed in terms of ‘fold change’ with respect to the calibrator, represented by the susceptible genotype at T0. Standard deviation, standard error and Student’s T test were calculated by using Excel Software (Microsoft Corporation).

## 3 Results

### 3.1 Evaluation of almond susceptibility towards *Diaporthe amygdali*

The susceptibility/tolerance toward infection of *D. amygdali* was assessed through the measurement of the necrosis length in a detached-twig assay on 123 almond accessions at five days after inoculation. Four replicates were tested per each genotype, for a total of 492 assayed twigs. Overall, the length of the necrosis showed a wide variability across the germplasm (**Supplementary Table 1**; **Figures 1A, B**). The mean necrotic lesion length ranged from values lower than 1 cm (0.96 cm for ‘Cuti’ and ‘Filippazzo’, 0.98 cm for ‘Giafaglione’ and ‘Uova di Cucco’ and 0.99 for ‘Sarbaggia Di Vitello’) to 5.23 cm for ‘Ferraduel’, followed by ‘Pizzuta (Bronte)’ (3.49 cm) and ‘Sancisuca’ (3.16 cm).

The 81% of the genotypes were characterised by an average length of the necrotic lesion lower than 2 cm (**Figure 1C**). Samples were clustered in three groups according to the mean lesion length as follow: low-susceptible genotypes (necrotic lesion length lower than 1 cm), susceptible genotypes (lesion length ranking from 1 cm to 2 cm), and very susceptible genotypes (necrotic lesion higher than 2 cm). Finally, no statistically significant correlation was found between susceptibility to *D. amygdali* and flowering or ripening periods (data not shown). To further validate the proposed detached-twig assay, twelve genotypes showing opposite behavior toward *D. amygdali* (**Supplementary Table 1**; **Figure 1A**), were selected and employed for an *in planta* assay. The two phenotypic methods showed a positive correlation (0.7, p value < 0.05; **Figure 1C**), the highest.

### 3.2 GWAS analysis and *in silico* annotation of the candidate genes

**Figure 2:**
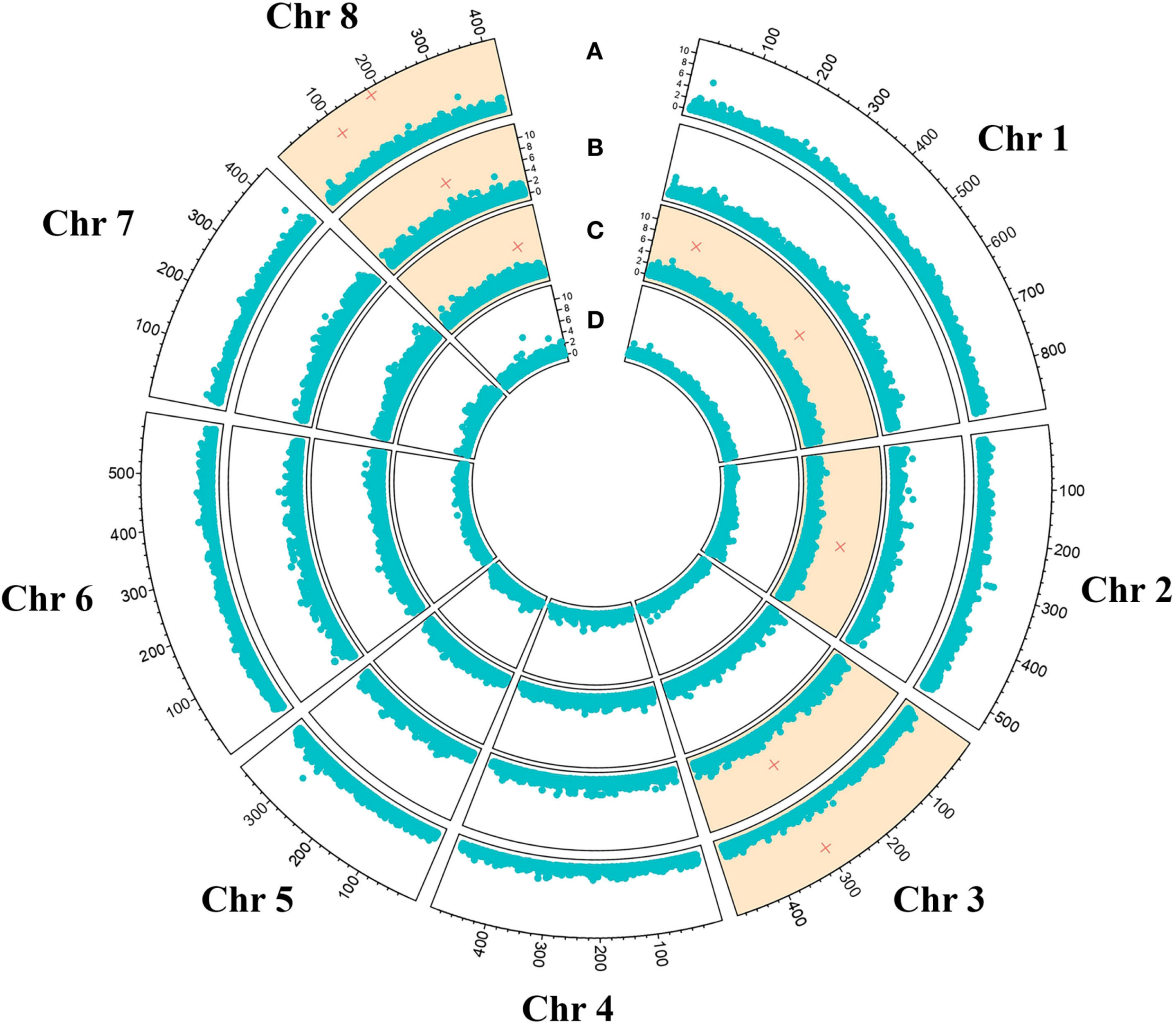
GWAS results employing overdominance **(A)**, dominance **(B)**, recessive **(C)**, and codominant **(D)** models. Significant SNPs were depicted as red cross and chromosomes showing at least one significant marker-trait association were evidenced in orange.

Phenotypic data resulting from the detached-twig assay were integrated with the genetic data in a GWAS analysis. The four genetic models tested (i.e. codominance, dominance, recessive, overdominance) led to the identification of nine SNPs exceeding the significance threshold located in four chromosomes as shown in **Figure 2**; **Table 1**. In particular, the overdominance model led to the identification of 3 significant SNPs located in chromosome three (1) and chromosome eight (2), while a total of 4 SNPs were detected postulating a recessive genetic model: two in chromosome one, and respectively in chromosomes two and eight. The exploration of the dominance model led to the identification of 1 significant SNP in chromosome three and chromosome eight, while no significant marker-trait association was detected employing the codominant genetic model (**Figure 2**; **Table 1**). All three genetic models showed at least one significant association in chromosome 8 even though the SNPs were located in a relatively wide genomic region spanning from 5,124,915 bp (AX-586143715, overdominance model) to 17,281,509 (AX-586150343, recessive model). Conversely, in both Dominance and Overdominance models, the significant SNPs detected in chromosome 3 were presumably in the same linkage block being only 43.3 Kb apart. The percentage of phenotypic variance explained ranged from 3% to 67.99% for markers AX-586048050 (recessive) and AX-586146007 (overdominance) respectively.

**Table 1:** SNPs identified through the GWAS analysis performed in the present study.

Genetic model	SNP	Chr	Pos	-log10 (p value)	Phenotypic variance explained [%]
Overdominance	AX-586070824	3	15098692	8.26	8.29
	AX-586143715	8	5124915	8.39	3.24
	AX-586146007	8	9169041	11.23	67.99
Recessive	AX-586016477	1	5934933	7.39	49.27
	AX-586027373	1	26769078	6.45	13.56
	AX-586048050	2	14420961	6.69	3
	AX-586150343	8	17281509	6.06	11.56
Dominance	AX-586069960	3	15055364	6.42	14.56
	AX-586144793	8	11559168	6.70	25.16

**Figure 3:**
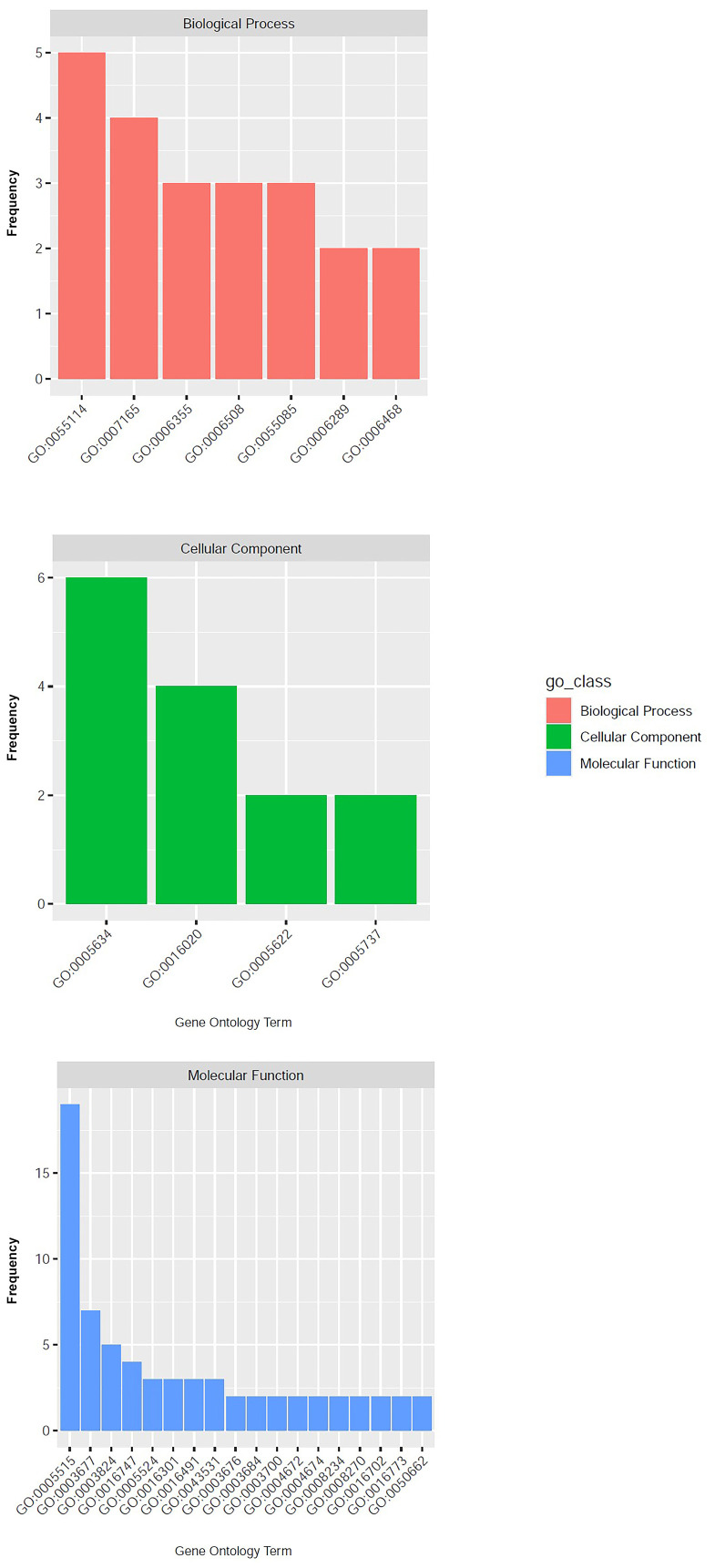
Results of gene ontology analysis with the GO terms detected at least twice grouped according to the corresponding classes: ‘Biological process’, ‘Cellular Component’ and ‘Molecular Function’.

A genomic region spanning 60,000 bp upstream and downstream the nine significant SNPs was annotated *in silico* enabling the detection of candidate genes putatively involved in the control of the tolerance/susceptibility to *D*. *amygdali*. The analysis led to the identification of 133 genes as reported in **Supplementary Table 3**. This set of genes corresponded to 98 univocal GO terms, most of those (47) were associated with the class of Molecular Function, followed by Biological Process (37) and Cellular Component (17; **Figure 3**). The most represented GO term was ‘protein binding’ (GO:0005515, Molecular Function), associated to 22 genes; followed by ‘nucleus’ (GO:0005634, Cellular Component) and ‘DNA binding’ (GO:0003677, Molecular Function) associated to 11 and 9 genes respectively (**Figure 3**; **Supplementary Table 3**). The effect of the nine SNPs detected on the gene expression was further assessed employing the SNPeff software. SNP AX-586150343 resulted in the occurrence of a splicing region in the gene Prudul26A001570T1, while 3 SNPs were associated to intron variants in genes Prudul26A032316T1 (AX-586069960 and AX-586070824) and Prudul26A016539T1 (AX-586146007; **Supplementary Table 3**). Among the set of candidate genes, 8 and 5 were characterised instead by the occurrence of variants upstream and downstream respectively caused, in both cases, by 4 SNPs (**Supplementary Table 3**).

### 3.3 Expression analysis via RT-qPCR

**Figure 4:**
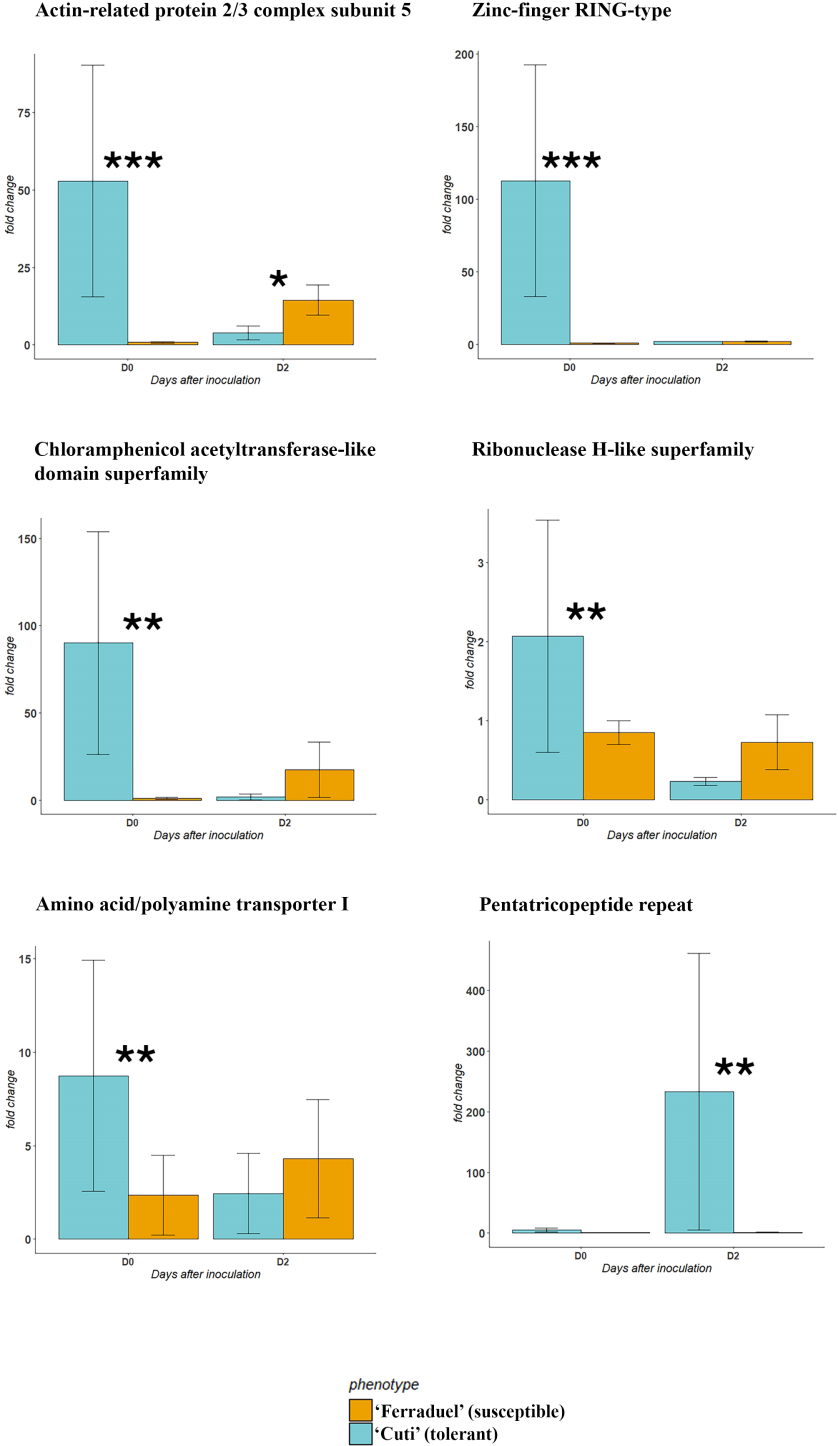
Results of the RT-qPCR analysis performed for the candidate genes identified in silico at 0 (D0) and 2 (D2) days after inoculation. The accessions tested were ‘Cuti’ (tolerant, in blue) and ‘Ferraduel’ (susceptible, in orange). Among the twenty candidate genes tested, only those showing significant differences in the expression (* for p value < 0.05, ** for p value < 0.01, *** for p value < 0.001 in the Student’s T test) were presented.

A subset of 20 genes were selected based on functional annotation and literature evidence and further validated through RT-qPCR (**Supplementary Table 2**, **Supplementary Figure 1**). The transcriptomic analysis was carried out on the accessions showing the highest (‘Cuti’) and lowest (‘Ferraduel’) phenotypic scores of the *in planta* assay. Among the 20 candidate genes tested, 5 were significantly overexpressed in the tolerant accession at D0, namely: *actin-related protein 2/3 complex subunit 5*, *amino acid/polyamine transporter I*, *chloramphenicol acetyltransferase-like domain superfamily*, *zinc finger RING-type* and *ribonuclease H-like superfamily* (**Figure 4**). Conversely, the *pentatricopeptide repeat* gene was significantly overexpressed in ‘Cuti’ at D2 (**Figure 4**). While none of the tested genes was overexpressed in ‘Ferraduel’ compared to ‘Cuti’ for both time points.

## 4 Discussion

### 4.1 Highlights of almond breeding for tolerance to *Diaporthe amygdali*

Canker pathogens represent one of the major threats affecting almond cultivation worldwide. Investigations conducted in the Mediterranean basin revealed the relevant role of the genus *Diaporthe* (previously reported as *Phomopsis*), with *D. amygdali* being the most prevalent (Diogo et al., 2010; León et al., 2020; Gusella et al., 2023). In the last decades, the wide employment of ‘Tuono’, ‘Cristomorto’ and ‘Nonpareil’ cultivars in most of the breeding programs worldwide led to a significant reduction in the genetic variability of the novel varieties (Pérez de los Cobos et al., 2021). Nevertheless, the characterization of local accessions can allow the selection of genetic variability sources that could be implemented in new breeding programs. In this context, several germplasm collections were screened for the tolerance toward constriction canker in Spain (Beluzan et al., 2022) and Hungary (Varjas et al., 2017) leading to the identification of a broad range of tolerant genotypes. Similarly, Cabrita et al. (2004), described the higher tolerance toward *D. amygdali* of the local cultivar ‘Barrinho Grado’ compared to ‘Ferragnes’.

### 4.2 Set up of a large-scale phenotyping screening assay to assess the susceptibility toward *Diaporthe amygdali*

Despite the high economic losses due to constriction canker, to date its genetic determinism in almond is not yet fully elucidated. This is partially due to the constraints represented by the set-up of large-scale phenotyping assay and the availability of large germplasm collections, both fundamental prerequisites to perform a robust marker-trait association study. To this extent, a novel method based on a detached-twig assay was adopted in light of its suitability for large-scale screening. *In planta* assays (such as mycelial plug) are widely considered a gold standard in susceptibility trials but showed serious limitations in terms of throughputness when large germplasms are screened. On the other side, the detached twig (stem or leaf) assay is commonly adopted in plant pathology for the assessment of preliminary results of pathogenicity tests (León et al., 2020), to assess cultivar susceptibility (Beluzan et al., 2022), and the effects of environmental factors on disease development (Luo et al., 2022) but were never employed for the set-up of a marker-trait association analysis.

Although the detached method can be considered a valuable resource for large-scale analyses, especially in terms of reproducibility and accuracy of symptoms assessment, some differences were observed with the *in planta* inoculation. These discrepancies reflect the fact that the detached tissues do not entirely reflect the behavior of the whole plant in terms of defense response in contrasting the disease development.

In our phenotyping assay, the mycelial plug technique was used for testing a high number of genotypes and the woundings were performed with an insulin needle, instead of cork borer which is much more invasive, to better simulate natural wounds. In this work, the detached-twig assay for *D. amygdali* inoculation was successfully developed and showed a positive correlation with the *in planta* assay, confirming its suitability for large-scale screening assays (**Figure 1**). In fact, the correlation coefficient obtained (0.7) can be considered high in line with results obtained by other authors in similar pathosystems (Hulin et al., 2018; Mancero-Castillo et al., 2024).

Nevertheless, some differences in the two phenotyping methods were detected: this can be probably reconducted to the fact that, in the detached method, the host defense mechanism is not fully activated due to the limited portion of plant examined. In addition, *D. amygdali* (similarly to what observed with many other canker-causing pathogens) causes a complex symptomatology spanning from necrotic lesion of the woody tissues (the trait evaluated employing the detached method), to fruit blight, defoliation, dieback, flower blight and others that were not evaluated with the proposed method and can contribute in explaining the differences in the two phenotyping methods.

The germplasm collection showed a high variability in the response to *D. amygdali*, in particular, five almond cultivars (‘Cuti’, ‘Filippazzo’, ‘Giafaglione’, ‘Uova di Cucco’ and ‘Sarbaggia di Vitello’) showed tolerance to the disease with an average necrosis length lower than 1 cm. For those varieties also assayed in previous research, we confirmed the high susceptibility of ‘Ferraduel’ (5.23 cm lesion length), for this reason the cultivar is often employed as reference in pathogenicity test (Goura et al., 2022; Ören and Bayraktar, 2025), and the susceptibility of ‘Ferragnès’ (1.19 cm), ‘Tuono’ (1.49 cm) and ‘Texas’ (1.74 cm) (Diogo et al., 2010; Ören and Bayraktar, 2025). Despite previous works proposed a correlation between the blooming and ripening time and susceptibility (Beluzan et al., 2022), these traits were not significantly correlated in our dataset (data not shown).

### 4.3 Development of a GWAS analysis and *in silico* identification of candidate genes

The GWAS analysis led to the identification of a total of nine SNPs significantly associated with tolerance/susceptibility toward *D. amygdali* (**Figure 2**). To better outline the genetic determinism of the trait, four models were tested postulating additive and non-additive (i.e. dominance, recessive, overdominance) marker-trait association models leading to the identification of 4 SNPs for the recessive methods and 3 and 2 for the overdominant and dominant models respectively, while no significant marker-trait association was detected for the additive model (**Table 1**). The two SNPs located on chromosome 1 (AX-586016477 and AX-586027373, **Table 1**) were characterized by the B allele conferring tolerance either in single or double dosage; the B allele was instead associated to higher susceptibility (in single or double dosage) for AX-586048050 (chromosome 2) and AX-586144793 (chromosome 8, **Table 1**). The detection of markers showing non-additive genetic models is of particular interest for the development of edited plants, especially for the knock-out of susceptibility allele(s). As for the overdominance model, SNPs AX-586070824 and AX-586143715 were both characterized by a higher resistance for the heterozygous genotype compared to the two homozygous conditions, For the remaining three SNPs one of the genetic classes was absent hampering a conclusive resolution of the role of allele dosage on the phenotype.

Four out of nine detected SNPs were identified employing the recessive genetic model suggesting the occurrence of recessive alleles controlling the resistance to the disease as already shown for anthracnose in lentil (Buchwaldt et al., 2013). On the other side, in both strawberry (Rehman et al., 2025) and wheat (Han et al., 2023), marker-trait association analysis detected resistance genes showing a dominant and/or overdominant genetic model on the germplasm in analysis. This result was also in line with what was described by Pérez de los Cobos et al. (2023) and Roth et al. (2024) highlighting the significant predominance of QTLs detected with non-additive models compared to the most widely employed additive model and enforcing the need for the exploration of alternative genetic models in marker-trait association analysis. In addition, the detection of a few significant SNPs highlighted the fast linkage-disequilibrium decay of almond, as already described in Di Guardo et al. (2023) and Pérez de los Cobos et al. (2023).

### 4.4 Validation of the candidate genes through RT-PCR

According to gene ontology categories and literature evidence, twenty candidate genes were further validated through a gene expression analysis (**Supplementary Figure 1**). In particular, five genes were highly expressed in the tolerant genotype (‘Cuti’) compared to the susceptible (‘Ferraduel’) at D0 (**Figure 4**). Among these, two genes (the *actin-related protein 2/3 complex* and the *chloramphenicol acetyltransferase-like domain superfamily)* are actively involved in the response to wounding (Ryan and An, 1988; Opalski et al., 2005). The activation of these genes in ‘Cuti’ suggests a potential role of wounding in triggering the defense response in the plant (Lee and Seo, 2022). In addition, other three genes involved in the plant defense response were also overexpressed in the tolerant genotype: an *amino acid/polyamine transporters*, involved in amino acid transport, such as tryptophan and methionine, which are precursors of secondary metabolites with antimicrobial effects (Ahuja et al., 2012); a RING zinc-finger protein that is activated in response to abiotic stress and in plant immunity, and a *ribonuclease H-like superfamily* gene that is involved in plant defense responses in *Arabidopsis thaliana* (Walley et al., 2010). Moreover, at D2, the *pentatricopeptide repeat* gene, which was demonstrated to be involved in response to biotic and abiotic stresses (Wang and Tan, 2024), was significantly overexpressed in the tolerant genotype than in the susceptible one (**Figure 4**). Pentatricopeptide repeat proteins belong to a large family of proteins characterized by a tandem degenerate repeat of almost 35 amino acid residues and have been studied for their role in post-transcriptional processing events, in particular the activation or repression of the translation of specific mRNA (Wang and Tan, 2024). In kiwifruit, pentatricopeptide repeat proteins were demonstrated to regulate the resistance adaptation to bacterial disease via the modulation of RNA editing (Zhang et al., 2023). Our results support the hypothesis that such a mechanism is also possible considering the almond-*D*. *amygdali* interaction.

## 5 Conclusion

Breeding programs for tree crops take advantage from the identification of molecular markers and candidate genes associated with traits of interest (productivity, fruit quality, resistance to biotic and abiotic stress) to be implemented in Marker-Assisted Selection (MAS) and in the application of New Genomic Techniques (NGTs). To our knowledge, this is the first study employing a GWAS analysis aimed at identifying SNPs markers and candidate genes associated with tolerance/susceptibility to *D. amygdali*, an aggressive pathogen causing canker disease. The presented results represent the first step toward the definition of molecular markers that can be readily employed for marker-assisted selection, an important possibility for long-term disease management, especially in the case of canker diseases considered difficult to control and provide novel insights on the genetic mechanisms regulating the tolerance/susceptibility towards *D. amygdali*.

## Data Availability

The original contributions presented in the study are included in the article/**Supplementary Material**, further inquiries can be directed to the corresponding author/s.

## Supplementary material

Supplementary Table 1 Almond varieties used in this study with results related to the detached-twig assay. Asterisks indicate those varieties used also in the *in planta* assay.

Supplementary Table 2 Primer designed for the RT-qPCR performed in this study.

Supplementary Table 3 List of genes resulted from the gene ontology analysis and annotation of those sequences putatively involved in the control of the tolerance/susceptibility to *Diaporthe amygdali.*

Supplementary Figure 1 Results of RT-qPCR analysis conducted on the twenty candidate genes identified through the GWAS analysis.

## References

[B1] Ahuja I. Kissen R. Bones A. M. (2012). Phytoalexins in defense against pathogens. Trends Plant Sci. 17, 73–90. doi: 10.1016/j.tplants.2011.11.002, PMID: 22209038

[B2] Alioto T. Alexiou K. G. Bardil A. Barteri F. Castanera R. Cruz F.. (2020). Transposons played a major role in the diversification between the closely related almond and peach genomes: results from the almond genome sequence. Plant J. 101, 455–472. doi: 10.1111/tpj.14538, PMID: 31529539 PMC7004133

[B3] Arús P. Ballester J. Jáuregui B. Joobeur T. Truco M. J. De Vicente M. C. (1998). The european prunus mapping project: Update on marker development in almond. Acta Hortic. 484, 331–338. doi: 10.17660/actahortic.1998.484.57

[B4] Ballester J. Socias I Company, R Arus P. De Vicente M. C. (2001). Genetic mapping of a major gene delaying blooming time in almond. Plant Breed. 120, 268–270. doi: 10.1046/j.1439-0523.2001.00604.x

[B5] Beluzan F. Miarnau X. Torguet L. Zazurca L. Abad-Campo P. Luque J.. (2022). Susceptibility of Almond (Prunus dulcis) Cultivars to Twig Canker and Shoot Blight Caused by Diaporthe amygdali. Plant Dis. 106, 1890–1897. doi: 10.1094/PDIS-09-21-1875-RE, PMID: 35021872

[B6] Buchwaldt L. Shaikh R. Adam J. Tullu A. Slinkard A. E. (2013). Recessive and dominant genes confer resistance to Colletotrichum truncatum in cultivated lentil. Can. J. Plant Pathol. 35, 222–231. doi: 10.1080/07060661.2013.768296

[B7] Cabrita L. Neves A. Leitão J. (2004). Evaluation of resistance to phomopsis amygdali in Almond. Acta Hortic. 663, 235–238. doi: 10.17660/ActaHortic.2004.663.37

[B8] Castanera R. de Tomás C. Ruggieri V. Vicient C. Eduardo I. Aranzana M. J.. (2024). A phased genome of the highly heterozygous ‘Texas’ almond uncovers patterns of allele-specific expression linked to heterozygous structural variants. Hortic. Res. 11. doi: 10.1093/hr/uhae106, PMID: 38883330 PMC11179849

[B9] Catalano C. Ciacciulli A. Salonia F. Russo M. P. Caruso P. Caruso M.. (2020). Target-genes reveal species and genotypic specificity of anthocyanin pigmentation in citrus and related genera. Genes (Basel). 11, 807. doi: 10.3390/genes11070807, PMID: 32708660 PMC7397085

[B10] Cingolani P. Platts A. Wang L. L. Coon M. Nguyen T. Wang L.. (2012). A program for annotating and predicting the effects of single nucleotide polymorphisms, SnpEff. Fly (Austin). 6, 80–92. doi: 10.4161/fly.19695, PMID: 22728672 PMC3679285

[B11] Currò S. La Malfa S. Distefano G. Long G. Sottile F. Gentile A. (2015). Analysis of S-allele genetic diversity in Sicilian almond germplasm comparing different molecular methods. Plant Breed. 134, 713–718. doi: 10.1111/pbr.12318

[B12] D’Amico-Willman K. M. Ouma W. Z. Meulia T. Sideli G. M. Gradziel T. M. Fresnedo-Ramírez J. (2022). Whole-genome sequence and methylome profiling of the almond [*Prunus dulcis* (Mill.) D.A. Webb] cultivar ‘Nonpareil.’. G3 Genes|Genomes|Genetics 12. doi: 10.1093/g3journal/jkac065, PMID: 35325123 PMC9073694

[B13] Di Guardo M. Farneti B. Khomenko I. Modica G. Mosca A. Distefano G.. (2021). Genetic characterization of an almond germplasm collection and volatilome profiling of raw and roasted kernels. Hortic. Res. 8, 27. doi: 10.1038/s41438-021-00465-7, PMID: 33518710 PMC7848010

[B14] Di Guardo M. Moretto M. Moser M. Catalano C. Troggio M. Deng Z.. (2023). De novo assembly of Citrus limon and target-sequence genotyping toward the detection of genes involved in tolerance to ‘mal secco’ disease. Acta Hortic, 231–238. doi: 10.17660/ActaHortic.2023.1362.31

[B15] Diogo E. L. F. Santos J. M. Phillips A. J. L. (2010). Phylogeny, morphology and pathogenicity of Diaporthe and Phomopsis species on almond in Portugal. Fungal Divers. 44, 107–115. doi: 10.1007/s13225-010-0057-x

[B16] Dirlewanger E. Cosson P. Howad W. Capdeville G. Bosselut N. Claverie M.. (2004). Microsatellite genetic linkage maps of myrobalan plum and an almond-peach hybrid?location of root-knot nematode resistance genes. Theor. Appl. Genet. 109, 827–838. doi: 10.1007/s00122-004-1694-9, PMID: 15241595

[B17] Distefano G. Caruso M. La Malfa S. Ferrante T. Del Signore B. Gentile A.. (2013). Genetic diversity and relationships among Italian and foreign almond germplasm as revealed by microsatellite markers. Sci. Hortic. (Amsterdam). 162, 305–312. doi: 10.1016/j.scienta.2013.08.030

[B18] Duval H. Coindre E. Ramos-Onsins S. E. Alexiou K. G. Rubio-Cabetas M. J. Martínez-García P. J.. (2023). Development and evaluation of an axiomTM 60K SNP array for almond (Prunus dulcis). Plants 12, 242. doi: 10.3390/plants12020242, PMID: 36678957 PMC9866729

[B19] Duval H. Hoerter M. Polidori J. Confolent C. Masse M. Moretti A.. (2014). High-resolution mapping of the RMia gene for resistance to root-knot nematodes in peach. *Tree Genet* . Genomes 10, 297–306. doi: 10.1007/s11295-013-0683-z

[B20] FAOSTAT (2023). No Title.

[B21] Fernández i Martí À. Howad W. Tao R. Segura J. M. A. Arús P. Socias i Company R. (2011). Identification of quantitative trait loci associated with self-compatibility in a Prunus species. Tree Genet. Genomes 7, 629–639. doi: 10.1007/s11295-010-0362-2

[B22] Gentile A. Inzirillo I. Bennici S. Scollo F. Las Casas G. Di Guardo M.. (2024). Genomic approaches for almond traceability from nursery and along the food chain. Hortic. Plant J. 11(3), 1103–1115. doi: 10.1016/j.hpj.2023.12.013

[B23] Gómez E. M. Dicenta F. Batlle I. Romero A. Ortega E. (2019). Cross-incompatibility in the cultivated almond (Prunus dulcis): Updating, revision and correction. Sci. Hortic. (Amsterdam). 245, 218–223. doi: 10.1016/j.scienta.2018.09.054

[B24] Goura K. Lahlali R. Bouchane O. Baala M. Radouane N. Kenfaoui J.. (2022). Identification and characterization of fungal pathogens causing trunk and branch cankers of almond trees in Morocco. Agronomy 13, 130. doi: 10.3390/agronomy13010130

[B25] Gusella G. La Quatra G. Agustí-Brisach C. Trapero A. Polizzi G. (2023). Elucidating the almond constriction canker caused by Diaporthe amygdali in Sicily (South Italy). J. Plant Pathol. 105, 987–1000. doi: 10.1007/s42161-023-01420-2

[B26] Han G. Yan H. Gu T. Cao L. Zhou Y. Liu W.. (2023). Identification of a wheat powdery mildew dominant resistance gene in the Pm5 locus for high-throughput marker-assisted selection. Plant Dis. 107, 450–456. doi: 10.1094/PDIS-07-22-1545-RE, PMID: 35815965

[B27] Huang M. Liu X. Zhou Y. Summers R. M. Zhang Z. (2019). BLINK: a package for the next level of genome-wide association studies with both individuals and markers in the millions. Gigascience 8. doi: 10.1093/gigascience/giy154, PMID: 30535326 PMC6365300

[B28] Hulin M. T. Mansfield J. W. Brain P. Xu X. Jackson R. W. Harrison R. J. (2018). Characterization of the pathogenicity of strains of Pseudomonas syringae towards cherry and plum. Plant Pathol. 67, 1177–1193. doi: 10.1111/ppa.12834, PMID: 29937581 PMC5993217

[B29] Koressaar T. Lepamets M. Kaplinski L. Raime K. Andreson R. Remm M. (2018). Primer3_masker: integrating masking of template sequence with primer design software | Bioinformatics | Oxford Academic. Bioinformatics 34, 1937–1938. doi: 10.1093/bioinformatics/bty036, PMID: 29360956

[B30] Lee K. Seo P. J. (2022). Wound-induced systemic responses and their coordination by electrical signals. Front. Plant Sci. 13. doi: 10.3389/fpls.2022.880680, PMID: 35665138 PMC9158525

[B31] León M. Berbegal M. Rodríguez-Reina J. M. Elena G. Abad-Campos P. Ramón-Albalat A.. (2020). Identification and characterization of diaporthe spp. associated with twig cankers and shoot blight of almonds in Spain. Agronomy 10, 1062. doi: 10.3390/agronomy10081062

[B32] Lin J.-S. Kuo C.-C. Yang I.-C. Tsai W.-A. Shen Y.-H. Lin C.-C.. (2018). MicroRNA160 modulates plant development and heat shock protein gene expression to mediate heat tolerance in Arabidopsis. Front. Plant Sci. 9. doi: 10.3389/fpls.2018.00068, PMID: 29449855 PMC5799662

[B33] Lipka A. E. Tian F. Wang Q. Peiffer J. Li M. Bradbury P. J.. (2012). GAPIT: genome association and prediction integrated tool. Bioinformatics 28, 2397–2399. doi: 10.1093/bioinformatics/bts444, PMID: 22796960

[B34] Luo Y. Ma R. Barrera E. Gusella G. Michailides T. J. (2022). Effects of temperature on development of canker-causing pathogens in almond and prune. Plant Dis. 106, 2424–2432. doi: 10.1094/PDIS-01-22-0048-RE, PMID: 35171640

[B35] Mancero-Castillo D. Espinoza L. Harmon P. F. Chaparro J. X. (2024). Botryosphaeriaceae infections on prunus germplasm: evaluation of pathogenicity, resistance loci, and detached assays of southeastern United States isolates. HortScience 59, 1700–1707. doi: 10.21273/HORTSCI18119-24

[B36] Martins M. Sarmento D. Oliveira M. M. Batlle I. Vargas F. J. (2002). Search for molecular markers linked to fusicoccum tolerance in almond. in. Acta Hortic. 577. doi: 10.17660/ActaHortic.2002.577.11

[B37] Martins M. Sarmento D. Oliveira M. M. Batlle I. Vargas F. J. (2005). “Development of SCAR-CPS markers linked to tolerance-sensitivity to Fusicoccum in almond,” in XIII GREMPA Meeting on Almonds and Pistachios. Eds. Oliveira M. M. Cordeiro V. (CIHEAM, Zaragoza), 187191.

[B38] Omodei F. (2007). Descrizione e caratterizzazione biometrica di cultivar Siciliane di mandorlo (P. Amygdalus L.) in conservazione ‘*ex situ.*’.

[B39] Opalski K. S. Schultheiss H. Kogel K. Hückelhoven R. (2005). The receptor-like MLO protein and the RAC/ROP family G-protein RACB modulate actin reorganization in barley attacked by the biotrophic powdery mildew fungus *Blumeria graminis* f.sp. *hordei* . Plant J. 41, 291–303. doi: 10.1111/j.1365-313X.2004.02292.x, PMID: 15634205

[B40] Ören E. Bayraktar H. (2025). Identifying fungi responsible for trunk and scaffold diseases in almonds in Türkiye. Physiol. Mol. Plant Pathol. 138, 102729. doi: 10.1016/j.pmpp.2025.102729

[B41] Ortega E. Sutherland B. G. Dicenta F. Boskovic R. Tobutt K. R. (2005). Determination of incompatibility genotypes in almond using first and second intron consensus primers: detection of new S alleles and correction of reported S genotypes. Plant Breed. 124, 188–196. doi: 10.1111/j.1439-0523.2004.01058.x

[B42] Pérez de los Cobos F. Coindre E. Dlalah N. Quilot-Turion B. Batlle I. Arús P.. (2023). Almond population genomics and non-additive GWAS reveal new insights into almond dissemination history and candidate genes for nut traits and blooming time. Hortic. Res. 10. doi: 10.1093/hr/uhad193, PMID: 37927408 PMC10623407

[B43] Pérez de los Cobos F. Martínez-García P. J. Romero A. Miarnau X. Eduardo I. Howad W.. (2021). Pedigree analysis of 220 almond genotypes reveals two world mainstream breeding lines based on only three different cultivars. Hortic. Res. 8, 11. doi: 10.1038/s41438-020-00444-4, PMID: 33384415 PMC7775440

[B44] R Core Team (2014). R: A language and environment for statistical computing (Vienna, Austria: R Found. Stat. Comput). Available online at: https://www.R-project.org/ (Accessed January 15, 2024).

[B45] Rehman A. Davik J. Karisto P. Kaseva J. Karhu S. Rantanen M.. (2025). A major QTL region associated with powdery mildew resistance in leaves and fruits of the reconstructed garden strawberry. Theor. Appl. Genet. 138, 93. doi: 10.1007/s00122-025-04871-6, PMID: 40195180 PMC11976356

[B46] Roth M. Serrie M. Segura V. Dlalah N. Cabel O. Malbot-Calonnec M.. (2024). Using non-additive effects in genome-wide association studies and genomic prediction to improve biotic stress tolerance in peach. in 7th International Conference of Quantitative Genetics. 213.

[B47] Ryan C. A. An G. (1988). Molecular biology of wound-inducible proteinase inhibitors in plants. Plant Cell Environ. 11, 345–349. doi: 10.1111/j.1365-3040.1988.tb01358.x

[B48] Sánchez-Pérez R. Howad W. Garcia-Mas J. Arús P. Martínez-Gómez P. Dicenta F. (2010). Molecular markers for kernel bitterness in almond. Tree Genet. Genomes 6, 237–245. doi: 10.1007/s11295-009-0244-7

[B49] Sánchez-Pérez R. Pavan S. Mazzeo R. Moldovan C. Aiese Cigliano R. Del Cueto J.. (2019). Mutation of a bHLH transcription factor allowed almond domestication. Sci. (80-.). 364, 1095–1098. doi: 10.1126/science.aav8197, PMID: 31197015

[B50] Schneider C. A. Rasband W. S. Eliceiri K. W. (2012). NIH Image to ImageJ: 25 years of image analysis. Nat. Methods 9, 671–675. doi: 10.1038/nmeth.2089, PMID: 22930834 PMC5554542

[B51] Sideli G. M. Mather D. Wirthensohn M. Dicenta F. Goonetilleke S. N. Martínez-García P. J.. (2023). Genome-wide association analysis and validation with KASP markers for nut and shell traits in almond (Prunus dulcis [Mill.] D.A.Webb). Tree Genet. Genomes 19, 13. doi: 10.1007/s11295-023-01588-9

[B52] Tamura M. Ushijima K. Sassa H. Hirano H. Tao R. Gradziel T. M.. (2000). Identification of self-incompatibility genotypes of almond by allele-specific PCR analysis. Theor. Appl. Genet. 101, 344–349. doi: 10.1007/s001220051489

[B53] Varjas V. Vajna L. Izsépi F. Nagy G. Pájtli (2017). First report of Phomopsis amygdali causing twig canker on almond in Hungary. Plant Dis. 101. doi: 10.1094/PDIS-03-17-0365-PDN

[B54] Walley J. W. Kelley D. R. Nestorova G. Hirschberg D. L. Dehesh K. (2010). Arabidopsis deadenylases atCAF1a and atCAF1b play overlapping and distinct roles in mediating environmental stress responses. Plant Physiol. 152, 866–875. doi: 10.1104/pp.109.149005, PMID: 19955262 PMC2815882

[B55] Wang Y. Tan B.-C. (2024). Pentatricopeptide repeat proteins in plants: Cellular functions, action mechanisms, and potential applications. Plant Commun. 6(2), 101203. doi: 10.1016/j.xplc.2024.101203, PMID: 39644091 PMC11897456

[B56] Wickham H. (2011). ggplot2. Wiley interdiscip. Rev. Comput. Stat. 3, 180–185. doi: 10.1002/wics.147

[B57] Willcox G. Fornite S. Herveux L. (2008). Early Holocene cultivation before domestication in northern Syria. Veg. Hist. Archaeobot. 17, 313–325. doi: 10.1007/s00334-007-0121-y

[B58] Zhang A. Xiong Y. Liu F. Zhang X. (2023). A genome-wide analysis of the pentatricopeptide repeat protein gene family in two kiwifruit species with an emphasis on the role of RNA editing in pathogen stress. Int. J. Mol. Sci. 24(18), 13700. doi: 10.3390/ijms241813700, PMID: 37762001 PMC10530749

