# Supplementary material for: Exploring additive and non-additive genetic models to decipher the genetic regulation of almond tolerance to Diaporthe amygdali
Source: Front Plant Sci. 2025 Sep 19;16:1608958. doi: 10.3389/fpls.2025.1608958 (PMC12491216; doi:10.3389/fpls.2025.1608958)
Supplement: Supplementary Table 1 — Almond varieties used in this study with results related to the detached-twig assay. Asterisks indicate those varieties used also in the in planta assay. [file DataSheet1.docx]

Supplementary Material

# Supplementary Figures and Tables

**
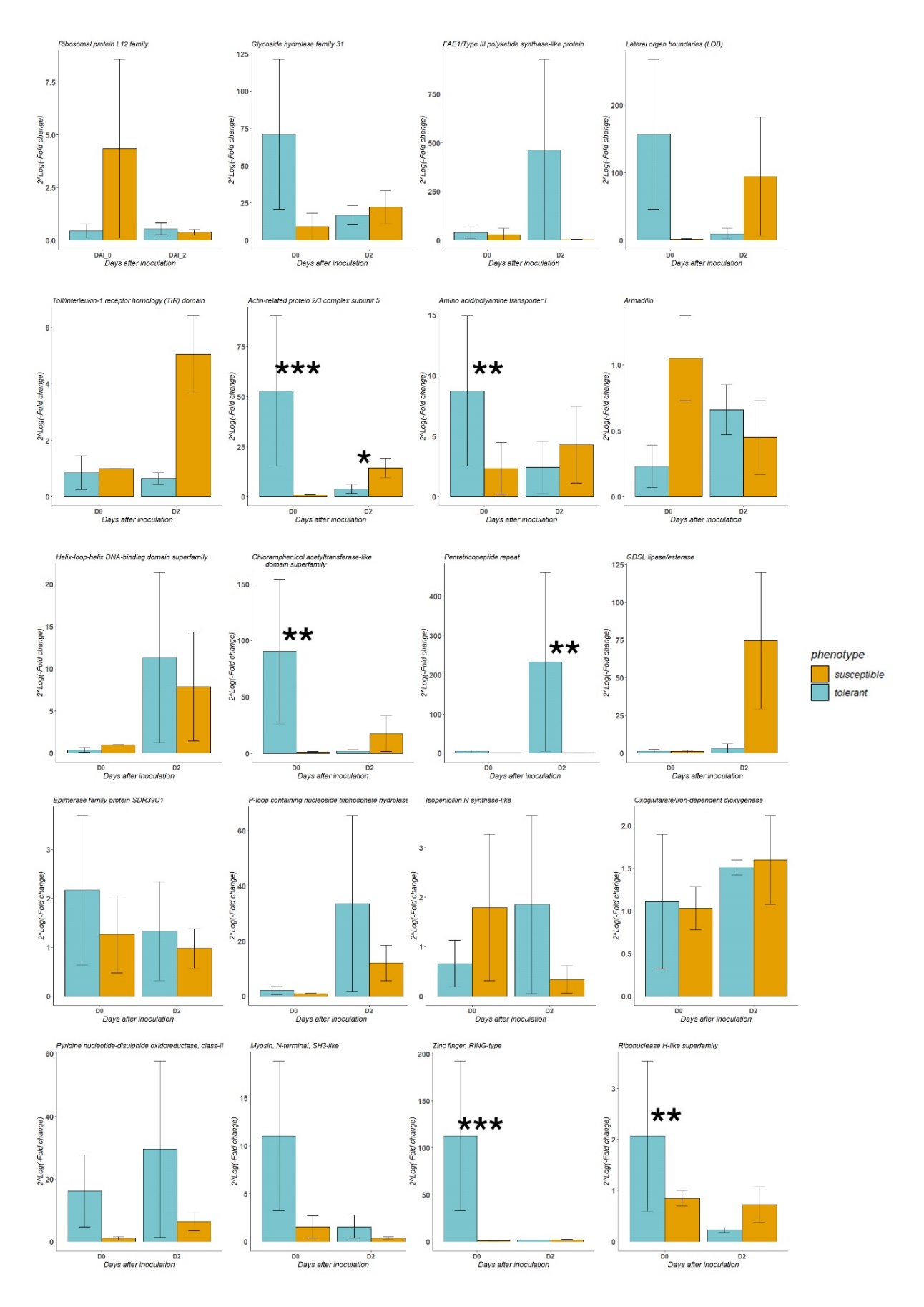
**

**Supplementary Figure 1.** Results of RT-qPCR analysis conducted on the twenty candidate genes identified through the GWAS analysis.

**Supplementary Table 1.** Almond varieties used in this study with results related to the detached-twig assay. Asterisks indicate those varieties used also in the *in planta* assay.

| **Variety** | **Origin** | **mean necrotic lesion lenght (cm)** | **standard deviation (cm)** |
| --- | --- | --- | --- |
| Acquaviva | Sicily | 2.32 | 0.21 |
| Albanisa | Sicily | 1.49 | 1.01 |
| Amara (Ispica) | Sicily | 2.21 | 1.16 |
| Amara Di Martorana | Sicily | 3.16 | 1.11 |
| Angelica | Sicily | 1.48 | 0.22 |
| Baggiana | Sicily | 1.52 | 0.16 |
| Bargellera | Sicily | 2.06 | 0.48 |
| Bari Flores | Italy (Apulia) | 1.08 | 0.06 |
| Bari Rachela | Italy (Apulia) | 1.39 | 0.57 |
| Bari Sabittisa | Italy (Apulia) | 1.38 | 0.33 |
| Barunissa | Sicily | 2.00 | 0.30 |
| Belvedere | Sicily | 2.17 | 0.23 |
| Bennici* | Sicily | 1.10 | 0.13 |
| Bianculidda Di Pezzino | Sicily | 3.39 | 2.24 |
| Bottara | Sicily | 2.23 | 1.05 |
| Bronte 1 | Sicily | 2.03 | 0.23 |
| Buscemi | Sicily | 2.10 | 0.21 |
| Cacciatura | Sicily | 1.51 | 0.12 |
| Cacinova | Sicily | 1.54 | 1.16 |
| Calamonaci | Sicily | 1.01 | 0.28 |
| Caluriedda | Sicily | 2.24 | 0.37 |
| Canicattinisa | Sicily | 3.25 | 1.14 |
| Carrubina | Sicily | 1.43 | 0.31 |
| Castrianisa | Sicily | 2.19 | 0.35 |
| Catrubba | Sicily | 2.13 | 0.40 |
| Cavalera Di Naro | Sicily | 1.21 | 0.13 |
| Cavaliere | Sicily | 1.43 | 0.17 |
| Cesarò 1 | Sicily | 1.57 | 1.24 |
| Chiarchiara | Sicily | 2.03 | 0.28 |
| Chiatta | Sicily | 1.20 | 0.22 |
| Chiatta | Sicily | 1.36 | 0.36 |
| Chiricupara | Sicily | 2.16 | 0.44 |
| Comunista | Sicily | 1.28 | 0.02 |
| Cumma (SOAT Licata) | Sicily | 2.02 | 0.56 |
| Cuore | Sicily | 1.09 | 0.41 |
| Cuti* | Sicily | 1.36 | 0.07 |
| Di Giorgio | Sicily | 2.00 | 0.12 |
| Don Filippo | Sicily | 1.47 | 0.18 |
| Don Pe’ | Sicily | 3.26 | 1.38 |
| Don Peppino | Sicily | 1.41 | 0.39 |
| Don Vincenzo | Sicily | 3.13 | 0.57 |
| Enna 2 | Sicily | 1.53 | 0.46 |
| Falsa Pizzuta | Sicily | 2.21 | 0.54 |
| Falsa Pizzuta | Sicily | 2.31 | 1.45 |
| Fascionello* | Sicily | 3.36 | 3.09 |
| Fastuchina | Sicily | 1.32 | 0.13 |
| Favarò 1 | Sicily | 1.51 | 0.31 |
| Favarò 2 | Sicily | 1.23 | 0.10 |
| Fellamasa Casteltermini | Sicily | 2.39 | 1.25 |
| Ferraduel* | International (France) | 5.23 | 1.10 |
| Ferragnes* | International (Spain) | 1.19 | 0.23 |
| Filippazzo | Sicily | 1.36 | 0.05 |
| Filippo Ceo | Italy (Apulia) | 1.29 | 0.27 |
| Gaglio | Sicily | 2.02 | 0.40 |
| Genco | Italy (Apulia) | 1.28 | 0.28 |
| Giafaglione* | Sicily | 1.38 | 0.03 |
| Giardinella | Sicily | 1.56 | 0.45 |
| Griddetta | Sicily | 1.26 | 0.12 |
| Gruttisa | Sicily | 1.56 | 0.42 |
| Laurenne* | International (France) | 1.49 | 0.24 |
| Lisciannarisa | Sicily | 1.56 | 0.15 |
| Lumia | Sicily | 1.28 | 1.06 |
| Mallia | Sicily | 1.37 | 0.40 |
| Mannara Di Chianu | Sicily | 2.23 | 1.05 |
| Marra' (MENNULA DI) | Sicily | 1.29 | 0.12 |
| Mastraciccia | Sicily | 1.53 | 0.12 |
| Mennula Du Nigliu | Sicily | 1.49 | 0.43 |
| Mennula Du Vattiu | Sicily | 1.09 | 0.39 |
| Mezzalira | Sicily | 2.18 | 0.32 |
| Milocca | Sicily | 2.11 | 0.35 |
| Mirabile | Sicily | 2.27 | 0.45 |
| Miricanedda | Sicily | 1.38 | 0.38 |
| Miuzza | Sicily | 1.09 | 0.27 |
| Montagna | Sicily | 1.05 | 0.07 |
| Mullisa Grande | Sicily | 1.43 | 0.24 |
| Mullisa Piccola | Sicily | 1.19 | 0.38 |
| Nambaredda | Sicily | 2.26 | 1.09 |
| Naro 1 | Sicily | 1.50 | 0.40 |
| Nerone | Sicily | 1.41 | 0.41 |
| Nivera Manza | Sicily | 1.22 | 0.25 |
| Nuciddara | Sicily | 3.16 | 1.09 |
| Palma | Sicily | 1.31 | 0.19 |
| Perciavisazza | Sicily | 1.35 | 1.04 |
| Persichina | Sicily | 1.49 | 0.23 |
| Piatta Mollisa | Sicily | 1.20 | 0.24 |
| Pilusedda | Sicily | 1.35 | 0.42 |
| Piricuddara | Sicily | 2.24 | 0.52 |
| Pizzuta (Bronte)* | Sicily | 3.49 | 1.01 |
| Pizzuta Contino | Sicily | 2.03 | 0.11 |
| Pizzuta D’Avola | Sicily | 2.38 | 0.51 |
| Pizzuta Grande | Sicily | 2.25 | 0.10 |
| Pizzutedda | Sicily | 1.35 | 0.15 |
| Pizzutella | Sicily | 1.57 | 0.17 |
| Rapparina | Sicily | 2.23 | 0.40 |
| Rapparina | Sicily | 2.10 | 1.27 |
| Regina | Sicily | 2.20 | 1.06 |
| Reginella | Sicily | 2.22 | 1.26 |
| Romana | Sicily | 1.02 | 0.07 |
| Sancisuca | Sicily | 3.16 | 2.32 |
| Sancisuca (Racalmuto) | Sicily | 2.17 | 0.36 |
| Sarbaggedda* | Sicily | 1.18 | 0.41 |
| Sarbaggia Di Patito | Sicily | 1.38 | 0.17 |
| Sarbaggia Di Sciascia | Sicily | 2.19 | 1.14 |
| Sarbaggia Di Vitello | Sicily | 1.39 | 0.05 |
| Scummissa | Sicily | 1.18 | 0.22 |
| Selvatica Favata | Sicily | 2.12 | 0.52 |
| Staccia | Sicily | 1.11 | 0.26 |
| Supernova* | Italy (Apulia) | 1.55 | 0.30 |
| Tabacchina | Sicily | 1.47 | 0.23 |
| Texas* | International (USA) | 2.14 | 0.27 |
| Tricula | Sicily | 1.56 | 0.19 |
| Tunnulidda | Sicily | 2.26 | 1.02 |
| Tuono* | Italy (Apulia) | 1.49 | 0.18 |
| Universo | Italy (Apulia) | 1.31 | 0.10 |
| Uova Di Cucco | Sicily | 1.38 | 0.26 |
| Vaiana (mennula di) | Sicily | 2.14 | 0.37 |
| Villana | Sicily | 3.01 | 1.03 |
| Vinci A Tutti | Sicily | 2.16 | 0.32 |
| Zaccaneddara | Sicily | 1.48 | 0.39 |
| Zagarri’ | Sicily | 1.41 | 0.23 |
| Zammuto 2 | Sicily | 2.25 | 1.14 |
| Barbara | Sicily | 1.04 | 0.37 |
| Zottafunnuta | Sicily | 1.58 | 0.49 |

**Supplementary Table 2.** Primer designed for the RT-qPCR performed in this study.

| **Target** | **Primer Forward** | **Primer Reverse** | **Amplicon** |
| --- | --- | --- | --- |
| Ribosomal protein L12 family | TTCCTTCTTCTCCTCGGCTG | CCGTCTTCCAGTGATCGTTG | 118 |
| Glycoside hydrolase family 31 | CCCCTCTTGCAGCTCTATGT | GGAACCTCCCATCTCTGCTT | 88 |
| FAE1/Type III polyketide synthase-like protein | CGACGTGCCAAATACCGATT | TTTGCCCTCCTTGTCCTCTT | 102 |
| Lateral organ boundaries, LOB | TTCACAATCCGTCCACAAGC | CTCAACCTCATCAATGCCGG | 85 |
| Toll/interleukin-1 receptor homology (TIR) domain | CTCTTCAGTTGGCACGCTTT | TCCGCAATGATCGACTAGCT | 84 |
| Actin-related protein 2/3 complex subunit 5 | CCAATCGGAAAAGACCTGGG | CGACACCAAAACGACACAGT | 80 |
| Amino acid/polyamine transporter I | GGACAAGAAAACCCTGGCAG | TGGTAGCTGTAGTTGTGGCA | 84 |
| Armadillo | AATCTTGGAGGGAAGGGCTC | TCCTCCTTCCTCAGCAACAG | 97 |
| Helix-loop-helix DNA-binding domain superfamily | CGCTAATCCGCTCTCTACGA | AGAAGCCCAAACCCAAGTCA | 80 |
| Chloramphenicol acetyltransferase-like domain superfamily | CAGAGAGTTCGTTGCGGATG | ATACCCACCTTTTCCCCACC | 94 |
| Pentatricopeptide repeat | GGAAAGCCTGGCAGAGACTA | CTAAAGAACTTCACGGCGGG | 102 |
| GDSL lipase/esterase | GTTGCAAAGTGACCTCCCAG | ACACATTTCTCGCCCTGTTC | 89 |
| Epimerase family protein SDR39U1 | TACCGATAAAACCCGTGGCT | GTCTCTCTGCCCCTCTCAAG | 119 |
| P-loop containing nucleoside triphosphate hydrolase | CGCAATCACTCGCCTCTAAC | GGAGCTTTTCTTGCATGGCT | 86 |
| Isopenicillin N synthase-like | CTACTCCCTCTACAAGCCGG | CCAACCCCAAGCTTTCTGAG | 113 |
| Oxoglutarate/iron-dependent dioxygenase | CCCTCCCAACGGTTTCTTTG | TTGTTGTTCAAGGTGGCTCG | 98 |
| Pyridine nucleotide-disulphide oxidoreductase, class-II | TCCTTTCCAGACCTTGCCAA | GCAGTTGGGTTCACAAGGAG | 87 |
| Myosin, N-terminal, SH3-like | CCTTCTCTTGCAGGGACGTA | CGAGGACTCCAACAATGCTG | 100 |
| Zinc finger, RING-type | ATGGCATCCTTCGTCACCTT | GCCTTGCAAGCACAAATTCC | 111 |
| Ribonuclease H-like superfamily | GAGCCACCAACAGATCAACG | GGAAAGGAGCCTAAGGACGT | 120 |

**Supplementary Table 3.** List of genes resulted from the gene ontology analysis and annotation of those sequences putatively involved in the control of the tolerance/susceptibility to *D. amygdali*.

| **prudul_name** | **SNP impact** | **V1** | **V4** | **V5** | **trait** | **snp** | **GO_code** | **GO** | **kegg_ort_code** | **kegg_ort_description** | **ipr_code** | **ipr_description** |
| --- | --- | --- | --- | --- | --- | --- | --- | --- | --- | --- | --- | --- |
| **Prudul26A005898T1** |  | Pd01 | 5878274 | 5879586 | recessive | AX-586016477 | GO:0005840 | Cellular Component:ribosome | K02942 | large subunit ribosomal protein LP1 | IPR027534 | Ribosomal protein L12 family |
|  |  | Pd01 | 5878274 | 5879586 | recessive | AX-586016477 | GO:0005622 | Cellular Component:intracellular | K02942 | large subunit ribosomal protein LP1 | IPR027534 | Ribosomal protein L12 family |
|  |  | Pd01 | 5878274 | 5879586 | recessive | AX-586016477 | GO:0003735 | Molecular Function:structural constituent of ribosome | K02942 | large subunit ribosomal protein LP1 | IPR027534 | Ribosomal protein L12 family |
|  |  | Pd01 | 5878274 | 5879586 | recessive | AX-586016477 | GO:0006414 | Biological Process:translational elongation | K02942 | large subunit ribosomal protein LP1 | IPR027534 | Ribosomal protein L12 family |
| **Prudul26A021841T1** |  | Pd01 | 5889805 | 5893981 | recessive | AX-586016477 | NA | NA | NA | NA | IPR036850 | Nucleoside diphosphate kinase-like domain superfamily |
|  |  | Pd01 | 5889805 | 5893981 | recessive | AX-586016477 | NA | NA | NA | NA | IPR039641 | Low-molecular-weight cysteine-rich protein |
| **Prudul26A021841T2** |  | Pd01 | 5889807 | 5893981 | recessive | AX-586016477 | NA | NA | NA | NA | IPR039641 | Low-molecular-weight cysteine-rich protein |
|  |  | Pd01 | 5889807 | 5893981 | recessive | AX-586016477 | NA | NA | NA | NA | IPR036850 | Nucleoside diphosphate kinase-like domain superfamily |
| **Prudul26A028947T1** |  | Pd01 | 5895636 | 5897000 | recessive | AX-586016477 | GO:0016747 | Molecular Function:transferase activity, transferring acyl groups other than amino-acyl groups | K13065 | shikimate O-hydroxycinnamoyltransferase [EC:2.3.1.133] | IPR003480 | Transferase |
|  |  | Pd01 | 5895636 | 5897000 | recessive | AX-586016477 | GO:0016747 | Molecular Function:transferase activity, transferring acyl groups other than amino-acyl groups | K13065 | shikimate O-hydroxycinnamoyltransferase [EC:2.3.1.133] | IPR023213 | Chloramphenicol acetyltransferase-like domain superfamily |
| **Prudul26A025778T1** |  | Pd01 | 5912561 | 5917867 | recessive | AX-586016477 | GO:0008168 | Molecular Function:methyltransferase activity | K06127 | 2-methoxy-6-polyprenyl-1,4-benzoquinol methylase [EC:2.1.1.201] | IPR023576 | UbiE/COQ5 methyltransferase, conserved site |
|  |  | Pd01 | 5912561 | 5917867 | recessive | AX-586016477 | GO:0008168 | Molecular Function:methyltransferase activity | K06127 | 2-methoxy-6-polyprenyl-1,4-benzoquinol methylase [EC:2.1.1.201] | IPR004033 | UbiE/COQ5 methyltransferase |
|  |  | Pd01 | 5912561 | 5917867 | recessive | AX-586016477 | GO:0008168 | Molecular Function:methyltransferase activity | K06127 | 2-methoxy-6-polyprenyl-1,4-benzoquinol methylase [EC:2.1.1.201] | IPR029063 | S-adenosyl-L-methionine-dependent methyltransferase |
| **Prudul26A004071T1** | Downstream gene variant | Pd01 | 5919339 | 5933508 | recessive | AX-586016477 | GO:0005634 | Cellular Component:nucleus | NA | NA | IPR010678 | Digestive organ expansion factor, predicted |
| **Prudul26A015002T1** | Upstream gene variant | Pd01 | 5938376 | 5939129 | recessive | AX-586016477 | GO:0008233 | Molecular Function:peptidase activity | K01369 | legumain [EC:3.4.22.34] | IPR001096 | Peptidase C13, legumain |
|  |  | Pd01 | 5938376 | 5939129 | recessive | AX-586016477 | GO:0006508 | Biological Process:proteolysis | K01369 | legumain [EC:3.4.22.34] | IPR001096 | Peptidase C13, legumain |
| **Prudul26A015002T2** | Upstream gene variant | Pd01 | 5938609 | 5938864 | recessive | AX-586016477 | GO:0006508 | Biological Process:proteolysis | K01369 | legumain [EC:3.4.22.34] | IPR001096 | Peptidase C13, legumain |
|  |  | Pd01 | 5938609 | 5938864 | recessive | AX-586016477 | GO:0008233 | Molecular Function:peptidase activity | K01369 | legumain [EC:3.4.22.34] | IPR001096 | Peptidase C13, legumain |
| **Prudul26A018177T1** |  | Pd01 | 5945587 | 5946218 | recessive | AX-586016477 | NA | NA | K07466 | replication factor A1 | IPR012340 | Nucleic acid-binding, OB-fold |
| **Prudul26A027440T1** |  | Pd01 | 5960560 | 5962238 | recessive | AX-586016477 | NA | NA | NA | NA | IPR004252 | Probable transposase, Ptta/En/Spm, plant |
| **Prudul26A004885T1** |  | Pd01 | 5971404 | 5971557 | recessive | AX-586016477 | NA | NA | K05391 | cyclic nucleotide gated channel, plant | NA | NA |
| **Prudul26A010600T1** |  | Pd01 | 5974999 | 5975356 | recessive | AX-586016477 | NA | NA | NA | NA | NA | NA |
| **Prudul26A003924T1** |  | Pd01 | 5990909 | 5992303 | recessive | AX-586016477 | GO:0016747 | Molecular Function:transferase activity, transferring acyl groups other than amino-acyl groups | K13065 | shikimate O-hydroxycinnamoyltransferase [EC:2.3.1.133] | IPR023213 | Chloramphenicol acetyltransferase-like domain superfamily |
|  |  | Pd01 | 5990909 | 5992303 | recessive | AX-586016477 | GO:0016747 | Molecular Function:transferase activity, transferring acyl groups other than amino-acyl groups | K13065 | shikimate O-hydroxycinnamoyltransferase [EC:2.3.1.133] | IPR003480 | Transferase |
| **Prudul26A011296T1** |  | Pd01 | 2,70E+07 | 2,70E+07 | recessive | AX-586027373 | GO:0005515 | Molecular Function:protein binding | K10839 | UV excision repair protein RAD23 | IPR029071 | Ubiquitin-like domain superfamily |
|  |  | Pd01 | 2,70E+07 | 2,70E+07 | recessive | AX-586027373 | GO:0005515 | Molecular Function:protein binding | K10839 | UV excision repair protein RAD23 | IPR036353 | XPC-binding domain superfamily |
|  |  | Pd01 | 2,70E+07 | 2,70E+07 | recessive | AX-586027373 | GO:0005515 | Molecular Function:protein binding | K10839 | UV excision repair protein RAD23 | IPR015940 | Ubiquitin-associated domain |
|  |  | Pd01 | 2,70E+07 | 2,70E+07 | recessive | AX-586027373 | GO:0005515 | Molecular Function:protein binding | K10839 | UV excision repair protein RAD23 | IPR015360 | XPC-binding domain |
|  |  | Pd01 | 2,70E+07 | 2,70E+07 | recessive | AX-586027373 | GO:0005515 | Molecular Function:protein binding | K10839 | UV excision repair protein RAD23 | IPR004806 | UV excision repair protein Rad23 |
|  |  | Pd01 | 2,70E+07 | 2,70E+07 | recessive | AX-586027373 | GO:0005515 | Molecular Function:protein binding | K10839 | UV excision repair protein RAD23 | IPR009060 | UBA-like superfamily |
|  |  | Pd01 | 2,70E+07 | 2,70E+07 | recessive | AX-586027373 | GO:0005515 | Molecular Function:protein binding | K10839 | UV excision repair protein RAD23 | IPR000626 | Ubiquitin domain |
|  |  | Pd01 | 2,70E+07 | 2,70E+07 | recessive | AX-586027373 | GO:0006289 | Biological Process:nucleotide-excision repair | K10839 | UV excision repair protein RAD23 | IPR029071 | Ubiquitin-like domain superfamily |
|  |  | Pd01 | 2,70E+07 | 2,70E+07 | recessive | AX-586027373 | GO:0006289 | Biological Process:nucleotide-excision repair | K10839 | UV excision repair protein RAD23 | IPR036353 | XPC-binding domain superfamily |
|  |  | Pd01 | 2,70E+07 | 2,70E+07 | recessive | AX-586027373 | GO:0006289 | Biological Process:nucleotide-excision repair | K10839 | UV excision repair protein RAD23 | IPR015940 | Ubiquitin-associated domain |
|  |  | Pd01 | 2,70E+07 | 2,70E+07 | recessive | AX-586027373 | GO:0006289 | Biological Process:nucleotide-excision repair | K10839 | UV excision repair protein RAD23 | IPR015360 | XPC-binding domain |
|  |  | Pd01 | 2,70E+07 | 2,70E+07 | recessive | AX-586027373 | GO:0006289 | Biological Process:nucleotide-excision repair | K10839 | UV excision repair protein RAD23 | IPR004806 | UV excision repair protein Rad23 |
|  |  | Pd01 | 2,70E+07 | 2,70E+07 | recessive | AX-586027373 | GO:0006289 | Biological Process:nucleotide-excision repair | K10839 | UV excision repair protein RAD23 | IPR009060 | UBA-like superfamily |
|  |  | Pd01 | 2,70E+07 | 2,70E+07 | recessive | AX-586027373 | GO:0006289 | Biological Process:nucleotide-excision repair | K10839 | UV excision repair protein RAD23 | IPR000626 | Ubiquitin domain |
|  |  | Pd01 | 2,70E+07 | 2,70E+07 | recessive | AX-586027373 | GO:0043161 | Biological Process:proteasome-mediated ubiquitin-dependent protein catabolic process | K10839 | UV excision repair protein RAD23 | IPR029071 | Ubiquitin-like domain superfamily |
|  |  | Pd01 | 2,70E+07 | 2,70E+07 | recessive | AX-586027373 | GO:0043161 | Biological Process:proteasome-mediated ubiquitin-dependent protein catabolic process | K10839 | UV excision repair protein RAD23 | IPR036353 | XPC-binding domain superfamily |
|  |  | Pd01 | 2,70E+07 | 2,70E+07 | recessive | AX-586027373 | GO:0043161 | Biological Process:proteasome-mediated ubiquitin-dependent protein catabolic process | K10839 | UV excision repair protein RAD23 | IPR015940 | Ubiquitin-associated domain |
|  |  | Pd01 | 2,70E+07 | 2,70E+07 | recessive | AX-586027373 | GO:0043161 | Biological Process:proteasome-mediated ubiquitin-dependent protein catabolic process | K10839 | UV excision repair protein RAD23 | IPR015360 | XPC-binding domain |
|  |  | Pd01 | 2,70E+07 | 2,70E+07 | recessive | AX-586027373 | GO:0043161 | Biological Process:proteasome-mediated ubiquitin-dependent protein catabolic process | K10839 | UV excision repair protein RAD23 | IPR004806 | UV excision repair protein Rad23 |
|  |  | Pd01 | 2,70E+07 | 2,70E+07 | recessive | AX-586027373 | GO:0043161 | Biological Process:proteasome-mediated ubiquitin-dependent protein catabolic process | K10839 | UV excision repair protein RAD23 | IPR009060 | UBA-like superfamily |
|  |  | Pd01 | 2,70E+07 | 2,70E+07 | recessive | AX-586027373 | GO:0043161 | Biological Process:proteasome-mediated ubiquitin-dependent protein catabolic process | K10839 | UV excision repair protein RAD23 | IPR000626 | Ubiquitin domain |
|  |  | Pd01 | 2,70E+07 | 2,70E+07 | recessive | AX-586027373 | GO:0003684 | Molecular Function:damaged DNA binding | K10839 | UV excision repair protein RAD23 | IPR029071 | Ubiquitin-like domain superfamily |
|  |  | Pd01 | 2,70E+07 | 2,70E+07 | recessive | AX-586027373 | GO:0003684 | Molecular Function:damaged DNA binding | K10839 | UV excision repair protein RAD23 | IPR036353 | XPC-binding domain superfamily |
|  |  | Pd01 | 2,70E+07 | 2,70E+07 | recessive | AX-586027373 | GO:0003684 | Molecular Function:damaged DNA binding | K10839 | UV excision repair protein RAD23 | IPR015940 | Ubiquitin-associated domain |
|  |  | Pd01 | 2,70E+07 | 2,70E+07 | recessive | AX-586027373 | GO:0003684 | Molecular Function:damaged DNA binding | K10839 | UV excision repair protein RAD23 | IPR015360 | XPC-binding domain |
|  |  | Pd01 | 2,70E+07 | 2,70E+07 | recessive | AX-586027373 | GO:0003684 | Molecular Function:damaged DNA binding | K10839 | UV excision repair protein RAD23 | IPR004806 | UV excision repair protein Rad23 |
|  |  | Pd01 | 2,70E+07 | 2,70E+07 | recessive | AX-586027373 | GO:0003684 | Molecular Function:damaged DNA binding | K10839 | UV excision repair protein RAD23 | IPR009060 | UBA-like superfamily |
|  |  | Pd01 | 2,70E+07 | 2,70E+07 | recessive | AX-586027373 | GO:0003684 | Molecular Function:damaged DNA binding | K10839 | UV excision repair protein RAD23 | IPR000626 | Ubiquitin domain |
|  |  | Pd01 | 2,70E+07 | 2,70E+07 | recessive | AX-586027373 | GO:0005634 | Cellular Component:nucleus | K10839 | UV excision repair protein RAD23 | IPR029071 | Ubiquitin-like domain superfamily |
|  |  | Pd01 | 2,70E+07 | 2,70E+07 | recessive | AX-586027373 | GO:0005634 | Cellular Component:nucleus | K10839 | UV excision repair protein RAD23 | IPR036353 | XPC-binding domain superfamily |
|  |  | Pd01 | 2,70E+07 | 2,70E+07 | recessive | AX-586027373 | GO:0005634 | Cellular Component:nucleus | K10839 | UV excision repair protein RAD23 | IPR015940 | Ubiquitin-associated domain |
|  |  | Pd01 | 2,70E+07 | 2,70E+07 | recessive | AX-586027373 | GO:0005634 | Cellular Component:nucleus | K10839 | UV excision repair protein RAD23 | IPR015360 | XPC-binding domain |
|  |  | Pd01 | 2,70E+07 | 2,70E+07 | recessive | AX-586027373 | GO:0005634 | Cellular Component:nucleus | K10839 | UV excision repair protein RAD23 | IPR004806 | UV excision repair protein Rad23 |
|  |  | Pd01 | 2,70E+07 | 2,70E+07 | recessive | AX-586027373 | GO:0005634 | Cellular Component:nucleus | K10839 | UV excision repair protein RAD23 | IPR009060 | UBA-like superfamily |
|  |  | Pd01 | 2,70E+07 | 2,70E+07 | recessive | AX-586027373 | GO:0005634 | Cellular Component:nucleus | K10839 | UV excision repair protein RAD23 | IPR000626 | Ubiquitin domain |
| **Prudul26A011296T2** |  | Pd01 | 2,70E+07 | 2,70E+07 | recessive | AX-586027373 | GO:0005634 | Cellular Component:nucleus | K10839 | UV excision repair protein RAD23 | IPR029071 | Ubiquitin-like domain superfamily |
|  |  | Pd01 | 2,70E+07 | 2,70E+07 | recessive | AX-586027373 | GO:0005634 | Cellular Component:nucleus | K10839 | UV excision repair protein RAD23 | IPR015940 | Ubiquitin-associated domain |
|  |  | Pd01 | 2,70E+07 | 2,70E+07 | recessive | AX-586027373 | GO:0005634 | Cellular Component:nucleus | K10839 | UV excision repair protein RAD23 | IPR036353 | XPC-binding domain superfamily |
|  |  | Pd01 | 2,70E+07 | 2,70E+07 | recessive | AX-586027373 | GO:0005634 | Cellular Component:nucleus | K10839 | UV excision repair protein RAD23 | IPR009060 | UBA-like superfamily |
|  |  | Pd01 | 2,70E+07 | 2,70E+07 | recessive | AX-586027373 | GO:0005634 | Cellular Component:nucleus | K10839 | UV excision repair protein RAD23 | IPR000626 | Ubiquitin domain |
|  |  | Pd01 | 2,70E+07 | 2,70E+07 | recessive | AX-586027373 | GO:0005634 | Cellular Component:nucleus | K10839 | UV excision repair protein RAD23 | IPR015360 | XPC-binding domain |
|  |  | Pd01 | 2,70E+07 | 2,70E+07 | recessive | AX-586027373 | GO:0005634 | Cellular Component:nucleus | K10839 | UV excision repair protein RAD23 | IPR004806 | UV excision repair protein Rad23 |
|  |  | Pd01 | 2,70E+07 | 2,70E+07 | recessive | AX-586027373 | GO:0043161 | Biological Process:proteasome-mediated ubiquitin-dependent protein catabolic process | K10839 | UV excision repair protein RAD23 | IPR029071 | Ubiquitin-like domain superfamily |
|  |  | Pd01 | 2,70E+07 | 2,70E+07 | recessive | AX-586027373 | GO:0043161 | Biological Process:proteasome-mediated ubiquitin-dependent protein catabolic process | K10839 | UV excision repair protein RAD23 | IPR015940 | Ubiquitin-associated domain |
|  |  | Pd01 | 2,70E+07 | 2,70E+07 | recessive | AX-586027373 | GO:0043161 | Biological Process:proteasome-mediated ubiquitin-dependent protein catabolic process | K10839 | UV excision repair protein RAD23 | IPR036353 | XPC-binding domain superfamily |
|  |  | Pd01 | 2,70E+07 | 2,70E+07 | recessive | AX-586027373 | GO:0043161 | Biological Process:proteasome-mediated ubiquitin-dependent protein catabolic process | K10839 | UV excision repair protein RAD23 | IPR009060 | UBA-like superfamily |
|  |  | Pd01 | 2,70E+07 | 2,70E+07 | recessive | AX-586027373 | GO:0043161 | Biological Process:proteasome-mediated ubiquitin-dependent protein catabolic process | K10839 | UV excision repair protein RAD23 | IPR000626 | Ubiquitin domain |
|  |  | Pd01 | 2,70E+07 | 2,70E+07 | recessive | AX-586027373 | GO:0043161 | Biological Process:proteasome-mediated ubiquitin-dependent protein catabolic process | K10839 | UV excision repair protein RAD23 | IPR015360 | XPC-binding domain |
|  |  | Pd01 | 2,70E+07 | 2,70E+07 | recessive | AX-586027373 | GO:0043161 | Biological Process:proteasome-mediated ubiquitin-dependent protein catabolic process | K10839 | UV excision repair protein RAD23 | IPR004806 | UV excision repair protein Rad23 |
|  |  | Pd01 | 2,70E+07 | 2,70E+07 | recessive | AX-586027373 | GO:0005515 | Molecular Function:protein binding | K10839 | UV excision repair protein RAD23 | IPR029071 | Ubiquitin-like domain superfamily |
|  |  | Pd01 | 2,70E+07 | 2,70E+07 | recessive | AX-586027373 | GO:0005515 | Molecular Function:protein binding | K10839 | UV excision repair protein RAD23 | IPR015940 | Ubiquitin-associated domain |
|  |  | Pd01 | 2,70E+07 | 2,70E+07 | recessive | AX-586027373 | GO:0005515 | Molecular Function:protein binding | K10839 | UV excision repair protein RAD23 | IPR036353 | XPC-binding domain superfamily |
|  |  | Pd01 | 2,70E+07 | 2,70E+07 | recessive | AX-586027373 | GO:0005515 | Molecular Function:protein binding | K10839 | UV excision repair protein RAD23 | IPR009060 | UBA-like superfamily |
|  |  | Pd01 | 2,70E+07 | 2,70E+07 | recessive | AX-586027373 | GO:0005515 | Molecular Function:protein binding | K10839 | UV excision repair protein RAD23 | IPR000626 | Ubiquitin domain |
|  |  | Pd01 | 2,70E+07 | 2,70E+07 | recessive | AX-586027373 | GO:0005515 | Molecular Function:protein binding | K10839 | UV excision repair protein RAD23 | IPR015360 | XPC-binding domain |
|  |  | Pd01 | 2,70E+07 | 2,70E+07 | recessive | AX-586027373 | GO:0005515 | Molecular Function:protein binding | K10839 | UV excision repair protein RAD23 | IPR004806 | UV excision repair protein Rad23 |
|  |  | Pd01 | 2,70E+07 | 2,70E+07 | recessive | AX-586027373 | GO:0003684 | Molecular Function:damaged DNA binding | K10839 | UV excision repair protein RAD23 | IPR029071 | Ubiquitin-like domain superfamily |
|  |  | Pd01 | 2,70E+07 | 2,70E+07 | recessive | AX-586027373 | GO:0003684 | Molecular Function:damaged DNA binding | K10839 | UV excision repair protein RAD23 | IPR015940 | Ubiquitin-associated domain |
|  |  | Pd01 | 2,70E+07 | 2,70E+07 | recessive | AX-586027373 | GO:0003684 | Molecular Function:damaged DNA binding | K10839 | UV excision repair protein RAD23 | IPR036353 | XPC-binding domain superfamily |
|  |  | Pd01 | 2,70E+07 | 2,70E+07 | recessive | AX-586027373 | GO:0003684 | Molecular Function:damaged DNA binding | K10839 | UV excision repair protein RAD23 | IPR009060 | UBA-like superfamily |
|  |  | Pd01 | 2,70E+07 | 2,70E+07 | recessive | AX-586027373 | GO:0003684 | Molecular Function:damaged DNA binding | K10839 | UV excision repair protein RAD23 | IPR000626 | Ubiquitin domain |
|  |  | Pd01 | 2,70E+07 | 2,70E+07 | recessive | AX-586027373 | GO:0003684 | Molecular Function:damaged DNA binding | K10839 | UV excision repair protein RAD23 | IPR015360 | XPC-binding domain |
|  |  | Pd01 | 2,70E+07 | 2,70E+07 | recessive | AX-586027373 | GO:0003684 | Molecular Function:damaged DNA binding | K10839 | UV excision repair protein RAD23 | IPR004806 | UV excision repair protein Rad23 |
|  |  | Pd01 | 2,70E+07 | 2,70E+07 | recessive | AX-586027373 | GO:0006289 | Biological Process:nucleotide-excision repair | K10839 | UV excision repair protein RAD23 | IPR029071 | Ubiquitin-like domain superfamily |
|  |  | Pd01 | 2,70E+07 | 2,70E+07 | recessive | AX-586027373 | GO:0006289 | Biological Process:nucleotide-excision repair | K10839 | UV excision repair protein RAD23 | IPR015940 | Ubiquitin-associated domain |
|  |  | Pd01 | 2,70E+07 | 2,70E+07 | recessive | AX-586027373 | GO:0006289 | Biological Process:nucleotide-excision repair | K10839 | UV excision repair protein RAD23 | IPR036353 | XPC-binding domain superfamily |
|  |  | Pd01 | 2,70E+07 | 2,70E+07 | recessive | AX-586027373 | GO:0006289 | Biological Process:nucleotide-excision repair | K10839 | UV excision repair protein RAD23 | IPR009060 | UBA-like superfamily |
|  |  | Pd01 | 2,70E+07 | 2,70E+07 | recessive | AX-586027373 | GO:0006289 | Biological Process:nucleotide-excision repair | K10839 | UV excision repair protein RAD23 | IPR000626 | Ubiquitin domain |
|  |  | Pd01 | 2,70E+07 | 2,70E+07 | recessive | AX-586027373 | GO:0006289 | Biological Process:nucleotide-excision repair | K10839 | UV excision repair protein RAD23 | IPR015360 | XPC-binding domain |
|  |  | Pd01 | 2,70E+07 | 2,70E+07 | recessive | AX-586027373 | GO:0006289 | Biological Process:nucleotide-excision repair | K10839 | UV excision repair protein RAD23 | IPR004806 | UV excision repair protein Rad23 |
| **Prudul26A024127T1** |  | Pd01 | 2,70E+07 | 2,70E+07 | recessive | AX-586027373 | NA | NA | NA | NA | NA | NA |
| **Prudul26A024985T1** |  | Pd01 | 2,70E+07 | 2,70E+07 | recessive | AX-586027373 | GO:0010112 | Biological Process:regulation of systemic acquired resistance | NA | NA | IPR034577 | Protein NIM1-INTERACTING 2 |
| **Prudul26A005756T1** | Upstream gene variant | Pd01 | 2,70E+07 | 2,70E+07 | recessive | AX-586027373 | GO:0004553 | Molecular Function:hydrolase activity, hydrolyzing O-glycosyl compounds | K15925 | alpha-D-xyloside xylohydrolase [EC:3.2.1.177] | IPR011013 | Galactose mutarotase-like domain superfamily |
|  |  | Pd01 | 2,70E+07 | 2,70E+07 | recessive | AX-586027373 | GO:0004553 | Molecular Function:hydrolase activity, hydrolyzing O-glycosyl compounds | K15925 | alpha-D-xyloside xylohydrolase [EC:3.2.1.177] | IPR030458 | Glycosyl hydrolases family 31, active site |
|  |  | Pd01 | 2,70E+07 | 2,70E+07 | recessive | AX-586027373 | GO:0004553 | Molecular Function:hydrolase activity, hydrolyzing O-glycosyl compounds | K15925 | alpha-D-xyloside xylohydrolase [EC:3.2.1.177] | IPR025887 | Glycoside hydrolase family 31, N-terminal domain |
|  |  | Pd01 | 2,70E+07 | 2,70E+07 | recessive | AX-586027373 | GO:0004553 | Molecular Function:hydrolase activity, hydrolyzing O-glycosyl compounds | K15925 | alpha-D-xyloside xylohydrolase [EC:3.2.1.177] | IPR013780 | Glycosyl hydrolase, all-beta |
|  |  | Pd01 | 2,70E+07 | 2,70E+07 | recessive | AX-586027373 | GO:0004553 | Molecular Function:hydrolase activity, hydrolyzing O-glycosyl compounds | K15925 | alpha-D-xyloside xylohydrolase [EC:3.2.1.177] | IPR031727 | Galactose mutarotase, N-terminal barrel |
|  |  | Pd01 | 2,70E+07 | 2,70E+07 | recessive | AX-586027373 | GO:0004553 | Molecular Function:hydrolase activity, hydrolyzing O-glycosyl compounds | K15925 | alpha-D-xyloside xylohydrolase [EC:3.2.1.177] | IPR000322 | Glycoside hydrolase family 31 |
|  |  | Pd01 | 2,70E+07 | 2,70E+07 | recessive | AX-586027373 | GO:0004553 | Molecular Function:hydrolase activity, hydrolyzing O-glycosyl compounds | K15925 | alpha-D-xyloside xylohydrolase [EC:3.2.1.177] | IPR017853 | Glycoside hydrolase superfamily |
|  |  | Pd01 | 2,70E+07 | 2,70E+07 | recessive | AX-586027373 | GO:0004553 | Molecular Function:hydrolase activity, hydrolyzing O-glycosyl compounds | K15925 | alpha-D-xyloside xylohydrolase [EC:3.2.1.177] | IPR030459 | Glycosyl hydrolases family 31, conserved site |
|  |  | Pd01 | 2,70E+07 | 2,70E+07 | recessive | AX-586027373 | GO:0005975 | Biological Process:carbohydrate metabolic process | K15925 | alpha-D-xyloside xylohydrolase [EC:3.2.1.177] | IPR011013 | Galactose mutarotase-like domain superfamily |
|  |  | Pd01 | 2,70E+07 | 2,70E+07 | recessive | AX-586027373 | GO:0005975 | Biological Process:carbohydrate metabolic process | K15925 | alpha-D-xyloside xylohydrolase [EC:3.2.1.177] | IPR030458 | Glycosyl hydrolases family 31, active site |
|  |  | Pd01 | 2,70E+07 | 2,70E+07 | recessive | AX-586027373 | GO:0005975 | Biological Process:carbohydrate metabolic process | K15925 | alpha-D-xyloside xylohydrolase [EC:3.2.1.177] | IPR025887 | Glycoside hydrolase family 31, N-terminal domain |
|  |  | Pd01 | 2,70E+07 | 2,70E+07 | recessive | AX-586027373 | GO:0005975 | Biological Process:carbohydrate metabolic process | K15925 | alpha-D-xyloside xylohydrolase [EC:3.2.1.177] | IPR013780 | Glycosyl hydrolase, all-beta |
|  |  | Pd01 | 2,70E+07 | 2,70E+07 | recessive | AX-586027373 | GO:0005975 | Biological Process:carbohydrate metabolic process | K15925 | alpha-D-xyloside xylohydrolase [EC:3.2.1.177] | IPR031727 | Galactose mutarotase, N-terminal barrel |
|  |  | Pd01 | 2,70E+07 | 2,70E+07 | recessive | AX-586027373 | GO:0005975 | Biological Process:carbohydrate metabolic process | K15925 | alpha-D-xyloside xylohydrolase [EC:3.2.1.177] | IPR000322 | Glycoside hydrolase family 31 |
|  |  | Pd01 | 2,70E+07 | 2,70E+07 | recessive | AX-586027373 | GO:0005975 | Biological Process:carbohydrate metabolic process | K15925 | alpha-D-xyloside xylohydrolase [EC:3.2.1.177] | IPR017853 | Glycoside hydrolase superfamily |
|  |  | Pd01 | 2,70E+07 | 2,70E+07 | recessive | AX-586027373 | GO:0005975 | Biological Process:carbohydrate metabolic process | K15925 | alpha-D-xyloside xylohydrolase [EC:3.2.1.177] | IPR030459 | Glycosyl hydrolases family 31, conserved site |
|  |  | Pd01 | 2,70E+07 | 2,70E+07 | recessive | AX-586027373 | GO:0030246 | Molecular Function:carbohydrate binding | K15925 | alpha-D-xyloside xylohydrolase [EC:3.2.1.177] | IPR011013 | Galactose mutarotase-like domain superfamily |
|  |  | Pd01 | 2,70E+07 | 2,70E+07 | recessive | AX-586027373 | GO:0030246 | Molecular Function:carbohydrate binding | K15925 | alpha-D-xyloside xylohydrolase [EC:3.2.1.177] | IPR030458 | Glycosyl hydrolases family 31, active site |
|  |  | Pd01 | 2,70E+07 | 2,70E+07 | recessive | AX-586027373 | GO:0030246 | Molecular Function:carbohydrate binding | K15925 | alpha-D-xyloside xylohydrolase [EC:3.2.1.177] | IPR025887 | Glycoside hydrolase family 31, N-terminal domain |
|  |  | Pd01 | 2,70E+07 | 2,70E+07 | recessive | AX-586027373 | GO:0030246 | Molecular Function:carbohydrate binding | K15925 | alpha-D-xyloside xylohydrolase [EC:3.2.1.177] | IPR013780 | Glycosyl hydrolase, all-beta |
|  |  | Pd01 | 2,70E+07 | 2,70E+07 | recessive | AX-586027373 | GO:0030246 | Molecular Function:carbohydrate binding | K15925 | alpha-D-xyloside xylohydrolase [EC:3.2.1.177] | IPR031727 | Galactose mutarotase, N-terminal barrel |
|  |  | Pd01 | 2,70E+07 | 2,70E+07 | recessive | AX-586027373 | GO:0030246 | Molecular Function:carbohydrate binding | K15925 | alpha-D-xyloside xylohydrolase [EC:3.2.1.177] | IPR000322 | Glycoside hydrolase family 31 |
|  |  | Pd01 | 2,70E+07 | 2,70E+07 | recessive | AX-586027373 | GO:0030246 | Molecular Function:carbohydrate binding | K15925 | alpha-D-xyloside xylohydrolase [EC:3.2.1.177] | IPR017853 | Glycoside hydrolase superfamily |
|  |  | Pd01 | 2,70E+07 | 2,70E+07 | recessive | AX-586027373 | GO:0030246 | Molecular Function:carbohydrate binding | K15925 | alpha-D-xyloside xylohydrolase [EC:3.2.1.177] | IPR030459 | Glycosyl hydrolases family 31, conserved site |
|  |  | Pd01 | 2,70E+07 | 2,70E+07 | recessive | AX-586027373 | GO:0003824 | Molecular Function:catalytic activity | K15925 | alpha-D-xyloside xylohydrolase [EC:3.2.1.177] | IPR011013 | Galactose mutarotase-like domain superfamily |
|  |  | Pd01 | 2,70E+07 | 2,70E+07 | recessive | AX-586027373 | GO:0003824 | Molecular Function:catalytic activity | K15925 | alpha-D-xyloside xylohydrolase [EC:3.2.1.177] | IPR030458 | Glycosyl hydrolases family 31, active site |
|  |  | Pd01 | 2,70E+07 | 2,70E+07 | recessive | AX-586027373 | GO:0003824 | Molecular Function:catalytic activity | K15925 | alpha-D-xyloside xylohydrolase [EC:3.2.1.177] | IPR025887 | Glycoside hydrolase family 31, N-terminal domain |
|  |  | Pd01 | 2,70E+07 | 2,70E+07 | recessive | AX-586027373 | GO:0003824 | Molecular Function:catalytic activity | K15925 | alpha-D-xyloside xylohydrolase [EC:3.2.1.177] | IPR013780 | Glycosyl hydrolase, all-beta |
|  |  | Pd01 | 2,70E+07 | 2,70E+07 | recessive | AX-586027373 | GO:0003824 | Molecular Function:catalytic activity | K15925 | alpha-D-xyloside xylohydrolase [EC:3.2.1.177] | IPR031727 | Galactose mutarotase, N-terminal barrel |
|  |  | Pd01 | 2,70E+07 | 2,70E+07 | recessive | AX-586027373 | GO:0003824 | Molecular Function:catalytic activity | K15925 | alpha-D-xyloside xylohydrolase [EC:3.2.1.177] | IPR000322 | Glycoside hydrolase family 31 |
|  |  | Pd01 | 2,70E+07 | 2,70E+07 | recessive | AX-586027373 | GO:0003824 | Molecular Function:catalytic activity | K15925 | alpha-D-xyloside xylohydrolase [EC:3.2.1.177] | IPR017853 | Glycoside hydrolase superfamily |
|  |  | Pd01 | 2,70E+07 | 2,70E+07 | recessive | AX-586027373 | GO:0003824 | Molecular Function:catalytic activity | K15925 | alpha-D-xyloside xylohydrolase [EC:3.2.1.177] | IPR030459 | Glycosyl hydrolases family 31, conserved site |
| **Prudul26A013883T1** |  | Pd01 | 2,70E+07 | 2,70E+07 | recessive | AX-586027373 | NA | NA | NA | NA | NA | NA |
| **Prudul26A012219T1** |  | Pd01 | 2,70E+07 | 2,70E+07 | recessive | AX-586027373 | GO:0003700 | Molecular Function:DNA-binding transcription factor activity | NA | NA | IPR036955 | AP2/ERF domain superfamily |
|  |  | Pd01 | 2,70E+07 | 2,70E+07 | recessive | AX-586027373 | GO:0003700 | Molecular Function:DNA-binding transcription factor activity | NA | NA | IPR001471 | AP2/ERF domain |
|  |  | Pd01 | 2,70E+07 | 2,70E+07 | recessive | AX-586027373 | GO:0003700 | Molecular Function:DNA-binding transcription factor activity | NA | NA | IPR016177 | DNA-binding domain superfamily |
|  |  | Pd01 | 2,70E+07 | 2,70E+07 | recessive | AX-586027373 | GO:0003677 | Molecular Function:DNA binding | NA | NA | IPR036955 | AP2/ERF domain superfamily |
|  |  | Pd01 | 2,70E+07 | 2,70E+07 | recessive | AX-586027373 | GO:0003677 | Molecular Function:DNA binding | NA | NA | IPR001471 | AP2/ERF domain |
|  |  | Pd01 | 2,70E+07 | 2,70E+07 | recessive | AX-586027373 | GO:0003677 | Molecular Function:DNA binding | NA | NA | IPR016177 | DNA-binding domain superfamily |
|  |  | Pd01 | 2,70E+07 | 2,70E+07 | recessive | AX-586027373 | GO:0006355 | Biological Process:regulation of transcription, DNA-templated | NA | NA | IPR036955 | AP2/ERF domain superfamily |
|  |  | Pd01 | 2,70E+07 | 2,70E+07 | recessive | AX-586027373 | GO:0006355 | Biological Process:regulation of transcription, DNA-templated | NA | NA | IPR001471 | AP2/ERF domain |
|  |  | Pd01 | 2,70E+07 | 2,70E+07 | recessive | AX-586027373 | GO:0006355 | Biological Process:regulation of transcription, DNA-templated | NA | NA | IPR016177 | DNA-binding domain superfamily |
| **Prudul26A029564T1** |  | Pd01 | 2,70E+07 | 2,70E+07 | recessive | AX-586027373 | GO:0050662 | Molecular Function:coenzyme binding | NA | NA | IPR001509 | NAD-dependent epimerase/dehydratase |
|  |  | Pd01 | 2,70E+07 | 2,70E+07 | recessive | AX-586027373 | GO:0050662 | Molecular Function:coenzyme binding | NA | NA | IPR036291 | NAD(P)-binding domain superfamily |
|  |  | Pd01 | 2,70E+07 | 2,70E+07 | recessive | AX-586027373 | GO:0003824 | Molecular Function:catalytic activity | NA | NA | IPR001509 | NAD-dependent epimerase/dehydratase |
|  |  | Pd01 | 2,70E+07 | 2,70E+07 | recessive | AX-586027373 | GO:0003824 | Molecular Function:catalytic activity | NA | NA | IPR036291 | NAD(P)-binding domain superfamily |
| **Prudul26A027048T1** |  | Pd01 | 2,70E+07 | 2,70E+07 | recessive | AX-586027373 | GO:0006633 | Biological Process:fatty acid biosynthetic process | K15397 | 3-ketoacyl-CoA synthase [EC:2.3.1.199] | IPR016039 | Thiolase-like |
|  |  | Pd01 | 2,70E+07 | 2,70E+07 | recessive | AX-586027373 | GO:0006633 | Biological Process:fatty acid biosynthetic process | K15397 | 3-ketoacyl-CoA synthase [EC:2.3.1.199] | IPR013747 | 3-Oxoacyl-[acyl-carrier-protein (ACP)] synthase III, C-terminal |
|  |  | Pd01 | 2,70E+07 | 2,70E+07 | recessive | AX-586027373 | GO:0006633 | Biological Process:fatty acid biosynthetic process | K15397 | 3-ketoacyl-CoA synthase [EC:2.3.1.199] | IPR013601 | FAE1/Type III polyketide synthase-like protein |
|  |  | Pd01 | 2,70E+07 | 2,70E+07 | recessive | AX-586027373 | GO:0006633 | Biological Process:fatty acid biosynthetic process | K15397 | 3-ketoacyl-CoA synthase [EC:2.3.1.199] | IPR012392 | Very-long-chain 3-ketoacyl-CoA synthase |
|  |  | Pd01 | 2,70E+07 | 2,70E+07 | recessive | AX-586027373 | GO:0003824 | Molecular Function:catalytic activity | K15397 | 3-ketoacyl-CoA synthase [EC:2.3.1.199] | IPR016039 | Thiolase-like |
|  |  | Pd01 | 2,70E+07 | 2,70E+07 | recessive | AX-586027373 | GO:0003824 | Molecular Function:catalytic activity | K15397 | 3-ketoacyl-CoA synthase [EC:2.3.1.199] | IPR013747 | 3-Oxoacyl-[acyl-carrier-protein (ACP)] synthase III, C-terminal |
|  |  | Pd01 | 2,70E+07 | 2,70E+07 | recessive | AX-586027373 | GO:0003824 | Molecular Function:catalytic activity | K15397 | 3-ketoacyl-CoA synthase [EC:2.3.1.199] | IPR013601 | FAE1/Type III polyketide synthase-like protein |
|  |  | Pd01 | 2,70E+07 | 2,70E+07 | recessive | AX-586027373 | GO:0003824 | Molecular Function:catalytic activity | K15397 | 3-ketoacyl-CoA synthase [EC:2.3.1.199] | IPR012392 | Very-long-chain 3-ketoacyl-CoA synthase |
|  |  | Pd01 | 2,70E+07 | 2,70E+07 | recessive | AX-586027373 | GO:0016747 | Molecular Function:transferase activity, transferring acyl groups other than amino-acyl groups | K15397 | 3-ketoacyl-CoA synthase [EC:2.3.1.199] | IPR016039 | Thiolase-like |
|  |  | Pd01 | 2,70E+07 | 2,70E+07 | recessive | AX-586027373 | GO:0016747 | Molecular Function:transferase activity, transferring acyl groups other than amino-acyl groups | K15397 | 3-ketoacyl-CoA synthase [EC:2.3.1.199] | IPR013747 | 3-Oxoacyl-[acyl-carrier-protein (ACP)] synthase III, C-terminal |
|  |  | Pd01 | 2,70E+07 | 2,70E+07 | recessive | AX-586027373 | GO:0016747 | Molecular Function:transferase activity, transferring acyl groups other than amino-acyl groups | K15397 | 3-ketoacyl-CoA synthase [EC:2.3.1.199] | IPR013601 | FAE1/Type III polyketide synthase-like protein |
|  |  | Pd01 | 2,70E+07 | 2,70E+07 | recessive | AX-586027373 | GO:0016747 | Molecular Function:transferase activity, transferring acyl groups other than amino-acyl groups | K15397 | 3-ketoacyl-CoA synthase [EC:2.3.1.199] | IPR012392 | Very-long-chain 3-ketoacyl-CoA synthase |
|  |  | Pd01 | 2,70E+07 | 2,70E+07 | recessive | AX-586027373 | GO:0016020 | Cellular Component:membrane | K15397 | 3-ketoacyl-CoA synthase [EC:2.3.1.199] | IPR016039 | Thiolase-like |
|  |  | Pd01 | 2,70E+07 | 2,70E+07 | recessive | AX-586027373 | GO:0016020 | Cellular Component:membrane | K15397 | 3-ketoacyl-CoA synthase [EC:2.3.1.199] | IPR013747 | 3-Oxoacyl-[acyl-carrier-protein (ACP)] synthase III, C-terminal |
|  |  | Pd01 | 2,70E+07 | 2,70E+07 | recessive | AX-586027373 | GO:0016020 | Cellular Component:membrane | K15397 | 3-ketoacyl-CoA synthase [EC:2.3.1.199] | IPR013601 | FAE1/Type III polyketide synthase-like protein |
|  |  | Pd01 | 2,70E+07 | 2,70E+07 | recessive | AX-586027373 | GO:0016020 | Cellular Component:membrane | K15397 | 3-ketoacyl-CoA synthase [EC:2.3.1.199] | IPR012392 | Very-long-chain 3-ketoacyl-CoA synthase |
| **Prudul26A027048T2** |  | Pd01 | 2,70E+07 | 2,70E+07 | recessive | AX-586027373 | GO:0016020 | Cellular Component:membrane | K15397 | 3-ketoacyl-CoA synthase [EC:2.3.1.199] | IPR013601 | FAE1/Type III polyketide synthase-like protein |
|  |  | Pd01 | 2,70E+07 | 2,70E+07 | recessive | AX-586027373 | GO:0016020 | Cellular Component:membrane | K15397 | 3-ketoacyl-CoA synthase [EC:2.3.1.199] | IPR013747 | 3-Oxoacyl-[acyl-carrier-protein (ACP)] synthase III, C-terminal |
|  |  | Pd01 | 2,70E+07 | 2,70E+07 | recessive | AX-586027373 | GO:0016020 | Cellular Component:membrane | K15397 | 3-ketoacyl-CoA synthase [EC:2.3.1.199] | IPR012392 | Very-long-chain 3-ketoacyl-CoA synthase |
|  |  | Pd01 | 2,70E+07 | 2,70E+07 | recessive | AX-586027373 | GO:0016020 | Cellular Component:membrane | K15397 | 3-ketoacyl-CoA synthase [EC:2.3.1.199] | IPR016039 | Thiolase-like |
|  |  | Pd01 | 2,70E+07 | 2,70E+07 | recessive | AX-586027373 | GO:0006633 | Biological Process:fatty acid biosynthetic process | K15397 | 3-ketoacyl-CoA synthase [EC:2.3.1.199] | IPR013601 | FAE1/Type III polyketide synthase-like protein |
|  |  | Pd01 | 2,70E+07 | 2,70E+07 | recessive | AX-586027373 | GO:0006633 | Biological Process:fatty acid biosynthetic process | K15397 | 3-ketoacyl-CoA synthase [EC:2.3.1.199] | IPR013747 | 3-Oxoacyl-[acyl-carrier-protein (ACP)] synthase III, C-terminal |
|  |  | Pd01 | 2,70E+07 | 2,70E+07 | recessive | AX-586027373 | GO:0006633 | Biological Process:fatty acid biosynthetic process | K15397 | 3-ketoacyl-CoA synthase [EC:2.3.1.199] | IPR012392 | Very-long-chain 3-ketoacyl-CoA synthase |
|  |  | Pd01 | 2,70E+07 | 2,70E+07 | recessive | AX-586027373 | GO:0006633 | Biological Process:fatty acid biosynthetic process | K15397 | 3-ketoacyl-CoA synthase [EC:2.3.1.199] | IPR016039 | Thiolase-like |
|  |  | Pd01 | 2,70E+07 | 2,70E+07 | recessive | AX-586027373 | GO:0003824 | Molecular Function:catalytic activity | K15397 | 3-ketoacyl-CoA synthase [EC:2.3.1.199] | IPR013601 | FAE1/Type III polyketide synthase-like protein |
|  |  | Pd01 | 2,70E+07 | 2,70E+07 | recessive | AX-586027373 | GO:0003824 | Molecular Function:catalytic activity | K15397 | 3-ketoacyl-CoA synthase [EC:2.3.1.199] | IPR013747 | 3-Oxoacyl-[acyl-carrier-protein (ACP)] synthase III, C-terminal |
|  |  | Pd01 | 2,70E+07 | 2,70E+07 | recessive | AX-586027373 | GO:0003824 | Molecular Function:catalytic activity | K15397 | 3-ketoacyl-CoA synthase [EC:2.3.1.199] | IPR012392 | Very-long-chain 3-ketoacyl-CoA synthase |
|  |  | Pd01 | 2,70E+07 | 2,70E+07 | recessive | AX-586027373 | GO:0003824 | Molecular Function:catalytic activity | K15397 | 3-ketoacyl-CoA synthase [EC:2.3.1.199] | IPR016039 | Thiolase-like |
|  |  | Pd01 | 2,70E+07 | 2,70E+07 | recessive | AX-586027373 | GO:0016747 | Molecular Function:transferase activity, transferring acyl groups other than amino-acyl groups | K15397 | 3-ketoacyl-CoA synthase [EC:2.3.1.199] | IPR013601 | FAE1/Type III polyketide synthase-like protein |
|  |  | Pd01 | 2,70E+07 | 2,70E+07 | recessive | AX-586027373 | GO:0016747 | Molecular Function:transferase activity, transferring acyl groups other than amino-acyl groups | K15397 | 3-ketoacyl-CoA synthase [EC:2.3.1.199] | IPR013747 | 3-Oxoacyl-[acyl-carrier-protein (ACP)] synthase III, C-terminal |
|  |  | Pd01 | 2,70E+07 | 2,70E+07 | recessive | AX-586027373 | GO:0016747 | Molecular Function:transferase activity, transferring acyl groups other than amino-acyl groups | K15397 | 3-ketoacyl-CoA synthase [EC:2.3.1.199] | IPR012392 | Very-long-chain 3-ketoacyl-CoA synthase |
|  |  | Pd01 | 2,70E+07 | 2,70E+07 | recessive | AX-586027373 | GO:0016747 | Molecular Function:transferase activity, transferring acyl groups other than amino-acyl groups | K15397 | 3-ketoacyl-CoA synthase [EC:2.3.1.199] | IPR016039 | Thiolase-like |
| **Prudul26A004166T1** |  | Pd01 | 2,70E+07 | 2,70E+07 | recessive | AX-586027373 | GO:0047150 | Molecular Function:betaine-homocysteine S-methyltransferase activity | K00547 | homocysteine S-methyltransferase [EC:2.1.1.10] | IPR036589 | Homocysteine-binding domain superfamily |
|  |  | Pd01 | 2,70E+07 | 2,70E+07 | recessive | AX-586027373 | GO:0047150 | Molecular Function:betaine-homocysteine S-methyltransferase activity | K00547 | homocysteine S-methyltransferase [EC:2.1.1.10] | IPR017226 | Betaine-homocysteine S-methyltransferase, BHMT |
|  |  | Pd01 | 2,70E+07 | 2,70E+07 | recessive | AX-586027373 | GO:0047150 | Molecular Function:betaine-homocysteine S-methyltransferase activity | K00547 | homocysteine S-methyltransferase [EC:2.1.1.10] | IPR003726 | Homocysteine-binding domain |
|  |  | Pd01 | 2,70E+07 | 2,70E+07 | recessive | AX-586027373 | GO:0009086 | Biological Process:methionine biosynthetic process | K00547 | homocysteine S-methyltransferase [EC:2.1.1.10] | IPR036589 | Homocysteine-binding domain superfamily |
|  |  | Pd01 | 2,70E+07 | 2,70E+07 | recessive | AX-586027373 | GO:0009086 | Biological Process:methionine biosynthetic process | K00547 | homocysteine S-methyltransferase [EC:2.1.1.10] | IPR017226 | Betaine-homocysteine S-methyltransferase, BHMT |
|  |  | Pd01 | 2,70E+07 | 2,70E+07 | recessive | AX-586027373 | GO:0009086 | Biological Process:methionine biosynthetic process | K00547 | homocysteine S-methyltransferase [EC:2.1.1.10] | IPR003726 | Homocysteine-binding domain |
|  |  | Pd01 | 2,70E+07 | 2,70E+07 | recessive | AX-586027373 | GO:0008270 | Molecular Function:zinc ion binding | K00547 | homocysteine S-methyltransferase [EC:2.1.1.10] | IPR036589 | Homocysteine-binding domain superfamily |
|  |  | Pd01 | 2,70E+07 | 2,70E+07 | recessive | AX-586027373 | GO:0008270 | Molecular Function:zinc ion binding | K00547 | homocysteine S-methyltransferase [EC:2.1.1.10] | IPR017226 | Betaine-homocysteine S-methyltransferase, BHMT |
|  |  | Pd01 | 2,70E+07 | 2,70E+07 | recessive | AX-586027373 | GO:0008270 | Molecular Function:zinc ion binding | K00547 | homocysteine S-methyltransferase [EC:2.1.1.10] | IPR003726 | Homocysteine-binding domain |
|  |  | Pd01 | 2,70E+07 | 2,70E+07 | recessive | AX-586027373 | GO:0005737 | Cellular Component:cytoplasm | K00547 | homocysteine S-methyltransferase [EC:2.1.1.10] | IPR036589 | Homocysteine-binding domain superfamily |
|  |  | Pd01 | 2,70E+07 | 2,70E+07 | recessive | AX-586027373 | GO:0005737 | Cellular Component:cytoplasm | K00547 | homocysteine S-methyltransferase [EC:2.1.1.10] | IPR017226 | Betaine-homocysteine S-methyltransferase, BHMT |
|  |  | Pd01 | 2,70E+07 | 2,70E+07 | recessive | AX-586027373 | GO:0005737 | Cellular Component:cytoplasm | K00547 | homocysteine S-methyltransferase [EC:2.1.1.10] | IPR003726 | Homocysteine-binding domain |
| **Prudul26A008703T1** |  | Pd01 | 2,70E+07 | 2,70E+07 | recessive | AX-586027373 | NA | NA | NA | NA | NA | NA |
| **Prudul26A024462T1** |  | Pd01 | 2,70E+07 | 2,70E+07 | recessive | AX-586027373 | GO:0008270 | Molecular Function:zinc ion binding | NA | NA | IPR010402 | CCT domain |
|  |  | Pd01 | 2,70E+07 | 2,70E+07 | recessive | AX-586027373 | GO:0008270 | Molecular Function:zinc ion binding | NA | NA | IPR000315 | B-box-type zinc finger |
|  |  | Pd01 | 2,70E+07 | 2,70E+07 | recessive | AX-586027373 | GO:0005622 | Cellular Component:intracellular | NA | NA | IPR010402 | CCT domain |
|  |  | Pd01 | 2,70E+07 | 2,70E+07 | recessive | AX-586027373 | GO:0005622 | Cellular Component:intracellular | NA | NA | IPR000315 | B-box-type zinc finger |
|  |  | Pd01 | 2,70E+07 | 2,70E+07 | recessive | AX-586027373 | GO:0005515 | Molecular Function:protein binding | NA | NA | IPR010402 | CCT domain |
|  |  | Pd01 | 2,70E+07 | 2,70E+07 | recessive | AX-586027373 | GO:0005515 | Molecular Function:protein binding | NA | NA | IPR000315 | B-box-type zinc finger |
| **Prudul26A031606T1** |  | Pd01 | 2,70E+07 | 2,70E+07 | recessive | AX-586027373 | NA | NA | NA | NA | IPR004883 | Lateral organ boundaries, LOB |
| **Prudul26A022111T1** |  | Pd02 | 1,40E+07 | 1,40E+07 | recessive | AX-586048050 | NA | NA | NA | NA | NA | NA |
| **Prudul26A029199T1** |  | Pd02 | 1,40E+07 | 1,40E+07 | recessive | AX-586048050 | GO:0043531 | Molecular Function:ADP binding | NA | NA | IPR032675 | Leucine-rich repeat domain superfamily |
|  |  | Pd02 | 1,40E+07 | 1,40E+07 | recessive | AX-586048050 | GO:0043531 | Molecular Function:ADP binding | NA | NA | IPR000157 | Toll/interleukin-1 receptor homology (TIR) domain |
|  |  | Pd02 | 1,40E+07 | 1,40E+07 | recessive | AX-586048050 | GO:0043531 | Molecular Function:ADP binding | NA | NA | IPR002182 | NB-ARC |
|  |  | Pd02 | 1,40E+07 | 1,40E+07 | recessive | AX-586048050 | GO:0043531 | Molecular Function:ADP binding | NA | NA | IPR035897 | Toll/interleukin-1 receptor homology (TIR) domain superfamily |
|  |  | Pd02 | 1,40E+07 | 1,40E+07 | recessive | AX-586048050 | GO:0043531 | Molecular Function:ADP binding | NA | NA | IPR027417 | P-loop containing nucleoside triphosphate hydrolase |
|  |  | Pd02 | 1,40E+07 | 1,40E+07 | recessive | AX-586048050 | GO:0007165 | Biological Process:signal transduction | NA | NA | IPR032675 | Leucine-rich repeat domain superfamily |
|  |  | Pd02 | 1,40E+07 | 1,40E+07 | recessive | AX-586048050 | GO:0007165 | Biological Process:signal transduction | NA | NA | IPR000157 | Toll/interleukin-1 receptor homology (TIR) domain |
|  |  | Pd02 | 1,40E+07 | 1,40E+07 | recessive | AX-586048050 | GO:0007165 | Biological Process:signal transduction | NA | NA | IPR002182 | NB-ARC |
|  |  | Pd02 | 1,40E+07 | 1,40E+07 | recessive | AX-586048050 | GO:0007165 | Biological Process:signal transduction | NA | NA | IPR035897 | Toll/interleukin-1 receptor homology (TIR) domain superfamily |
|  |  | Pd02 | 1,40E+07 | 1,40E+07 | recessive | AX-586048050 | GO:0007165 | Biological Process:signal transduction | NA | NA | IPR027417 | P-loop containing nucleoside triphosphate hydrolase |
|  |  | Pd02 | 1,40E+07 | 1,40E+07 | recessive | AX-586048050 | GO:0005515 | Molecular Function:protein binding | NA | NA | IPR032675 | Leucine-rich repeat domain superfamily |
|  |  | Pd02 | 1,40E+07 | 1,40E+07 | recessive | AX-586048050 | GO:0005515 | Molecular Function:protein binding | NA | NA | IPR000157 | Toll/interleukin-1 receptor homology (TIR) domain |
|  |  | Pd02 | 1,40E+07 | 1,40E+07 | recessive | AX-586048050 | GO:0005515 | Molecular Function:protein binding | NA | NA | IPR002182 | NB-ARC |
|  |  | Pd02 | 1,40E+07 | 1,40E+07 | recessive | AX-586048050 | GO:0005515 | Molecular Function:protein binding | NA | NA | IPR035897 | Toll/interleukin-1 receptor homology (TIR) domain superfamily |
|  |  | Pd02 | 1,40E+07 | 1,40E+07 | recessive | AX-586048050 | GO:0005515 | Molecular Function:protein binding | NA | NA | IPR027417 | P-loop containing nucleoside triphosphate hydrolase |
| **Prudul26A011151T1** |  | Pd02 | 1,40E+07 | 1,40E+07 | recessive | AX-586048050 | GO:0005634 | Cellular Component:nucleus | NA | NA | IPR012617 | Apoptosis-antagonizing transcription factor, C-terminal |
|  |  | Pd02 | 1,40E+07 | 1,40E+07 | recessive | AX-586048050 | GO:0005634 | Cellular Component:nucleus | NA | NA | IPR025160 | AATF leucine zipper-containing domain |
|  |  | Pd02 | 1,40E+07 | 1,40E+07 | recessive | AX-586048050 | GO:0005634 | Cellular Component:nucleus | NA | NA | IPR039223 | Protein AATF/Bfr2 |
| **Prudul26A011151T2** |  | Pd02 | 1,40E+07 | 1,40E+07 | recessive | AX-586048050 | GO:0005634 | Cellular Component:nucleus | NA | NA | IPR025160 | AATF leucine zipper-containing domain |
|  |  | Pd02 | 1,40E+07 | 1,40E+07 | recessive | AX-586048050 | GO:0005634 | Cellular Component:nucleus | NA | NA | IPR012617 | Apoptosis-antagonizing transcription factor, C-terminal |
|  |  | Pd02 | 1,40E+07 | 1,40E+07 | recessive | AX-586048050 | GO:0005634 | Cellular Component:nucleus | NA | NA | IPR039223 | Protein AATF/Bfr2 |
| **Prudul26A011151T3** |  | Pd02 | 1,40E+07 | 1,40E+07 | recessive | AX-586048050 | GO:0005634 | Cellular Component:nucleus | NA | NA | IPR025160 | AATF leucine zipper-containing domain |
|  |  | Pd02 | 1,40E+07 | 1,40E+07 | recessive | AX-586048050 | GO:0005634 | Cellular Component:nucleus | NA | NA | IPR039223 | Protein AATF/Bfr2 |
|  |  | Pd02 | 1,40E+07 | 1,40E+07 | recessive | AX-586048050 | GO:0005634 | Cellular Component:nucleus | NA | NA | IPR012617 | Apoptosis-antagonizing transcription factor, C-terminal |
| **Prudul26A011151T4** |  | Pd02 | 1,40E+07 | 1,40E+07 | recessive | AX-586048050 | GO:0005634 | Cellular Component:nucleus | NA | NA | IPR039223 | Protein AATF/Bfr2 |
|  |  | Pd02 | 1,40E+07 | 1,40E+07 | recessive | AX-586048050 | GO:0005634 | Cellular Component:nucleus | NA | NA | IPR012617 | Apoptosis-antagonizing transcription factor, C-terminal |
|  |  | Pd02 | 1,40E+07 | 1,40E+07 | recessive | AX-586048050 | GO:0005634 | Cellular Component:nucleus | NA | NA | IPR025160 | AATF leucine zipper-containing domain |
| **Prudul26A018199T1** | Downstream gene variant | Pd02 | 1,40E+07 | 1,40E+07 | recessive | AX-586048050 | GO:0003677 | Molecular Function:DNA binding | K09338 | homeobox-leucine zipper protein | IPR003106 | Leucine zipper, homeobox-associated |
|  |  | Pd02 | 1,40E+07 | 1,40E+07 | recessive | AX-586048050 | GO:0003677 | Molecular Function:DNA binding | K09338 | homeobox-leucine zipper protein | IPR006712 | HD-ZIP protein, N-terminal |
|  |  | Pd02 | 1,40E+07 | 1,40E+07 | recessive | AX-586048050 | GO:0003677 | Molecular Function:DNA binding | K09338 | homeobox-leucine zipper protein | IPR001356 | Homeobox domain |
|  |  | Pd02 | 1,40E+07 | 1,40E+07 | recessive | AX-586048050 | GO:0003677 | Molecular Function:DNA binding | K09338 | homeobox-leucine zipper protein | IPR017970 | Homeobox, conserved site |
|  |  | Pd02 | 1,40E+07 | 1,40E+07 | recessive | AX-586048050 | GO:0003677 | Molecular Function:DNA binding | K09338 | homeobox-leucine zipper protein | IPR009057 | Homeobox-like domain superfamily |
|  |  | Pd02 | 1,40E+07 | 1,40E+07 | recessive | AX-586048050 | GO:0006355 | Biological Process:regulation of transcription, DNA-templated | K09338 | homeobox-leucine zipper protein | IPR003106 | Leucine zipper, homeobox-associated |
|  |  | Pd02 | 1,40E+07 | 1,40E+07 | recessive | AX-586048050 | GO:0006355 | Biological Process:regulation of transcription, DNA-templated | K09338 | homeobox-leucine zipper protein | IPR006712 | HD-ZIP protein, N-terminal |
|  |  | Pd02 | 1,40E+07 | 1,40E+07 | recessive | AX-586048050 | GO:0006355 | Biological Process:regulation of transcription, DNA-templated | K09338 | homeobox-leucine zipper protein | IPR001356 | Homeobox domain |
|  |  | Pd02 | 1,40E+07 | 1,40E+07 | recessive | AX-586048050 | GO:0006355 | Biological Process:regulation of transcription, DNA-templated | K09338 | homeobox-leucine zipper protein | IPR017970 | Homeobox, conserved site |
|  |  | Pd02 | 1,40E+07 | 1,40E+07 | recessive | AX-586048050 | GO:0006355 | Biological Process:regulation of transcription, DNA-templated | K09338 | homeobox-leucine zipper protein | IPR009057 | Homeobox-like domain superfamily |
|  |  | Pd02 | 1,40E+07 | 1,40E+07 | recessive | AX-586048050 | GO:0043565 | Molecular Function:sequence-specific DNA binding | K09338 | homeobox-leucine zipper protein | IPR003106 | Leucine zipper, homeobox-associated |
|  |  | Pd02 | 1,40E+07 | 1,40E+07 | recessive | AX-586048050 | GO:0043565 | Molecular Function:sequence-specific DNA binding | K09338 | homeobox-leucine zipper protein | IPR006712 | HD-ZIP protein, N-terminal |
|  |  | Pd02 | 1,40E+07 | 1,40E+07 | recessive | AX-586048050 | GO:0043565 | Molecular Function:sequence-specific DNA binding | K09338 | homeobox-leucine zipper protein | IPR001356 | Homeobox domain |
|  |  | Pd02 | 1,40E+07 | 1,40E+07 | recessive | AX-586048050 | GO:0043565 | Molecular Function:sequence-specific DNA binding | K09338 | homeobox-leucine zipper protein | IPR017970 | Homeobox, conserved site |
|  |  | Pd02 | 1,40E+07 | 1,40E+07 | recessive | AX-586048050 | GO:0043565 | Molecular Function:sequence-specific DNA binding | K09338 | homeobox-leucine zipper protein | IPR009057 | Homeobox-like domain superfamily |
|  |  | Pd02 | 1,40E+07 | 1,40E+07 | recessive | AX-586048050 | GO:0003700 | Molecular Function:DNA-binding transcription factor activity | K09338 | homeobox-leucine zipper protein | IPR003106 | Leucine zipper, homeobox-associated |
|  |  | Pd02 | 1,40E+07 | 1,40E+07 | recessive | AX-586048050 | GO:0003700 | Molecular Function:DNA-binding transcription factor activity | K09338 | homeobox-leucine zipper protein | IPR006712 | HD-ZIP protein, N-terminal |
|  |  | Pd02 | 1,40E+07 | 1,40E+07 | recessive | AX-586048050 | GO:0003700 | Molecular Function:DNA-binding transcription factor activity | K09338 | homeobox-leucine zipper protein | IPR001356 | Homeobox domain |
|  |  | Pd02 | 1,40E+07 | 1,40E+07 | recessive | AX-586048050 | GO:0003700 | Molecular Function:DNA-binding transcription factor activity | K09338 | homeobox-leucine zipper protein | IPR017970 | Homeobox, conserved site |
|  |  | Pd02 | 1,40E+07 | 1,40E+07 | recessive | AX-586048050 | GO:0003700 | Molecular Function:DNA-binding transcription factor activity | K09338 | homeobox-leucine zipper protein | IPR009057 | Homeobox-like domain superfamily |
|  |  | Pd02 | 1,40E+07 | 1,40E+07 | recessive | AX-586048050 | GO:0005634 | Cellular Component:nucleus | K09338 | homeobox-leucine zipper protein | IPR003106 | Leucine zipper, homeobox-associated |
|  |  | Pd02 | 1,40E+07 | 1,40E+07 | recessive | AX-586048050 | GO:0005634 | Cellular Component:nucleus | K09338 | homeobox-leucine zipper protein | IPR006712 | HD-ZIP protein, N-terminal |
|  |  | Pd02 | 1,40E+07 | 1,40E+07 | recessive | AX-586048050 | GO:0005634 | Cellular Component:nucleus | K09338 | homeobox-leucine zipper protein | IPR001356 | Homeobox domain |
|  |  | Pd02 | 1,40E+07 | 1,40E+07 | recessive | AX-586048050 | GO:0005634 | Cellular Component:nucleus | K09338 | homeobox-leucine zipper protein | IPR017970 | Homeobox, conserved site |
|  |  | Pd02 | 1,40E+07 | 1,40E+07 | recessive | AX-586048050 | GO:0005634 | Cellular Component:nucleus | K09338 | homeobox-leucine zipper protein | IPR009057 | Homeobox-like domain superfamily |
| **Prudul26A025565T1** | Upstream gene variant | Pd02 | 1,40E+07 | 1,40E+07 | recessive | AX-586048050 | GO:0005885 | Cellular Component:Arp2/3 protein complex | K05754 | actin related protein 2/3 complex, subunit 5 | IPR006789 | Actin-related protein 2/3 complex subunit 5 |
|  |  | Pd02 | 1,40E+07 | 1,40E+07 | recessive | AX-586048050 | GO:0005885 | Cellular Component:Arp2/3 protein complex | K05754 | actin related protein 2/3 complex, subunit 5 | IPR036743 | Actin-related protein 2/3 complex subunit 5 superfamily |
|  |  | Pd02 | 1,40E+07 | 1,40E+07 | recessive | AX-586048050 | GO:0015629 | Cellular Component:actin cytoskeleton | K05754 | actin related protein 2/3 complex, subunit 5 | IPR006789 | Actin-related protein 2/3 complex subunit 5 |
|  |  | Pd02 | 1,40E+07 | 1,40E+07 | recessive | AX-586048050 | GO:0015629 | Cellular Component:actin cytoskeleton | K05754 | actin related protein 2/3 complex, subunit 5 | IPR036743 | Actin-related protein 2/3 complex subunit 5 superfamily |
|  |  | Pd02 | 1,40E+07 | 1,40E+07 | recessive | AX-586048050 | GO:0034314 | Biological Process:Arp2/3 complex-mediated actin nucleation | K05754 | actin related protein 2/3 complex, subunit 5 | IPR006789 | Actin-related protein 2/3 complex subunit 5 |
|  |  | Pd02 | 1,40E+07 | 1,40E+07 | recessive | AX-586048050 | GO:0034314 | Biological Process:Arp2/3 complex-mediated actin nucleation | K05754 | actin related protein 2/3 complex, subunit 5 | IPR036743 | Actin-related protein 2/3 complex subunit 5 superfamily |
|  |  | Pd02 | 1,40E+07 | 1,40E+07 | recessive | AX-586048050 | GO:0030833 | Biological Process:regulation of actin filament polymerization | K05754 | actin related protein 2/3 complex, subunit 5 | IPR006789 | Actin-related protein 2/3 complex subunit 5 |
|  |  | Pd02 | 1,40E+07 | 1,40E+07 | recessive | AX-586048050 | GO:0030833 | Biological Process:regulation of actin filament polymerization | K05754 | actin related protein 2/3 complex, subunit 5 | IPR036743 | Actin-related protein 2/3 complex subunit 5 superfamily |
| **Prudul26A007411T1** |  | Pd02 | 1,40E+07 | 1,40E+07 | recessive | AX-586048050 | NA | NA | NA | NA | IPR008480 | Protein of unknown function DUF761, plant |
| **Prudul26A012633T1** | Upstream gene variant | Pd02 | 1,40E+07 | 1,40E+07 | recessive | AX-586048050 | NA | NA | NA | NA | IPR023566 | Peptidyl-prolyl cis-trans isomerase, FKBP-type |
|  |  | Pd02 | 1,40E+07 | 1,40E+07 | recessive | AX-586048050 | NA | NA | NA | NA | IPR001179 | FKBP-type peptidyl-prolyl cis-trans isomerase domain |
| **Prudul26A013443T1** |  | Pd02 | 1,40E+07 | 1,40E+07 | recessive | AX-586048050 | GO:0055085 | Biological Process:transmembrane transport | NA | NA | IPR002293 | Amino acid/polyamine transporter I |
|  |  | Pd02 | 1,40E+07 | 1,40E+07 | recessive | AX-586048050 | GO:0022857 | Molecular Function:transmembrane transporter activity | NA | NA | IPR002293 | Amino acid/polyamine transporter I |
|  |  | Pd02 | 1,40E+07 | 1,40E+07 | recessive | AX-586048050 | GO:0016020 | Cellular Component:membrane | NA | NA | IPR002293 | Amino acid/polyamine transporter I |
| **Prudul26A013443T2** |  | Pd02 | 1,40E+07 | 1,40E+07 | recessive | AX-586048050 | GO:0022857 | Molecular Function:transmembrane transporter activity | NA | NA | IPR002293 | Amino acid/polyamine transporter I |
|  |  | Pd02 | 1,40E+07 | 1,40E+07 | recessive | AX-586048050 | GO:0055085 | Biological Process:transmembrane transport | NA | NA | IPR002293 | Amino acid/polyamine transporter I |
|  |  | Pd02 | 1,40E+07 | 1,40E+07 | recessive | AX-586048050 | GO:0016020 | Cellular Component:membrane | NA | NA | IPR002293 | Amino acid/polyamine transporter I |
| **Prudul26A006053T1** |  | Pd02 | 1,40E+07 | 1,40E+07 | recessive | AX-586048050 | GO:0005515 | Molecular Function:protein binding | NA | NA | IPR000225 | Armadillo |
|  |  | Pd02 | 1,40E+07 | 1,40E+07 | recessive | AX-586048050 | GO:0005515 | Molecular Function:protein binding | NA | NA | IPR016024 | Armadillo-type fold |
|  |  | Pd02 | 1,40E+07 | 1,40E+07 | recessive | AX-586048050 | GO:0005515 | Molecular Function:protein binding | NA | NA | IPR032675 | Leucine-rich repeat domain superfamily |
|  |  | Pd02 | 1,40E+07 | 1,40E+07 | recessive | AX-586048050 | GO:0005515 | Molecular Function:protein binding | NA | NA | IPR011989 | Armadillo-like helical |
|  |  | Pd02 | 1,40E+07 | 1,40E+07 | recessive | AX-586048050 | GO:0005515 | Molecular Function:protein binding | NA | NA | IPR001810 | F-box domain |
| **Prudul26A026067T1** |  | Pd02 | 1,40E+07 | 1,40E+07 | recessive | AX-586048050 | GO:0098599 | Molecular Function:palmitoyl hydrolase activity | K01074 | palmitoyl-protein thioesterase [EC:3.1.2.22] | IPR002472 | Palmitoyl protein thioesterase |
|  |  | Pd02 | 1,40E+07 | 1,40E+07 | recessive | AX-586048050 | GO:0098599 | Molecular Function:palmitoyl hydrolase activity | K01074 | palmitoyl-protein thioesterase [EC:3.1.2.22] | IPR029058 | Alpha/Beta hydrolase fold |
| **Prudul26A018763T1** |  | Pd02 | 1,40E+07 | 1,40E+07 | recessive | AX-586048050 | NA | NA | NA | NA | NA | NA |
| **Prudul26A018763T2** |  | Pd02 | 1,40E+07 | 1,40E+07 | recessive | AX-586048050 | NA | NA | NA | NA | NA | NA |
| **Prudul26A018763T3** |  | Pd02 | 1,40E+07 | 1,40E+07 | recessive | AX-586048050 | NA | NA | NA | NA | NA | NA |
| **Prudul26A006068T1** |  | Pd03 | 1,50E+07 | 1,50E+07 | dominance | AX-586069960 | GO:0015267 | Molecular Function:channel activity | NA | NA | IPR000425 | Major intrinsic protein |
|  |  | Pd03 | 1,50E+07 | 1,50E+07 | dominance | AX-586069960 | GO:0015267 | Molecular Function:channel activity | NA | NA | IPR022357 | Major intrinsic protein, conserved site |
|  |  | Pd03 | 1,50E+07 | 1,50E+07 | dominance | AX-586069960 | GO:0015267 | Molecular Function:channel activity | NA | NA | IPR034294 | Aquaporin transporter |
|  |  | Pd03 | 1,50E+07 | 1,50E+07 | dominance | AX-586069960 | GO:0015267 | Molecular Function:channel activity | NA | NA | IPR023271 | Aquaporin-like |
|  |  | Pd03 | 1,50E+07 | 1,50E+07 | dominance | AX-586069960 | GO:0055085 | Biological Process:transmembrane transport | NA | NA | IPR000425 | Major intrinsic protein |
|  |  | Pd03 | 1,50E+07 | 1,50E+07 | dominance | AX-586069960 | GO:0055085 | Biological Process:transmembrane transport | NA | NA | IPR022357 | Major intrinsic protein, conserved site |
|  |  | Pd03 | 1,50E+07 | 1,50E+07 | dominance | AX-586069960 | GO:0055085 | Biological Process:transmembrane transport | NA | NA | IPR034294 | Aquaporin transporter |
|  |  | Pd03 | 1,50E+07 | 1,50E+07 | dominance | AX-586069960 | GO:0055085 | Biological Process:transmembrane transport | NA | NA | IPR023271 | Aquaporin-like |
|  |  | Pd03 | 1,50E+07 | 1,50E+07 | dominance | AX-586069960 | GO:0016020 | Cellular Component:membrane | NA | NA | IPR000425 | Major intrinsic protein |
|  |  | Pd03 | 1,50E+07 | 1,50E+07 | dominance | AX-586069960 | GO:0016020 | Cellular Component:membrane | NA | NA | IPR022357 | Major intrinsic protein, conserved site |
|  |  | Pd03 | 1,50E+07 | 1,50E+07 | dominance | AX-586069960 | GO:0016020 | Cellular Component:membrane | NA | NA | IPR034294 | Aquaporin transporter |
|  |  | Pd03 | 1,50E+07 | 1,50E+07 | dominance | AX-586069960 | GO:0016020 | Cellular Component:membrane | NA | NA | IPR023271 | Aquaporin-like |
| **Prudul26A005769T1** |  | Pd03 | 1,50E+07 | 1,50E+07 | dominance | AX-586069960 | GO:0005515 | Molecular Function:protein binding | NA | NA | IPR000225 | Armadillo |
|  |  | Pd03 | 1,50E+07 | 1,50E+07 | dominance | AX-586069960 | GO:0005515 | Molecular Function:protein binding | NA | NA | IPR016024 | Armadillo-type fold |
|  |  | Pd03 | 1,50E+07 | 1,50E+07 | dominance | AX-586069960 | GO:0005515 | Molecular Function:protein binding | NA | NA | IPR011989 | Armadillo-like helical |
| **Prudul26A031948T1** |  | Pd03 | 1,50E+07 | 1,50E+07 | dominance | AX-586069960 | GO:0046983 | Molecular Function:protein dimerization activity | NA | NA | IPR036638 | Helix-loop-helix DNA-binding domain superfamily |
|  |  | Pd03 | 1,50E+07 | 1,50E+07 | dominance | AX-586069960 | GO:0046983 | Molecular Function:protein dimerization activity | NA | NA | IPR011598 | Myc-type, basic helix-loop-helix (bHLH) domain |
| **Prudul26A024244T1** |  | Pd03 | 1,50E+07 | 1,50E+07 | dominance | AX-586069960 | GO:0006508 | Biological Process:proteolysis | NA | NA | IPR003653 | Ulp1 protease family, C-terminal catalytic domain |
|  |  | Pd03 | 1,50E+07 | 1,50E+07 | dominance | AX-586069960 | GO:0008234 | Molecular Function:cysteine-type peptidase activity | NA | NA | IPR003653 | Ulp1 protease family, C-terminal catalytic domain |
| **Prudul26A022902T1** |  | Pd03 | 1,50E+07 | 1,50E+07 | dominance | AX-586069960 | GO:0016747 | Molecular Function:transferase activity, transferring acyl groups other than amino-acyl groups | K19861 | benzyl alcohol O-benzoyltransferase [EC:2.3.1.196 2.3.1.232] | IPR023213 | Chloramphenicol acetyltransferase-like domain superfamily |
|  |  | Pd03 | 1,50E+07 | 1,50E+07 | dominance | AX-586069960 | GO:0016747 | Molecular Function:transferase activity, transferring acyl groups other than amino-acyl groups | K19861 | benzyl alcohol O-benzoyltransferase [EC:2.3.1.196 2.3.1.232] | IPR003480 | Transferase |
| **Prudul26A017598T1** |  | Pd03 | 1,50E+07 | 1,50E+07 | overdominance | AX-586070824 | GO:0005515 | Molecular Function:protein binding | NA | NA | IPR002885 | Pentatricopeptide repeat |
|  |  | Pd03 | 1,50E+07 | 1,50E+07 | overdominance | AX-586070824 | GO:0005515 | Molecular Function:protein binding | NA | NA | IPR011990 | Tetratricopeptide-like helical domain superfamily |
|  |  | Pd03 | 1,50E+07 | 1,50E+07 | dominance | AX-586069960 | GO:0005515 | Molecular Function:protein binding | NA | NA | IPR002885 | Pentatricopeptide repeat |
|  |  | Pd03 | 1,50E+07 | 1,50E+07 | dominance | AX-586069960 | GO:0005515 | Molecular Function:protein binding | NA | NA | IPR011990 | Tetratricopeptide-like helical domain superfamily |
| **Prudul26A027971T1** | Downstream gene variant | Pd03 | 1,50E+07 | 1,50E+07 | overdominance | AX-586070824 | GO:0005524 | Molecular Function:ATP binding | NA | NA | IPR000719 | Protein kinase domain |
|  |  | Pd03 | 1,50E+07 | 1,50E+07 | overdominance | AX-586070824 | GO:0005524 | Molecular Function:ATP binding | NA | NA | IPR008271 | Serine/threonine-protein kinase, active site |
|  |  | Pd03 | 1,50E+07 | 1,50E+07 | overdominance | AX-586070824 | GO:0005524 | Molecular Function:ATP binding | NA | NA | IPR011009 | Protein kinase-like domain superfamily |
|  |  | Pd03 | 1,50E+07 | 1,50E+07 | overdominance | AX-586070824 | GO:0006468 | Biological Process:protein phosphorylation | NA | NA | IPR000719 | Protein kinase domain |
|  |  | Pd03 | 1,50E+07 | 1,50E+07 | overdominance | AX-586070824 | GO:0006468 | Biological Process:protein phosphorylation | NA | NA | IPR008271 | Serine/threonine-protein kinase, active site |
|  |  | Pd03 | 1,50E+07 | 1,50E+07 | overdominance | AX-586070824 | GO:0006468 | Biological Process:protein phosphorylation | NA | NA | IPR011009 | Protein kinase-like domain superfamily |
|  |  | Pd03 | 1,50E+07 | 1,50E+07 | overdominance | AX-586070824 | GO:0004672 | Molecular Function:protein kinase activity | NA | NA | IPR000719 | Protein kinase domain |
|  |  | Pd03 | 1,50E+07 | 1,50E+07 | overdominance | AX-586070824 | GO:0004672 | Molecular Function:protein kinase activity | NA | NA | IPR008271 | Serine/threonine-protein kinase, active site |
|  |  | Pd03 | 1,50E+07 | 1,50E+07 | overdominance | AX-586070824 | GO:0004672 | Molecular Function:protein kinase activity | NA | NA | IPR011009 | Protein kinase-like domain superfamily |
|  |  | Pd03 | 1,50E+07 | 1,50E+07 | dominance | AX-586069960 | GO:0005524 | Molecular Function:ATP binding | NA | NA | IPR000719 | Protein kinase domain |
|  |  | Pd03 | 1,50E+07 | 1,50E+07 | dominance | AX-586069960 | GO:0005524 | Molecular Function:ATP binding | NA | NA | IPR008271 | Serine/threonine-protein kinase, active site |
|  |  | Pd03 | 1,50E+07 | 1,50E+07 | dominance | AX-586069960 | GO:0005524 | Molecular Function:ATP binding | NA | NA | IPR011009 | Protein kinase-like domain superfamily |
|  |  | Pd03 | 1,50E+07 | 1,50E+07 | dominance | AX-586069960 | GO:0006468 | Biological Process:protein phosphorylation | NA | NA | IPR000719 | Protein kinase domain |
|  |  | Pd03 | 1,50E+07 | 1,50E+07 | dominance | AX-586069960 | GO:0006468 | Biological Process:protein phosphorylation | NA | NA | IPR008271 | Serine/threonine-protein kinase, active site |
|  |  | Pd03 | 1,50E+07 | 1,50E+07 | dominance | AX-586069960 | GO:0006468 | Biological Process:protein phosphorylation | NA | NA | IPR011009 | Protein kinase-like domain superfamily |
|  |  | Pd03 | 1,50E+07 | 1,50E+07 | dominance | AX-586069960 | GO:0004672 | Molecular Function:protein kinase activity | NA | NA | IPR000719 | Protein kinase domain |
|  |  | Pd03 | 1,50E+07 | 1,50E+07 | dominance | AX-586069960 | GO:0004672 | Molecular Function:protein kinase activity | NA | NA | IPR008271 | Serine/threonine-protein kinase, active site |
|  |  | Pd03 | 1,50E+07 | 1,50E+07 | dominance | AX-586069960 | GO:0004672 | Molecular Function:protein kinase activity | NA | NA | IPR011009 | Protein kinase-like domain superfamily |
| **Prudul26A002745T1** | Downstream gene variant | Pd03 | 1,50E+07 | 1,50E+07 | overdominance | AX-586070824 | GO:0016773 | Molecular Function:phosphotransferase activity, alcohol group as acceptor | K00847 | fructokinase [EC:2.7.1.4] | IPR011611 | Carbohydrate kinase PfkB |
|  |  | Pd03 | 1,50E+07 | 1,50E+07 | overdominance | AX-586070824 | GO:0016773 | Molecular Function:phosphotransferase activity, alcohol group as acceptor | K00847 | fructokinase [EC:2.7.1.4] | IPR002173 | Carbohydrate/puine kinase, PfkB, conserved site |
|  |  | Pd03 | 1,50E+07 | 1,50E+07 | overdominance | AX-586070824 | GO:0016773 | Molecular Function:phosphotransferase activity, alcohol group as acceptor | K00847 | fructokinase [EC:2.7.1.4] | IPR002139 | Ribokinase/fructokinase |
|  |  | Pd03 | 1,50E+07 | 1,50E+07 | overdominance | AX-586070824 | GO:0016773 | Molecular Function:phosphotransferase activity, alcohol group as acceptor | K00847 | fructokinase [EC:2.7.1.4] | IPR029056 | Ribokinase-like |
|  |  | Pd03 | 1,50E+07 | 1,50E+07 | overdominance | AX-586070824 | GO:0016301 | Molecular Function:kinase activity | K00847 | fructokinase [EC:2.7.1.4] | IPR011611 | Carbohydrate kinase PfkB |
|  |  | Pd03 | 1,50E+07 | 1,50E+07 | overdominance | AX-586070824 | GO:0016301 | Molecular Function:kinase activity | K00847 | fructokinase [EC:2.7.1.4] | IPR002173 | Carbohydrate/puine kinase, PfkB, conserved site |
|  |  | Pd03 | 1,50E+07 | 1,50E+07 | overdominance | AX-586070824 | GO:0016301 | Molecular Function:kinase activity | K00847 | fructokinase [EC:2.7.1.4] | IPR002139 | Ribokinase/fructokinase |
|  |  | Pd03 | 1,50E+07 | 1,50E+07 | overdominance | AX-586070824 | GO:0016301 | Molecular Function:kinase activity | K00847 | fructokinase [EC:2.7.1.4] | IPR029056 | Ribokinase-like |
|  |  | Pd03 | 1,50E+07 | 1,50E+07 | dominance | AX-586069960 | GO:0016773 | Molecular Function:phosphotransferase activity, alcohol group as acceptor | K00847 | fructokinase [EC:2.7.1.4] | IPR011611 | Carbohydrate kinase PfkB |
|  |  | Pd03 | 1,50E+07 | 1,50E+07 | dominance | AX-586069960 | GO:0016773 | Molecular Function:phosphotransferase activity, alcohol group as acceptor | K00847 | fructokinase [EC:2.7.1.4] | IPR002173 | Carbohydrate/puine kinase, PfkB, conserved site |
|  |  | Pd03 | 1,50E+07 | 1,50E+07 | dominance | AX-586069960 | GO:0016773 | Molecular Function:phosphotransferase activity, alcohol group as acceptor | K00847 | fructokinase [EC:2.7.1.4] | IPR002139 | Ribokinase/fructokinase |
|  |  | Pd03 | 1,50E+07 | 1,50E+07 | dominance | AX-586069960 | GO:0016773 | Molecular Function:phosphotransferase activity, alcohol group as acceptor | K00847 | fructokinase [EC:2.7.1.4] | IPR029056 | Ribokinase-like |
|  |  | Pd03 | 1,50E+07 | 1,50E+07 | dominance | AX-586069960 | GO:0016301 | Molecular Function:kinase activity | K00847 | fructokinase [EC:2.7.1.4] | IPR011611 | Carbohydrate kinase PfkB |
|  |  | Pd03 | 1,50E+07 | 1,50E+07 | dominance | AX-586069960 | GO:0016301 | Molecular Function:kinase activity | K00847 | fructokinase [EC:2.7.1.4] | IPR002173 | Carbohydrate/puine kinase, PfkB, conserved site |
|  |  | Pd03 | 1,50E+07 | 1,50E+07 | dominance | AX-586069960 | GO:0016301 | Molecular Function:kinase activity | K00847 | fructokinase [EC:2.7.1.4] | IPR002139 | Ribokinase/fructokinase |
|  |  | Pd03 | 1,50E+07 | 1,50E+07 | dominance | AX-586069960 | GO:0016301 | Molecular Function:kinase activity | K00847 | fructokinase [EC:2.7.1.4] | IPR029056 | Ribokinase-like |
| **Prudul26A022503T1** |  | Pd03 | 1,50E+07 | 1,50E+07 | overdominance | AX-586070824 | GO:0005509 | Molecular Function:calcium ion binding | NA | NA | IPR039647 | EF-hand domain pair protein CML-like |
|  |  | Pd03 | 1,50E+07 | 1,50E+07 | overdominance | AX-586070824 | GO:0005509 | Molecular Function:calcium ion binding | NA | NA | IPR002048 | EF-hand domain |
|  |  | Pd03 | 1,50E+07 | 1,50E+07 | overdominance | AX-586070824 | GO:0005509 | Molecular Function:calcium ion binding | NA | NA | IPR018247 | EF-Hand 1, calcium-binding site |
|  |  | Pd03 | 1,50E+07 | 1,50E+07 | overdominance | AX-586070824 | GO:0005509 | Molecular Function:calcium ion binding | NA | NA | IPR011992 | EF-hand domain pair |
|  |  | Pd03 | 1,50E+07 | 1,50E+07 | dominance | AX-586069960 | GO:0005509 | Molecular Function:calcium ion binding | NA | NA | IPR039647 | EF-hand domain pair protein CML-like |
|  |  | Pd03 | 1,50E+07 | 1,50E+07 | dominance | AX-586069960 | GO:0005509 | Molecular Function:calcium ion binding | NA | NA | IPR002048 | EF-hand domain |
|  |  | Pd03 | 1,50E+07 | 1,50E+07 | dominance | AX-586069960 | GO:0005509 | Molecular Function:calcium ion binding | NA | NA | IPR018247 | EF-Hand 1, calcium-binding site |
|  |  | Pd03 | 1,50E+07 | 1,50E+07 | dominance | AX-586069960 | GO:0005509 | Molecular Function:calcium ion binding | NA | NA | IPR011992 | EF-hand domain pair |
| **Prudul26A021070T1** |  | Pd03 | 1,50E+07 | 1,50E+07 | dominance | AX-586069960 | NA | NA | NA | NA | IPR031318 | OPI10 family |
|  |  | Pd03 | 1,50E+07 | 1,50E+07 | dominance | AX-586069960 | NA | NA | NA | NA | IPR008493 | Domain of unknown function DUF775 |
|  |  | Pd03 | 1,50E+07 | 1,50E+07 | overdominance | AX-586070824 | NA | NA | NA | NA | IPR031318 | OPI10 family |
|  |  | Pd03 | 1,50E+07 | 1,50E+07 | overdominance | AX-586070824 | NA | NA | NA | NA | IPR008493 | Domain of unknown function DUF775 |
| **Prudul26A032316T1** | Intron variant | Pd03 | 1,50E+07 | 1,50E+07 | dominance | AX-586069960 | GO:0003676 | Molecular Function:nucleic acid binding | K12881 | THO complex subunit 4 | IPR035979 | RNA-binding domain superfamily |
|  |  | Pd03 | 1,50E+07 | 1,50E+07 | dominance | AX-586069960 | GO:0003676 | Molecular Function:nucleic acid binding | K12881 | THO complex subunit 4 | IPR012677 | Nucleotide-binding alpha-beta plait domain superfamily |
|  |  | Pd03 | 1,50E+07 | 1,50E+07 | dominance | AX-586069960 | GO:0003676 | Molecular Function:nucleic acid binding | K12881 | THO complex subunit 4 | IPR025715 | Chromatin target of PRMT1 protein, C-terminal |
|  |  | Pd03 | 1,50E+07 | 1,50E+07 | dominance | AX-586069960 | GO:0003676 | Molecular Function:nucleic acid binding | K12881 | THO complex subunit 4 | IPR000504 | RNA recognition motif domain |
|  |  | Pd03 | 1,50E+07 | 1,50E+07 | overdominance | AX-586070824 | GO:0003676 | Molecular Function:nucleic acid binding | K12881 | THO complex subunit 4 | IPR035979 | RNA-binding domain superfamily |
|  |  | Pd03 | 1,50E+07 | 1,50E+07 | overdominance | AX-586070824 | GO:0003676 | Molecular Function:nucleic acid binding | K12881 | THO complex subunit 4 | IPR012677 | Nucleotide-binding alpha-beta plait domain superfamily |
|  |  | Pd03 | 1,50E+07 | 1,50E+07 | overdominance | AX-586070824 | GO:0003676 | Molecular Function:nucleic acid binding | K12881 | THO complex subunit 4 | IPR025715 | Chromatin target of PRMT1 protein, C-terminal |
|  |  | Pd03 | 1,50E+07 | 1,50E+07 | overdominance | AX-586070824 | GO:0003676 | Molecular Function:nucleic acid binding | K12881 | THO complex subunit 4 | IPR000504 | RNA recognition motif domain |
| **Prudul26A001790T1** |  | Pd03 | 1,50E+07 | 1,50E+07 | dominance | AX-586069960 | NA | NA | NA | NA | NA | NA |
|  |  | Pd03 | 1,50E+07 | 1,50E+07 | overdominance | AX-586070824 | NA | NA | NA | NA | NA | NA |
| **Prudul26A008886T1** |  | Pd03 | 1,50E+07 | 1,50E+07 | overdominance | AX-586070824 | GO:0006289 | Biological Process:nucleotide-excision repair | K10838 | xeroderma pigmentosum group C-complementing protein | IPR018328 | Rad4 beta-hairpin domain 3 |
|  |  | Pd03 | 1,50E+07 | 1,50E+07 | overdominance | AX-586070824 | GO:0006289 | Biological Process:nucleotide-excision repair | K10838 | xeroderma pigmentosum group C-complementing protein | IPR004583 | DNA repair protein Rad4 |
|  |  | Pd03 | 1,50E+07 | 1,50E+07 | overdominance | AX-586070824 | GO:0006289 | Biological Process:nucleotide-excision repair | K10838 | xeroderma pigmentosum group C-complementing protein | IPR018327 | Rad4 beta-hairpin domain 2 |
|  |  | Pd03 | 1,50E+07 | 1,50E+07 | overdominance | AX-586070824 | GO:0006289 | Biological Process:nucleotide-excision repair | K10838 | xeroderma pigmentosum group C-complementing protein | IPR036985 | Transglutaminase-like superfamily |
|  |  | Pd03 | 1,50E+07 | 1,50E+07 | overdominance | AX-586070824 | GO:0006289 | Biological Process:nucleotide-excision repair | K10838 | xeroderma pigmentosum group C-complementing protein | IPR018326 | Rad4 beta-hairpin domain 1 |
|  |  | Pd03 | 1,50E+07 | 1,50E+07 | overdominance | AX-586070824 | GO:0006289 | Biological Process:nucleotide-excision repair | K10838 | xeroderma pigmentosum group C-complementing protein | IPR038765 | Papain-like cysteine peptidase superfamily |
|  |  | Pd03 | 1,50E+07 | 1,50E+07 | overdominance | AX-586070824 | GO:0006289 | Biological Process:nucleotide-excision repair | K10838 | xeroderma pigmentosum group C-complementing protein | IPR002931 | Transglutaminase-like |
|  |  | Pd03 | 1,50E+07 | 1,50E+07 | overdominance | AX-586070824 | GO:0006289 | Biological Process:nucleotide-excision repair | K10838 | xeroderma pigmentosum group C-complementing protein | IPR018325 | Rad4/PNGase transglutaminase-like fold |
|  |  | Pd03 | 1,50E+07 | 1,50E+07 | overdominance | AX-586070824 | GO:0003684 | Molecular Function:damaged DNA binding | K10838 | xeroderma pigmentosum group C-complementing protein | IPR018328 | Rad4 beta-hairpin domain 3 |
|  |  | Pd03 | 1,50E+07 | 1,50E+07 | overdominance | AX-586070824 | GO:0003684 | Molecular Function:damaged DNA binding | K10838 | xeroderma pigmentosum group C-complementing protein | IPR004583 | DNA repair protein Rad4 |
|  |  | Pd03 | 1,50E+07 | 1,50E+07 | overdominance | AX-586070824 | GO:0003684 | Molecular Function:damaged DNA binding | K10838 | xeroderma pigmentosum group C-complementing protein | IPR018327 | Rad4 beta-hairpin domain 2 |
|  |  | Pd03 | 1,50E+07 | 1,50E+07 | overdominance | AX-586070824 | GO:0003684 | Molecular Function:damaged DNA binding | K10838 | xeroderma pigmentosum group C-complementing protein | IPR036985 | Transglutaminase-like superfamily |
|  |  | Pd03 | 1,50E+07 | 1,50E+07 | overdominance | AX-586070824 | GO:0003684 | Molecular Function:damaged DNA binding | K10838 | xeroderma pigmentosum group C-complementing protein | IPR018326 | Rad4 beta-hairpin domain 1 |
|  |  | Pd03 | 1,50E+07 | 1,50E+07 | overdominance | AX-586070824 | GO:0003684 | Molecular Function:damaged DNA binding | K10838 | xeroderma pigmentosum group C-complementing protein | IPR038765 | Papain-like cysteine peptidase superfamily |
|  |  | Pd03 | 1,50E+07 | 1,50E+07 | overdominance | AX-586070824 | GO:0003684 | Molecular Function:damaged DNA binding | K10838 | xeroderma pigmentosum group C-complementing protein | IPR002931 | Transglutaminase-like |
|  |  | Pd03 | 1,50E+07 | 1,50E+07 | overdominance | AX-586070824 | GO:0003684 | Molecular Function:damaged DNA binding | K10838 | xeroderma pigmentosum group C-complementing protein | IPR018325 | Rad4/PNGase transglutaminase-like fold |
|  |  | Pd03 | 1,50E+07 | 1,50E+07 | overdominance | AX-586070824 | GO:0005634 | Cellular Component:nucleus | K10838 | xeroderma pigmentosum group C-complementing protein | IPR018328 | Rad4 beta-hairpin domain 3 |
|  |  | Pd03 | 1,50E+07 | 1,50E+07 | overdominance | AX-586070824 | GO:0005634 | Cellular Component:nucleus | K10838 | xeroderma pigmentosum group C-complementing protein | IPR004583 | DNA repair protein Rad4 |
|  |  | Pd03 | 1,50E+07 | 1,50E+07 | overdominance | AX-586070824 | GO:0005634 | Cellular Component:nucleus | K10838 | xeroderma pigmentosum group C-complementing protein | IPR018327 | Rad4 beta-hairpin domain 2 |
|  |  | Pd03 | 1,50E+07 | 1,50E+07 | overdominance | AX-586070824 | GO:0005634 | Cellular Component:nucleus | K10838 | xeroderma pigmentosum group C-complementing protein | IPR036985 | Transglutaminase-like superfamily |
|  |  | Pd03 | 1,50E+07 | 1,50E+07 | overdominance | AX-586070824 | GO:0005634 | Cellular Component:nucleus | K10838 | xeroderma pigmentosum group C-complementing protein | IPR018326 | Rad4 beta-hairpin domain 1 |
|  |  | Pd03 | 1,50E+07 | 1,50E+07 | overdominance | AX-586070824 | GO:0005634 | Cellular Component:nucleus | K10838 | xeroderma pigmentosum group C-complementing protein | IPR038765 | Papain-like cysteine peptidase superfamily |
|  |  | Pd03 | 1,50E+07 | 1,50E+07 | overdominance | AX-586070824 | GO:0005634 | Cellular Component:nucleus | K10838 | xeroderma pigmentosum group C-complementing protein | IPR002931 | Transglutaminase-like |
|  |  | Pd03 | 1,50E+07 | 1,50E+07 | overdominance | AX-586070824 | GO:0005634 | Cellular Component:nucleus | K10838 | xeroderma pigmentosum group C-complementing protein | IPR018325 | Rad4/PNGase transglutaminase-like fold |
|  |  | Pd03 | 1,50E+07 | 1,50E+07 | overdominance | AX-586070824 | GO:0003677 | Molecular Function:DNA binding | K10838 | xeroderma pigmentosum group C-complementing protein | IPR018328 | Rad4 beta-hairpin domain 3 |
|  |  | Pd03 | 1,50E+07 | 1,50E+07 | overdominance | AX-586070824 | GO:0003677 | Molecular Function:DNA binding | K10838 | xeroderma pigmentosum group C-complementing protein | IPR004583 | DNA repair protein Rad4 |
|  |  | Pd03 | 1,50E+07 | 1,50E+07 | overdominance | AX-586070824 | GO:0003677 | Molecular Function:DNA binding | K10838 | xeroderma pigmentosum group C-complementing protein | IPR018327 | Rad4 beta-hairpin domain 2 |
|  |  | Pd03 | 1,50E+07 | 1,50E+07 | overdominance | AX-586070824 | GO:0003677 | Molecular Function:DNA binding | K10838 | xeroderma pigmentosum group C-complementing protein | IPR036985 | Transglutaminase-like superfamily |
|  |  | Pd03 | 1,50E+07 | 1,50E+07 | overdominance | AX-586070824 | GO:0003677 | Molecular Function:DNA binding | K10838 | xeroderma pigmentosum group C-complementing protein | IPR018326 | Rad4 beta-hairpin domain 1 |
|  |  | Pd03 | 1,50E+07 | 1,50E+07 | overdominance | AX-586070824 | GO:0003677 | Molecular Function:DNA binding | K10838 | xeroderma pigmentosum group C-complementing protein | IPR038765 | Papain-like cysteine peptidase superfamily |
|  |  | Pd03 | 1,50E+07 | 1,50E+07 | overdominance | AX-586070824 | GO:0003677 | Molecular Function:DNA binding | K10838 | xeroderma pigmentosum group C-complementing protein | IPR002931 | Transglutaminase-like |
|  |  | Pd03 | 1,50E+07 | 1,50E+07 | overdominance | AX-586070824 | GO:0003677 | Molecular Function:DNA binding | K10838 | xeroderma pigmentosum group C-complementing protein | IPR018325 | Rad4/PNGase transglutaminase-like fold |
| **Prudul26A021349T1** |  | Pd03 | 1,50E+07 | 1,50E+07 | overdominance | AX-586070824 | GO:0000786 | Cellular Component:nucleosome | NA | NA | IPR036390 | Winged helix DNA-binding domain superfamily |
|  |  | Pd03 | 1,50E+07 | 1,50E+07 | overdominance | AX-586070824 | GO:0000786 | Cellular Component:nucleosome | NA | NA | IPR036388 | Winged helix-like DNA-binding domain superfamily |
|  |  | Pd03 | 1,50E+07 | 1,50E+07 | overdominance | AX-586070824 | GO:0000786 | Cellular Component:nucleosome | NA | NA | IPR000116 | High mobility group protein HMGA |
|  |  | Pd03 | 1,50E+07 | 1,50E+07 | overdominance | AX-586070824 | GO:0000786 | Cellular Component:nucleosome | NA | NA | IPR031059 | High mobility group protein HMGA, plant |
|  |  | Pd03 | 1,50E+07 | 1,50E+07 | overdominance | AX-586070824 | GO:0000786 | Cellular Component:nucleosome | NA | NA | IPR005818 | Linker histone H1/H5, domain H15 |
|  |  | Pd03 | 1,50E+07 | 1,50E+07 | overdominance | AX-586070824 | GO:0000786 | Cellular Component:nucleosome | NA | NA | IPR017956 | AT hook, DNA-binding motif |
|  |  | Pd03 | 1,50E+07 | 1,50E+07 | overdominance | AX-586070824 | GO:0005634 | Cellular Component:nucleus | NA | NA | IPR036390 | Winged helix DNA-binding domain superfamily |
|  |  | Pd03 | 1,50E+07 | 1,50E+07 | overdominance | AX-586070824 | GO:0005634 | Cellular Component:nucleus | NA | NA | IPR036388 | Winged helix-like DNA-binding domain superfamily |
|  |  | Pd03 | 1,50E+07 | 1,50E+07 | overdominance | AX-586070824 | GO:0005634 | Cellular Component:nucleus | NA | NA | IPR000116 | High mobility group protein HMGA |
|  |  | Pd03 | 1,50E+07 | 1,50E+07 | overdominance | AX-586070824 | GO:0005634 | Cellular Component:nucleus | NA | NA | IPR031059 | High mobility group protein HMGA, plant |
|  |  | Pd03 | 1,50E+07 | 1,50E+07 | overdominance | AX-586070824 | GO:0005634 | Cellular Component:nucleus | NA | NA | IPR005818 | Linker histone H1/H5, domain H15 |
|  |  | Pd03 | 1,50E+07 | 1,50E+07 | overdominance | AX-586070824 | GO:0005634 | Cellular Component:nucleus | NA | NA | IPR017956 | AT hook, DNA-binding motif |
|  |  | Pd03 | 1,50E+07 | 1,50E+07 | overdominance | AX-586070824 | GO:0003677 | Molecular Function:DNA binding | NA | NA | IPR036390 | Winged helix DNA-binding domain superfamily |
|  |  | Pd03 | 1,50E+07 | 1,50E+07 | overdominance | AX-586070824 | GO:0003677 | Molecular Function:DNA binding | NA | NA | IPR036388 | Winged helix-like DNA-binding domain superfamily |
|  |  | Pd03 | 1,50E+07 | 1,50E+07 | overdominance | AX-586070824 | GO:0003677 | Molecular Function:DNA binding | NA | NA | IPR000116 | High mobility group protein HMGA |
|  |  | Pd03 | 1,50E+07 | 1,50E+07 | overdominance | AX-586070824 | GO:0003677 | Molecular Function:DNA binding | NA | NA | IPR031059 | High mobility group protein HMGA, plant |
|  |  | Pd03 | 1,50E+07 | 1,50E+07 | overdominance | AX-586070824 | GO:0003677 | Molecular Function:DNA binding | NA | NA | IPR005818 | Linker histone H1/H5, domain H15 |
|  |  | Pd03 | 1,50E+07 | 1,50E+07 | overdominance | AX-586070824 | GO:0003677 | Molecular Function:DNA binding | NA | NA | IPR017956 | AT hook, DNA-binding motif |
|  |  | Pd03 | 1,50E+07 | 1,50E+07 | overdominance | AX-586070824 | GO:0006355 | Biological Process:regulation of transcription, DNA-templated | NA | NA | IPR036390 | Winged helix DNA-binding domain superfamily |
|  |  | Pd03 | 1,50E+07 | 1,50E+07 | overdominance | AX-586070824 | GO:0006355 | Biological Process:regulation of transcription, DNA-templated | NA | NA | IPR036388 | Winged helix-like DNA-binding domain superfamily |
|  |  | Pd03 | 1,50E+07 | 1,50E+07 | overdominance | AX-586070824 | GO:0006355 | Biological Process:regulation of transcription, DNA-templated | NA | NA | IPR000116 | High mobility group protein HMGA |
|  |  | Pd03 | 1,50E+07 | 1,50E+07 | overdominance | AX-586070824 | GO:0006355 | Biological Process:regulation of transcription, DNA-templated | NA | NA | IPR031059 | High mobility group protein HMGA, plant |
|  |  | Pd03 | 1,50E+07 | 1,50E+07 | overdominance | AX-586070824 | GO:0006355 | Biological Process:regulation of transcription, DNA-templated | NA | NA | IPR005818 | Linker histone H1/H5, domain H15 |
|  |  | Pd03 | 1,50E+07 | 1,50E+07 | overdominance | AX-586070824 | GO:0006355 | Biological Process:regulation of transcription, DNA-templated | NA | NA | IPR017956 | AT hook, DNA-binding motif |
|  |  | Pd03 | 1,50E+07 | 1,50E+07 | overdominance | AX-586070824 | GO:0006334 | Biological Process:nucleosome assembly | NA | NA | IPR036390 | Winged helix DNA-binding domain superfamily |
|  |  | Pd03 | 1,50E+07 | 1,50E+07 | overdominance | AX-586070824 | GO:0006334 | Biological Process:nucleosome assembly | NA | NA | IPR036388 | Winged helix-like DNA-binding domain superfamily |
|  |  | Pd03 | 1,50E+07 | 1,50E+07 | overdominance | AX-586070824 | GO:0006334 | Biological Process:nucleosome assembly | NA | NA | IPR000116 | High mobility group protein HMGA |
|  |  | Pd03 | 1,50E+07 | 1,50E+07 | overdominance | AX-586070824 | GO:0006334 | Biological Process:nucleosome assembly | NA | NA | IPR031059 | High mobility group protein HMGA, plant |
|  |  | Pd03 | 1,50E+07 | 1,50E+07 | overdominance | AX-586070824 | GO:0006334 | Biological Process:nucleosome assembly | NA | NA | IPR005818 | Linker histone H1/H5, domain H15 |
|  |  | Pd03 | 1,50E+07 | 1,50E+07 | overdominance | AX-586070824 | GO:0006334 | Biological Process:nucleosome assembly | NA | NA | IPR017956 | AT hook, DNA-binding motif |
|  |  | Pd03 | 1,50E+07 | 1,50E+07 | overdominance | AX-586070824 | GO:0000785 | Cellular Component:chromatin | NA | NA | IPR036390 | Winged helix DNA-binding domain superfamily |
|  |  | Pd03 | 1,50E+07 | 1,50E+07 | overdominance | AX-586070824 | GO:0000785 | Cellular Component:chromatin | NA | NA | IPR036388 | Winged helix-like DNA-binding domain superfamily |
|  |  | Pd03 | 1,50E+07 | 1,50E+07 | overdominance | AX-586070824 | GO:0000785 | Cellular Component:chromatin | NA | NA | IPR000116 | High mobility group protein HMGA |
|  |  | Pd03 | 1,50E+07 | 1,50E+07 | overdominance | AX-586070824 | GO:0000785 | Cellular Component:chromatin | NA | NA | IPR031059 | High mobility group protein HMGA, plant |
|  |  | Pd03 | 1,50E+07 | 1,50E+07 | overdominance | AX-586070824 | GO:0000785 | Cellular Component:chromatin | NA | NA | IPR005818 | Linker histone H1/H5, domain H15 |
|  |  | Pd03 | 1,50E+07 | 1,50E+07 | overdominance | AX-586070824 | GO:0000785 | Cellular Component:chromatin | NA | NA | IPR017956 | AT hook, DNA-binding motif |
| **Prudul26A021349T2** |  | Pd03 | 1,50E+07 | 1,50E+07 | overdominance | AX-586070824 | GO:0000785 | Cellular Component:chromatin | NA | NA | IPR036390 | Winged helix DNA-binding domain superfamily |
|  |  | Pd03 | 1,50E+07 | 1,50E+07 | overdominance | AX-586070824 | GO:0000785 | Cellular Component:chromatin | NA | NA | IPR036388 | Winged helix-like DNA-binding domain superfamily |
|  |  | Pd03 | 1,50E+07 | 1,50E+07 | overdominance | AX-586070824 | GO:0000785 | Cellular Component:chromatin | NA | NA | IPR000116 | High mobility group protein HMGA |
|  |  | Pd03 | 1,50E+07 | 1,50E+07 | overdominance | AX-586070824 | GO:0000785 | Cellular Component:chromatin | NA | NA | IPR031059 | High mobility group protein HMGA, plant |
|  |  | Pd03 | 1,50E+07 | 1,50E+07 | overdominance | AX-586070824 | GO:0000785 | Cellular Component:chromatin | NA | NA | IPR017956 | AT hook, DNA-binding motif |
|  |  | Pd03 | 1,50E+07 | 1,50E+07 | overdominance | AX-586070824 | GO:0000785 | Cellular Component:chromatin | NA | NA | IPR005818 | Linker histone H1/H5, domain H15 |
|  |  | Pd03 | 1,50E+07 | 1,50E+07 | overdominance | AX-586070824 | GO:0003677 | Molecular Function:DNA binding | NA | NA | IPR036390 | Winged helix DNA-binding domain superfamily |
|  |  | Pd03 | 1,50E+07 | 1,50E+07 | overdominance | AX-586070824 | GO:0003677 | Molecular Function:DNA binding | NA | NA | IPR036388 | Winged helix-like DNA-binding domain superfamily |
|  |  | Pd03 | 1,50E+07 | 1,50E+07 | overdominance | AX-586070824 | GO:0003677 | Molecular Function:DNA binding | NA | NA | IPR000116 | High mobility group protein HMGA |
|  |  | Pd03 | 1,50E+07 | 1,50E+07 | overdominance | AX-586070824 | GO:0003677 | Molecular Function:DNA binding | NA | NA | IPR031059 | High mobility group protein HMGA, plant |
|  |  | Pd03 | 1,50E+07 | 1,50E+07 | overdominance | AX-586070824 | GO:0003677 | Molecular Function:DNA binding | NA | NA | IPR017956 | AT hook, DNA-binding motif |
|  |  | Pd03 | 1,50E+07 | 1,50E+07 | overdominance | AX-586070824 | GO:0003677 | Molecular Function:DNA binding | NA | NA | IPR005818 | Linker histone H1/H5, domain H15 |
|  |  | Pd03 | 1,50E+07 | 1,50E+07 | overdominance | AX-586070824 | GO:0000786 | Cellular Component:nucleosome | NA | NA | IPR036390 | Winged helix DNA-binding domain superfamily |
|  |  | Pd03 | 1,50E+07 | 1,50E+07 | overdominance | AX-586070824 | GO:0000786 | Cellular Component:nucleosome | NA | NA | IPR036388 | Winged helix-like DNA-binding domain superfamily |
|  |  | Pd03 | 1,50E+07 | 1,50E+07 | overdominance | AX-586070824 | GO:0000786 | Cellular Component:nucleosome | NA | NA | IPR000116 | High mobility group protein HMGA |
|  |  | Pd03 | 1,50E+07 | 1,50E+07 | overdominance | AX-586070824 | GO:0000786 | Cellular Component:nucleosome | NA | NA | IPR031059 | High mobility group protein HMGA, plant |
|  |  | Pd03 | 1,50E+07 | 1,50E+07 | overdominance | AX-586070824 | GO:0000786 | Cellular Component:nucleosome | NA | NA | IPR017956 | AT hook, DNA-binding motif |
|  |  | Pd03 | 1,50E+07 | 1,50E+07 | overdominance | AX-586070824 | GO:0000786 | Cellular Component:nucleosome | NA | NA | IPR005818 | Linker histone H1/H5, domain H15 |
|  |  | Pd03 | 1,50E+07 | 1,50E+07 | overdominance | AX-586070824 | GO:0006334 | Biological Process:nucleosome assembly | NA | NA | IPR036390 | Winged helix DNA-binding domain superfamily |
|  |  | Pd03 | 1,50E+07 | 1,50E+07 | overdominance | AX-586070824 | GO:0006334 | Biological Process:nucleosome assembly | NA | NA | IPR036388 | Winged helix-like DNA-binding domain superfamily |
|  |  | Pd03 | 1,50E+07 | 1,50E+07 | overdominance | AX-586070824 | GO:0006334 | Biological Process:nucleosome assembly | NA | NA | IPR000116 | High mobility group protein HMGA |
|  |  | Pd03 | 1,50E+07 | 1,50E+07 | overdominance | AX-586070824 | GO:0006334 | Biological Process:nucleosome assembly | NA | NA | IPR031059 | High mobility group protein HMGA, plant |
|  |  | Pd03 | 1,50E+07 | 1,50E+07 | overdominance | AX-586070824 | GO:0006334 | Biological Process:nucleosome assembly | NA | NA | IPR017956 | AT hook, DNA-binding motif |
|  |  | Pd03 | 1,50E+07 | 1,50E+07 | overdominance | AX-586070824 | GO:0006334 | Biological Process:nucleosome assembly | NA | NA | IPR005818 | Linker histone H1/H5, domain H15 |
|  |  | Pd03 | 1,50E+07 | 1,50E+07 | overdominance | AX-586070824 | GO:0006355 | Biological Process:regulation of transcription, DNA-templated | NA | NA | IPR036390 | Winged helix DNA-binding domain superfamily |
|  |  | Pd03 | 1,50E+07 | 1,50E+07 | overdominance | AX-586070824 | GO:0006355 | Biological Process:regulation of transcription, DNA-templated | NA | NA | IPR036388 | Winged helix-like DNA-binding domain superfamily |
|  |  | Pd03 | 1,50E+07 | 1,50E+07 | overdominance | AX-586070824 | GO:0006355 | Biological Process:regulation of transcription, DNA-templated | NA | NA | IPR000116 | High mobility group protein HMGA |
|  |  | Pd03 | 1,50E+07 | 1,50E+07 | overdominance | AX-586070824 | GO:0006355 | Biological Process:regulation of transcription, DNA-templated | NA | NA | IPR031059 | High mobility group protein HMGA, plant |
|  |  | Pd03 | 1,50E+07 | 1,50E+07 | overdominance | AX-586070824 | GO:0006355 | Biological Process:regulation of transcription, DNA-templated | NA | NA | IPR017956 | AT hook, DNA-binding motif |
|  |  | Pd03 | 1,50E+07 | 1,50E+07 | overdominance | AX-586070824 | GO:0006355 | Biological Process:regulation of transcription, DNA-templated | NA | NA | IPR005818 | Linker histone H1/H5, domain H15 |
|  |  | Pd03 | 1,50E+07 | 1,50E+07 | overdominance | AX-586070824 | GO:0005634 | Cellular Component:nucleus | NA | NA | IPR036390 | Winged helix DNA-binding domain superfamily |
|  |  | Pd03 | 1,50E+07 | 1,50E+07 | overdominance | AX-586070824 | GO:0005634 | Cellular Component:nucleus | NA | NA | IPR036388 | Winged helix-like DNA-binding domain superfamily |
|  |  | Pd03 | 1,50E+07 | 1,50E+07 | overdominance | AX-586070824 | GO:0005634 | Cellular Component:nucleus | NA | NA | IPR000116 | High mobility group protein HMGA |
|  |  | Pd03 | 1,50E+07 | 1,50E+07 | overdominance | AX-586070824 | GO:0005634 | Cellular Component:nucleus | NA | NA | IPR031059 | High mobility group protein HMGA, plant |
|  |  | Pd03 | 1,50E+07 | 1,50E+07 | overdominance | AX-586070824 | GO:0005634 | Cellular Component:nucleus | NA | NA | IPR017956 | AT hook, DNA-binding motif |
|  |  | Pd03 | 1,50E+07 | 1,50E+07 | overdominance | AX-586070824 | GO:0005634 | Cellular Component:nucleus | NA | NA | IPR005818 | Linker histone H1/H5, domain H15 |
| **Prudul26A014445T1** |  | Pd03 | 1,50E+07 | 1,50E+07 | overdominance | AX-586070824 | GO:0017022 | Molecular Function:myosin binding | NA | NA | IPR039306 | Myosin-binding protein |
|  |  | Pd03 | 1,50E+07 | 1,50E+07 | overdominance | AX-586070824 | GO:0017022 | Molecular Function:myosin binding | NA | NA | IPR007656 | GTD-binding domain |
| **Prudul26A002467T1** |  | Pd03 | 1,50E+07 | 1,50E+07 | overdominance | AX-586070824 | NA | NA | NA | NA | NA | NA |
| **Prudul26A020197T1** |  | Pd08 | 5081439 | 5083220 | overdominance | AX-586143715 | GO:0005515 | Molecular Function:protein binding | NA | NA | IPR001611 | Leucine-rich repeat |
|  |  | Pd08 | 5081439 | 5083220 | overdominance | AX-586143715 | GO:0005515 | Molecular Function:protein binding | NA | NA | IPR032675 | Leucine-rich repeat domain superfamily |
| **Prudul26A009300T1** |  | Pd08 | 5086673 | 5088502 | overdominance | AX-586143715 | GO:0005515 | Molecular Function:protein binding | NA | NA | IPR032675 | Leucine-rich repeat domain superfamily |
|  |  | Pd08 | 5086673 | 5088502 | overdominance | AX-586143715 | GO:0005515 | Molecular Function:protein binding | NA | NA | IPR001611 | Leucine-rich repeat |
| **Prudul26A001977T1** |  | Pd08 | 5092853 | 5096312 | overdominance | AX-586143715 | GO:0016788 | Molecular Function:hydrolase activity, acting on ester bonds | NA | NA | IPR036514 | SGNH hydrolase superfamily |
|  |  | Pd08 | 5092853 | 5096312 | overdominance | AX-586143715 | GO:0016788 | Molecular Function:hydrolase activity, acting on ester bonds | NA | NA | IPR001087 | GDSL lipase/esterase |
| **Prudul26A008726T1** |  | Pd08 | 5101968 | 5104868 | overdominance | AX-586143715 | GO:0005524 | Molecular Function:ATP binding | NA | NA | IPR000858 | S-locus glycoprotein domain |
|  |  | Pd08 | 5101968 | 5104868 | overdominance | AX-586143715 | GO:0005524 | Molecular Function:ATP binding | NA | NA | IPR000719 | Protein kinase domain |
|  |  | Pd08 | 5101968 | 5104868 | overdominance | AX-586143715 | GO:0005524 | Molecular Function:ATP binding | NA | NA | IPR017441 | Protein kinase, ATP binding site |
|  |  | Pd08 | 5101968 | 5104868 | overdominance | AX-586143715 | GO:0005524 | Molecular Function:ATP binding | NA | NA | IPR024171 | S-receptor-like serine/threonine-protein kinase |
|  |  | Pd08 | 5101968 | 5104868 | overdominance | AX-586143715 | GO:0005524 | Molecular Function:ATP binding | NA | NA | IPR001480 | Bulb-type lectin domain |
|  |  | Pd08 | 5101968 | 5104868 | overdominance | AX-586143715 | GO:0005524 | Molecular Function:ATP binding | NA | NA | IPR036426 | Bulb-type lectin domain superfamily |
|  |  | Pd08 | 5101968 | 5104868 | overdominance | AX-586143715 | GO:0005524 | Molecular Function:ATP binding | NA | NA | IPR011009 | Protein kinase-like domain superfamily |
|  |  | Pd08 | 5101968 | 5104868 | overdominance | AX-586143715 | GO:0005524 | Molecular Function:ATP binding | NA | NA | IPR008271 | Serine/threonine-protein kinase, active site |
|  |  | Pd08 | 5101968 | 5104868 | overdominance | AX-586143715 | GO:0048544 | Biological Process:recognition of pollen | NA | NA | IPR000858 | S-locus glycoprotein domain |
|  |  | Pd08 | 5101968 | 5104868 | overdominance | AX-586143715 | GO:0048544 | Biological Process:recognition of pollen | NA | NA | IPR000719 | Protein kinase domain |
|  |  | Pd08 | 5101968 | 5104868 | overdominance | AX-586143715 | GO:0048544 | Biological Process:recognition of pollen | NA | NA | IPR017441 | Protein kinase, ATP binding site |
|  |  | Pd08 | 5101968 | 5104868 | overdominance | AX-586143715 | GO:0048544 | Biological Process:recognition of pollen | NA | NA | IPR024171 | S-receptor-like serine/threonine-protein kinase |
|  |  | Pd08 | 5101968 | 5104868 | overdominance | AX-586143715 | GO:0048544 | Biological Process:recognition of pollen | NA | NA | IPR001480 | Bulb-type lectin domain |
|  |  | Pd08 | 5101968 | 5104868 | overdominance | AX-586143715 | GO:0048544 | Biological Process:recognition of pollen | NA | NA | IPR036426 | Bulb-type lectin domain superfamily |
|  |  | Pd08 | 5101968 | 5104868 | overdominance | AX-586143715 | GO:0048544 | Biological Process:recognition of pollen | NA | NA | IPR011009 | Protein kinase-like domain superfamily |
|  |  | Pd08 | 5101968 | 5104868 | overdominance | AX-586143715 | GO:0048544 | Biological Process:recognition of pollen | NA | NA | IPR008271 | Serine/threonine-protein kinase, active site |
|  |  | Pd08 | 5101968 | 5104868 | overdominance | AX-586143715 | GO:0006468 | Biological Process:protein phosphorylation | NA | NA | IPR000858 | S-locus glycoprotein domain |
|  |  | Pd08 | 5101968 | 5104868 | overdominance | AX-586143715 | GO:0006468 | Biological Process:protein phosphorylation | NA | NA | IPR000719 | Protein kinase domain |
|  |  | Pd08 | 5101968 | 5104868 | overdominance | AX-586143715 | GO:0006468 | Biological Process:protein phosphorylation | NA | NA | IPR017441 | Protein kinase, ATP binding site |
|  |  | Pd08 | 5101968 | 5104868 | overdominance | AX-586143715 | GO:0006468 | Biological Process:protein phosphorylation | NA | NA | IPR024171 | S-receptor-like serine/threonine-protein kinase |
|  |  | Pd08 | 5101968 | 5104868 | overdominance | AX-586143715 | GO:0006468 | Biological Process:protein phosphorylation | NA | NA | IPR001480 | Bulb-type lectin domain |
|  |  | Pd08 | 5101968 | 5104868 | overdominance | AX-586143715 | GO:0006468 | Biological Process:protein phosphorylation | NA | NA | IPR036426 | Bulb-type lectin domain superfamily |
|  |  | Pd08 | 5101968 | 5104868 | overdominance | AX-586143715 | GO:0006468 | Biological Process:protein phosphorylation | NA | NA | IPR011009 | Protein kinase-like domain superfamily |
|  |  | Pd08 | 5101968 | 5104868 | overdominance | AX-586143715 | GO:0006468 | Biological Process:protein phosphorylation | NA | NA | IPR008271 | Serine/threonine-protein kinase, active site |
|  |  | Pd08 | 5101968 | 5104868 | overdominance | AX-586143715 | GO:0004672 | Molecular Function:protein kinase activity | NA | NA | IPR000858 | S-locus glycoprotein domain |
|  |  | Pd08 | 5101968 | 5104868 | overdominance | AX-586143715 | GO:0004672 | Molecular Function:protein kinase activity | NA | NA | IPR000719 | Protein kinase domain |
|  |  | Pd08 | 5101968 | 5104868 | overdominance | AX-586143715 | GO:0004672 | Molecular Function:protein kinase activity | NA | NA | IPR017441 | Protein kinase, ATP binding site |
|  |  | Pd08 | 5101968 | 5104868 | overdominance | AX-586143715 | GO:0004672 | Molecular Function:protein kinase activity | NA | NA | IPR024171 | S-receptor-like serine/threonine-protein kinase |
|  |  | Pd08 | 5101968 | 5104868 | overdominance | AX-586143715 | GO:0004672 | Molecular Function:protein kinase activity | NA | NA | IPR001480 | Bulb-type lectin domain |
|  |  | Pd08 | 5101968 | 5104868 | overdominance | AX-586143715 | GO:0004672 | Molecular Function:protein kinase activity | NA | NA | IPR036426 | Bulb-type lectin domain superfamily |
|  |  | Pd08 | 5101968 | 5104868 | overdominance | AX-586143715 | GO:0004672 | Molecular Function:protein kinase activity | NA | NA | IPR011009 | Protein kinase-like domain superfamily |
|  |  | Pd08 | 5101968 | 5104868 | overdominance | AX-586143715 | GO:0004672 | Molecular Function:protein kinase activity | NA | NA | IPR008271 | Serine/threonine-protein kinase, active site |
|  |  | Pd08 | 5101968 | 5104868 | overdominance | AX-586143715 | GO:0004674 | Molecular Function:protein serine/threonine kinase activity | NA | NA | IPR000858 | S-locus glycoprotein domain |
|  |  | Pd08 | 5101968 | 5104868 | overdominance | AX-586143715 | GO:0004674 | Molecular Function:protein serine/threonine kinase activity | NA | NA | IPR000719 | Protein kinase domain |
|  |  | Pd08 | 5101968 | 5104868 | overdominance | AX-586143715 | GO:0004674 | Molecular Function:protein serine/threonine kinase activity | NA | NA | IPR017441 | Protein kinase, ATP binding site |
|  |  | Pd08 | 5101968 | 5104868 | overdominance | AX-586143715 | GO:0004674 | Molecular Function:protein serine/threonine kinase activity | NA | NA | IPR024171 | S-receptor-like serine/threonine-protein kinase |
|  |  | Pd08 | 5101968 | 5104868 | overdominance | AX-586143715 | GO:0004674 | Molecular Function:protein serine/threonine kinase activity | NA | NA | IPR001480 | Bulb-type lectin domain |
|  |  | Pd08 | 5101968 | 5104868 | overdominance | AX-586143715 | GO:0004674 | Molecular Function:protein serine/threonine kinase activity | NA | NA | IPR036426 | Bulb-type lectin domain superfamily |
|  |  | Pd08 | 5101968 | 5104868 | overdominance | AX-586143715 | GO:0004674 | Molecular Function:protein serine/threonine kinase activity | NA | NA | IPR011009 | Protein kinase-like domain superfamily |
|  |  | Pd08 | 5101968 | 5104868 | overdominance | AX-586143715 | GO:0004674 | Molecular Function:protein serine/threonine kinase activity | NA | NA | IPR008271 | Serine/threonine-protein kinase, active site |
| **Prudul26A001290T1** | Upstream gene variant | Pd08 | 5128652 | 5131764 | overdominance | AX-586143715 | GO:0006855 | Biological Process:drug transmembrane transport | NA | NA | IPR002528 | Multi antimicrobial extrusion protein |
|  |  | Pd08 | 5128652 | 5131764 | overdominance | AX-586143715 | GO:0015238 | Molecular Function:drug transmembrane transporter activity | NA | NA | IPR002528 | Multi antimicrobial extrusion protein |
|  |  | Pd08 | 5128652 | 5131764 | overdominance | AX-586143715 | GO:0016020 | Cellular Component:membrane | NA | NA | IPR002528 | Multi antimicrobial extrusion protein |
|  |  | Pd08 | 5128652 | 5131764 | overdominance | AX-586143715 | GO:0015297 | Molecular Function:antiporter activity | NA | NA | IPR002528 | Multi antimicrobial extrusion protein |
|  |  | Pd08 | 5128652 | 5131764 | overdominance | AX-586143715 | GO:0055085 | Biological Process:transmembrane transport | NA | NA | IPR002528 | Multi antimicrobial extrusion protein |
| **Prudul26A031640T1** |  | Pd08 | 5137252 | 5139296 | overdominance | AX-586143715 | NA | NA | NA | NA | IPR021099 | Plant organelle RNA recognition domain |
| **Prudul26A015954T1** |  | Pd08 | 5148744 | 5152011 | overdominance | AX-586143715 | GO:0006508 | Biological Process:proteolysis | NA | NA | IPR038765 | Papain-like cysteine peptidase superfamily |
|  |  | Pd08 | 5148744 | 5152011 | overdominance | AX-586143715 | GO:0006508 | Biological Process:proteolysis | NA | NA | IPR003653 | Ulp1 protease family, C-terminal catalytic domain |
|  |  | Pd08 | 5148744 | 5152011 | overdominance | AX-586143715 | GO:0006508 | Biological Process:proteolysis | NA | NA | IPR004252 | Probable transposase, Ptta/En/Spm, plant |
|  |  | Pd08 | 5148744 | 5152011 | overdominance | AX-586143715 | GO:0008234 | Molecular Function:cysteine-type peptidase activity | NA | NA | IPR038765 | Papain-like cysteine peptidase superfamily |
|  |  | Pd08 | 5148744 | 5152011 | overdominance | AX-586143715 | GO:0008234 | Molecular Function:cysteine-type peptidase activity | NA | NA | IPR003653 | Ulp1 protease family, C-terminal catalytic domain |
|  |  | Pd08 | 5148744 | 5152011 | overdominance | AX-586143715 | GO:0008234 | Molecular Function:cysteine-type peptidase activity | NA | NA | IPR004252 | Probable transposase, Ptta/En/Spm, plant |
| **Prudul26A015497T1** |  | Pd08 | 5170421 | 5170582 | overdominance | AX-586143715 | NA | NA | NA | NA | NA | NA |
| **Prudul26A017554T1** |  | Pd08 | 5171134 | 5171288 | overdominance | AX-586143715 | NA | NA | NA | NA | NA | NA |
| **Prudul26A020818T1** |  | Pd08 | 5174933 | 5175817 | overdominance | AX-586143715 | NA | NA | NA | NA | NA | NA |
| **Prudul26A030034T1** |  | Pd08 | 5176812 | 5183322 | overdominance | AX-586143715 | GO:0016791 | Molecular Function:phosphatase activity | K18039 | tyrosine-protein phosphatase non-receptor type 20 [EC:3.1.3.48] | IPR016130 | Protein-tyrosine phosphatase, active site |
|  |  | Pd08 | 5176812 | 5183322 | overdominance | AX-586143715 | GO:0016791 | Molecular Function:phosphatase activity | K18039 | tyrosine-protein phosphatase non-receptor type 20 [EC:3.1.3.48] | IPR029021 | Protein-tyrosine phosphatase-like |
|  |  | Pd08 | 5176812 | 5183322 | overdominance | AX-586143715 | GO:0016791 | Molecular Function:phosphatase activity | K18039 | tyrosine-protein phosphatase non-receptor type 20 [EC:3.1.3.48] | IPR000242 | PTP type protein phosphatase |
|  |  | Pd08 | 5176812 | 5183322 | overdominance | AX-586143715 | GO:0016791 | Molecular Function:phosphatase activity | K18039 | tyrosine-protein phosphatase non-receptor type 20 [EC:3.1.3.48] | IPR000387 | Tyrosine specific protein phosphatases domain |
|  |  | Pd08 | 5176812 | 5183322 | overdominance | AX-586143715 | GO:0016311 | Biological Process:dephosphorylation | K18039 | tyrosine-protein phosphatase non-receptor type 20 [EC:3.1.3.48] | IPR016130 | Protein-tyrosine phosphatase, active site |
|  |  | Pd08 | 5176812 | 5183322 | overdominance | AX-586143715 | GO:0016311 | Biological Process:dephosphorylation | K18039 | tyrosine-protein phosphatase non-receptor type 20 [EC:3.1.3.48] | IPR029021 | Protein-tyrosine phosphatase-like |
|  |  | Pd08 | 5176812 | 5183322 | overdominance | AX-586143715 | GO:0016311 | Biological Process:dephosphorylation | K18039 | tyrosine-protein phosphatase non-receptor type 20 [EC:3.1.3.48] | IPR000242 | PTP type protein phosphatase |
|  |  | Pd08 | 5176812 | 5183322 | overdominance | AX-586143715 | GO:0016311 | Biological Process:dephosphorylation | K18039 | tyrosine-protein phosphatase non-receptor type 20 [EC:3.1.3.48] | IPR000387 | Tyrosine specific protein phosphatases domain |
|  |  | Pd08 | 5176812 | 5183322 | overdominance | AX-586143715 | GO:0004725 | Molecular Function:protein tyrosine phosphatase activity | K18039 | tyrosine-protein phosphatase non-receptor type 20 [EC:3.1.3.48] | IPR016130 | Protein-tyrosine phosphatase, active site |
|  |  | Pd08 | 5176812 | 5183322 | overdominance | AX-586143715 | GO:0004725 | Molecular Function:protein tyrosine phosphatase activity | K18039 | tyrosine-protein phosphatase non-receptor type 20 [EC:3.1.3.48] | IPR029021 | Protein-tyrosine phosphatase-like |
|  |  | Pd08 | 5176812 | 5183322 | overdominance | AX-586143715 | GO:0004725 | Molecular Function:protein tyrosine phosphatase activity | K18039 | tyrosine-protein phosphatase non-receptor type 20 [EC:3.1.3.48] | IPR000242 | PTP type protein phosphatase |
|  |  | Pd08 | 5176812 | 5183322 | overdominance | AX-586143715 | GO:0004725 | Molecular Function:protein tyrosine phosphatase activity | K18039 | tyrosine-protein phosphatase non-receptor type 20 [EC:3.1.3.48] | IPR000387 | Tyrosine specific protein phosphatases domain |
|  |  | Pd08 | 5176812 | 5183322 | overdominance | AX-586143715 | GO:0006470 | Biological Process:protein dephosphorylation | K18039 | tyrosine-protein phosphatase non-receptor type 20 [EC:3.1.3.48] | IPR016130 | Protein-tyrosine phosphatase, active site |
|  |  | Pd08 | 5176812 | 5183322 | overdominance | AX-586143715 | GO:0006470 | Biological Process:protein dephosphorylation | K18039 | tyrosine-protein phosphatase non-receptor type 20 [EC:3.1.3.48] | IPR029021 | Protein-tyrosine phosphatase-like |
|  |  | Pd08 | 5176812 | 5183322 | overdominance | AX-586143715 | GO:0006470 | Biological Process:protein dephosphorylation | K18039 | tyrosine-protein phosphatase non-receptor type 20 [EC:3.1.3.48] | IPR000242 | PTP type protein phosphatase |
|  |  | Pd08 | 5176812 | 5183322 | overdominance | AX-586143715 | GO:0006470 | Biological Process:protein dephosphorylation | K18039 | tyrosine-protein phosphatase non-receptor type 20 [EC:3.1.3.48] | IPR000387 | Tyrosine specific protein phosphatases domain |
| **Prudul26A030034T2** |  | Pd08 | 5176812 | 5183322 | overdominance | AX-586143715 | GO:0004725 | Molecular Function:protein tyrosine phosphatase activity | K18039 | tyrosine-protein phosphatase non-receptor type 20 [EC:3.1.3.48] | IPR000387 | Tyrosine specific protein phosphatases domain |
|  |  | Pd08 | 5176812 | 5183322 | overdominance | AX-586143715 | GO:0004725 | Molecular Function:protein tyrosine phosphatase activity | K18039 | tyrosine-protein phosphatase non-receptor type 20 [EC:3.1.3.48] | IPR000242 | PTP type protein phosphatase |
|  |  | Pd08 | 5176812 | 5183322 | overdominance | AX-586143715 | GO:0004725 | Molecular Function:protein tyrosine phosphatase activity | K18039 | tyrosine-protein phosphatase non-receptor type 20 [EC:3.1.3.48] | IPR029021 | Protein-tyrosine phosphatase-like |
|  |  | Pd08 | 5176812 | 5183322 | overdominance | AX-586143715 | GO:0004725 | Molecular Function:protein tyrosine phosphatase activity | K18039 | tyrosine-protein phosphatase non-receptor type 20 [EC:3.1.3.48] | IPR016130 | Protein-tyrosine phosphatase, active site |
|  |  | Pd08 | 5176812 | 5183322 | overdominance | AX-586143715 | GO:0016311 | Biological Process:dephosphorylation | K18039 | tyrosine-protein phosphatase non-receptor type 20 [EC:3.1.3.48] | IPR000387 | Tyrosine specific protein phosphatases domain |
|  |  | Pd08 | 5176812 | 5183322 | overdominance | AX-586143715 | GO:0016311 | Biological Process:dephosphorylation | K18039 | tyrosine-protein phosphatase non-receptor type 20 [EC:3.1.3.48] | IPR000242 | PTP type protein phosphatase |
|  |  | Pd08 | 5176812 | 5183322 | overdominance | AX-586143715 | GO:0016311 | Biological Process:dephosphorylation | K18039 | tyrosine-protein phosphatase non-receptor type 20 [EC:3.1.3.48] | IPR029021 | Protein-tyrosine phosphatase-like |
|  |  | Pd08 | 5176812 | 5183322 | overdominance | AX-586143715 | GO:0016311 | Biological Process:dephosphorylation | K18039 | tyrosine-protein phosphatase non-receptor type 20 [EC:3.1.3.48] | IPR016130 | Protein-tyrosine phosphatase, active site |
|  |  | Pd08 | 5176812 | 5183322 | overdominance | AX-586143715 | GO:0006470 | Biological Process:protein dephosphorylation | K18039 | tyrosine-protein phosphatase non-receptor type 20 [EC:3.1.3.48] | IPR000387 | Tyrosine specific protein phosphatases domain |
|  |  | Pd08 | 5176812 | 5183322 | overdominance | AX-586143715 | GO:0006470 | Biological Process:protein dephosphorylation | K18039 | tyrosine-protein phosphatase non-receptor type 20 [EC:3.1.3.48] | IPR000242 | PTP type protein phosphatase |
|  |  | Pd08 | 5176812 | 5183322 | overdominance | AX-586143715 | GO:0006470 | Biological Process:protein dephosphorylation | K18039 | tyrosine-protein phosphatase non-receptor type 20 [EC:3.1.3.48] | IPR029021 | Protein-tyrosine phosphatase-like |
|  |  | Pd08 | 5176812 | 5183322 | overdominance | AX-586143715 | GO:0006470 | Biological Process:protein dephosphorylation | K18039 | tyrosine-protein phosphatase non-receptor type 20 [EC:3.1.3.48] | IPR016130 | Protein-tyrosine phosphatase, active site |
|  |  | Pd08 | 5176812 | 5183322 | overdominance | AX-586143715 | GO:0016791 | Molecular Function:phosphatase activity | K18039 | tyrosine-protein phosphatase non-receptor type 20 [EC:3.1.3.48] | IPR000387 | Tyrosine specific protein phosphatases domain |
|  |  | Pd08 | 5176812 | 5183322 | overdominance | AX-586143715 | GO:0016791 | Molecular Function:phosphatase activity | K18039 | tyrosine-protein phosphatase non-receptor type 20 [EC:3.1.3.48] | IPR000242 | PTP type protein phosphatase |
|  |  | Pd08 | 5176812 | 5183322 | overdominance | AX-586143715 | GO:0016791 | Molecular Function:phosphatase activity | K18039 | tyrosine-protein phosphatase non-receptor type 20 [EC:3.1.3.48] | IPR029021 | Protein-tyrosine phosphatase-like |
|  |  | Pd08 | 5176812 | 5183322 | overdominance | AX-586143715 | GO:0016791 | Molecular Function:phosphatase activity | K18039 | tyrosine-protein phosphatase non-receptor type 20 [EC:3.1.3.48] | IPR016130 | Protein-tyrosine phosphatase, active site |
| **Prudul26A011967T1** |  | Pd08 | 9110630 | 9114282 | overdominance | AX-586146007 | GO:0006364 | Biological Process:rRNA processing | NA | NA | IPR023799 | Ribosome-binding factor A domain superfamily |
|  |  | Pd08 | 9110630 | 9114282 | overdominance | AX-586146007 | GO:0006364 | Biological Process:rRNA processing | NA | NA | IPR000238 | Ribosome-binding factor A |
|  |  | Pd08 | 9110630 | 9114282 | overdominance | AX-586146007 | GO:0006364 | Biological Process:rRNA processing | NA | NA | IPR020053 | Ribosome-binding factor A, conserved site |
|  |  | Pd08 | 9110630 | 9114282 | overdominance | AX-586146007 | GO:0006364 | Biological Process:rRNA processing | NA | NA | IPR015946 | K homology domain-like, alpha/beta |
| **Prudul26A019249T1** |  | Pd08 | 9115166 | 9117723 | overdominance | AX-586146007 | NA | NA | NA | NA | NA | NA |
| **Prudul26A002402T1** |  | Pd08 | 9156410 | 9156700 | overdominance | AX-586146007 | NA | NA | NA | NA | NA | NA |
| **Prudul26A008988T1** | Upstream gene variant | Pd08 | 9163275 | 9167170 | overdominance | AX-586146007 | NA | NA | NA | NA | IPR010409 | GAGA-binding transcriptional activator |
| **Prudul26A008988T2** |  | Pd08 | 9163275 | 9167170 | overdominance | AX-586146007 | NA | NA | NA | NA | IPR010409 | GAGA-binding transcriptional activator |
| **Prudul26A016539T1** | Intron variant | Pd08 | 9167981 | 9172688 | overdominance | AX-586146007 | GO:0006511 | Biological Process:ubiquitin-dependent protein catabolic process | NA | NA | IPR004854 | Ubiquitin fusion degradation protein Ufd1-like |
| **Prudul26A016539T2** |  | Pd08 | 9167981 | 9172688 | overdominance | AX-586146007 | GO:0006511 | Biological Process:ubiquitin-dependent protein catabolic process | NA | NA | IPR004854 | Ubiquitin fusion degradation protein Ufd1-like |
| **Prudul26A016539T3** |  | Pd08 | 9167981 | 9172688 | overdominance | AX-586146007 | GO:0006511 | Biological Process:ubiquitin-dependent protein catabolic process | NA | NA | IPR004854 | Ubiquitin fusion degradation protein Ufd1-like |
| **Prudul26A026295T1** |  | Pd08 | 9178215 | 9181311 | overdominance | AX-586146007 | GO:0008792 | Molecular Function:arginine decarboxylase activity | K01583 | arginine decarboxylase [EC:4.1.1.19] | IPR000183 | Ornithine/DAP/Arg decarboxylase |
|  |  | Pd08 | 9178215 | 9181311 | overdominance | AX-586146007 | GO:0008792 | Molecular Function:arginine decarboxylase activity | K01583 | arginine decarboxylase [EC:4.1.1.19] | IPR022653 | Orn/DAP/Arg decarboxylase 2, pyridoxal-phosphate binding site |
|  |  | Pd08 | 9178215 | 9181311 | overdominance | AX-586146007 | GO:0008792 | Molecular Function:arginine decarboxylase activity | K01583 | arginine decarboxylase [EC:4.1.1.19] | IPR022657 | Orn/DAP/Arg decarboxylase 2, conserved site |
|  |  | Pd08 | 9178215 | 9181311 | overdominance | AX-586146007 | GO:0008792 | Molecular Function:arginine decarboxylase activity | K01583 | arginine decarboxylase [EC:4.1.1.19] | IPR029066 | PLP-binding barrel |
|  |  | Pd08 | 9178215 | 9181311 | overdominance | AX-586146007 | GO:0008792 | Molecular Function:arginine decarboxylase activity | K01583 | arginine decarboxylase [EC:4.1.1.19] | IPR022644 | Orn/DAP/Arg decarboxylase 2, N-terminal |
|  |  | Pd08 | 9178215 | 9181311 | overdominance | AX-586146007 | GO:0008792 | Molecular Function:arginine decarboxylase activity | K01583 | arginine decarboxylase [EC:4.1.1.19] | IPR009006 | Alanine racemase/group IV decarboxylase, C-terminal |
|  |  | Pd08 | 9178215 | 9181311 | overdominance | AX-586146007 | GO:0008792 | Molecular Function:arginine decarboxylase activity | K01583 | arginine decarboxylase [EC:4.1.1.19] | IPR002985 | Arginine decarboxylase |
|  |  | Pd08 | 9178215 | 9181311 | overdominance | AX-586146007 | GO:0003824 | Molecular Function:catalytic activity | K01583 | arginine decarboxylase [EC:4.1.1.19] | IPR000183 | Ornithine/DAP/Arg decarboxylase |
|  |  | Pd08 | 9178215 | 9181311 | overdominance | AX-586146007 | GO:0003824 | Molecular Function:catalytic activity | K01583 | arginine decarboxylase [EC:4.1.1.19] | IPR022653 | Orn/DAP/Arg decarboxylase 2, pyridoxal-phosphate binding site |
|  |  | Pd08 | 9178215 | 9181311 | overdominance | AX-586146007 | GO:0003824 | Molecular Function:catalytic activity | K01583 | arginine decarboxylase [EC:4.1.1.19] | IPR022657 | Orn/DAP/Arg decarboxylase 2, conserved site |
|  |  | Pd08 | 9178215 | 9181311 | overdominance | AX-586146007 | GO:0003824 | Molecular Function:catalytic activity | K01583 | arginine decarboxylase [EC:4.1.1.19] | IPR029066 | PLP-binding barrel |
|  |  | Pd08 | 9178215 | 9181311 | overdominance | AX-586146007 | GO:0003824 | Molecular Function:catalytic activity | K01583 | arginine decarboxylase [EC:4.1.1.19] | IPR022644 | Orn/DAP/Arg decarboxylase 2, N-terminal |
|  |  | Pd08 | 9178215 | 9181311 | overdominance | AX-586146007 | GO:0003824 | Molecular Function:catalytic activity | K01583 | arginine decarboxylase [EC:4.1.1.19] | IPR009006 | Alanine racemase/group IV decarboxylase, C-terminal |
|  |  | Pd08 | 9178215 | 9181311 | overdominance | AX-586146007 | GO:0003824 | Molecular Function:catalytic activity | K01583 | arginine decarboxylase [EC:4.1.1.19] | IPR002985 | Arginine decarboxylase |
|  |  | Pd08 | 9178215 | 9181311 | overdominance | AX-586146007 | GO:0008295 | Biological Process:spermidine biosynthetic process | K01583 | arginine decarboxylase [EC:4.1.1.19] | IPR000183 | Ornithine/DAP/Arg decarboxylase |
|  |  | Pd08 | 9178215 | 9181311 | overdominance | AX-586146007 | GO:0008295 | Biological Process:spermidine biosynthetic process | K01583 | arginine decarboxylase [EC:4.1.1.19] | IPR022653 | Orn/DAP/Arg decarboxylase 2, pyridoxal-phosphate binding site |
|  |  | Pd08 | 9178215 | 9181311 | overdominance | AX-586146007 | GO:0008295 | Biological Process:spermidine biosynthetic process | K01583 | arginine decarboxylase [EC:4.1.1.19] | IPR022657 | Orn/DAP/Arg decarboxylase 2, conserved site |
|  |  | Pd08 | 9178215 | 9181311 | overdominance | AX-586146007 | GO:0008295 | Biological Process:spermidine biosynthetic process | K01583 | arginine decarboxylase [EC:4.1.1.19] | IPR029066 | PLP-binding barrel |
|  |  | Pd08 | 9178215 | 9181311 | overdominance | AX-586146007 | GO:0008295 | Biological Process:spermidine biosynthetic process | K01583 | arginine decarboxylase [EC:4.1.1.19] | IPR022644 | Orn/DAP/Arg decarboxylase 2, N-terminal |
|  |  | Pd08 | 9178215 | 9181311 | overdominance | AX-586146007 | GO:0008295 | Biological Process:spermidine biosynthetic process | K01583 | arginine decarboxylase [EC:4.1.1.19] | IPR009006 | Alanine racemase/group IV decarboxylase, C-terminal |
|  |  | Pd08 | 9178215 | 9181311 | overdominance | AX-586146007 | GO:0008295 | Biological Process:spermidine biosynthetic process | K01583 | arginine decarboxylase [EC:4.1.1.19] | IPR002985 | Arginine decarboxylase |
|  |  | Pd08 | 9178215 | 9181311 | overdominance | AX-586146007 | GO:0006527 | Biological Process:arginine catabolic process | K01583 | arginine decarboxylase [EC:4.1.1.19] | IPR000183 | Ornithine/DAP/Arg decarboxylase |
|  |  | Pd08 | 9178215 | 9181311 | overdominance | AX-586146007 | GO:0006527 | Biological Process:arginine catabolic process | K01583 | arginine decarboxylase [EC:4.1.1.19] | IPR022653 | Orn/DAP/Arg decarboxylase 2, pyridoxal-phosphate binding site |
|  |  | Pd08 | 9178215 | 9181311 | overdominance | AX-586146007 | GO:0006527 | Biological Process:arginine catabolic process | K01583 | arginine decarboxylase [EC:4.1.1.19] | IPR022657 | Orn/DAP/Arg decarboxylase 2, conserved site |
|  |  | Pd08 | 9178215 | 9181311 | overdominance | AX-586146007 | GO:0006527 | Biological Process:arginine catabolic process | K01583 | arginine decarboxylase [EC:4.1.1.19] | IPR029066 | PLP-binding barrel |
|  |  | Pd08 | 9178215 | 9181311 | overdominance | AX-586146007 | GO:0006527 | Biological Process:arginine catabolic process | K01583 | arginine decarboxylase [EC:4.1.1.19] | IPR022644 | Orn/DAP/Arg decarboxylase 2, N-terminal |
|  |  | Pd08 | 9178215 | 9181311 | overdominance | AX-586146007 | GO:0006527 | Biological Process:arginine catabolic process | K01583 | arginine decarboxylase [EC:4.1.1.19] | IPR009006 | Alanine racemase/group IV decarboxylase, C-terminal |
|  |  | Pd08 | 9178215 | 9181311 | overdominance | AX-586146007 | GO:0006527 | Biological Process:arginine catabolic process | K01583 | arginine decarboxylase [EC:4.1.1.19] | IPR002985 | Arginine decarboxylase |
| **Prudul26A007057T1** |  | Pd08 | 9202534 | 9205615 | overdominance | AX-586146007 | GO:0033179 | Cellular Component:proton-transporting V-type ATPase, V0 domain | NA | NA | IPR011555 | V-ATPase proteolipid subunit C, eukaryotic |
|  |  | Pd08 | 9202534 | 9205615 | overdominance | AX-586146007 | GO:0033179 | Cellular Component:proton-transporting V-type ATPase, V0 domain | NA | NA | IPR035921 | F/V-ATP synthase subunit C superfamily |
|  |  | Pd08 | 9202534 | 9205615 | overdominance | AX-586146007 | GO:0033179 | Cellular Component:proton-transporting V-type ATPase, V0 domain | NA | NA | IPR000245 | V-ATPase proteolipid subunit |
|  |  | Pd08 | 9202534 | 9205615 | overdominance | AX-586146007 | GO:0033179 | Cellular Component:proton-transporting V-type ATPase, V0 domain | NA | NA | IPR002379 | V-ATPase proteolipid subunit C-like domain |
|  |  | Pd08 | 9202534 | 9205615 | overdominance | AX-586146007 | GO:0033177 | Cellular Component:proton-transporting two-sector ATPase complex, proton-transporting domain | NA | NA | IPR011555 | V-ATPase proteolipid subunit C, eukaryotic |
|  |  | Pd08 | 9202534 | 9205615 | overdominance | AX-586146007 | GO:0033177 | Cellular Component:proton-transporting two-sector ATPase complex, proton-transporting domain | NA | NA | IPR035921 | F/V-ATP synthase subunit C superfamily |
|  |  | Pd08 | 9202534 | 9205615 | overdominance | AX-586146007 | GO:0033177 | Cellular Component:proton-transporting two-sector ATPase complex, proton-transporting domain | NA | NA | IPR000245 | V-ATPase proteolipid subunit |
|  |  | Pd08 | 9202534 | 9205615 | overdominance | AX-586146007 | GO:0033177 | Cellular Component:proton-transporting two-sector ATPase complex, proton-transporting domain | NA | NA | IPR002379 | V-ATPase proteolipid subunit C-like domain |
|  |  | Pd08 | 9202534 | 9205615 | overdominance | AX-586146007 | GO:0015078 | Molecular Function:proton transmembrane transporter activity | NA | NA | IPR011555 | V-ATPase proteolipid subunit C, eukaryotic |
|  |  | Pd08 | 9202534 | 9205615 | overdominance | AX-586146007 | GO:0015078 | Molecular Function:proton transmembrane transporter activity | NA | NA | IPR035921 | F/V-ATP synthase subunit C superfamily |
|  |  | Pd08 | 9202534 | 9205615 | overdominance | AX-586146007 | GO:0015078 | Molecular Function:proton transmembrane transporter activity | NA | NA | IPR000245 | V-ATPase proteolipid subunit |
|  |  | Pd08 | 9202534 | 9205615 | overdominance | AX-586146007 | GO:0015078 | Molecular Function:proton transmembrane transporter activity | NA | NA | IPR002379 | V-ATPase proteolipid subunit C-like domain |
|  |  | Pd08 | 9202534 | 9205615 | overdominance | AX-586146007 | GO:0015991 | Biological Process:ATP hydrolysis coupled proton transport | NA | NA | IPR011555 | V-ATPase proteolipid subunit C, eukaryotic |
|  |  | Pd08 | 9202534 | 9205615 | overdominance | AX-586146007 | GO:0015991 | Biological Process:ATP hydrolysis coupled proton transport | NA | NA | IPR035921 | F/V-ATP synthase subunit C superfamily |
|  |  | Pd08 | 9202534 | 9205615 | overdominance | AX-586146007 | GO:0015991 | Biological Process:ATP hydrolysis coupled proton transport | NA | NA | IPR000245 | V-ATPase proteolipid subunit |
|  |  | Pd08 | 9202534 | 9205615 | overdominance | AX-586146007 | GO:0015991 | Biological Process:ATP hydrolysis coupled proton transport | NA | NA | IPR002379 | V-ATPase proteolipid subunit C-like domain |
| **Prudul26A015648T1** |  | Pd08 | 9209882 | 9217786 | overdominance | AX-586146007 | GO:0016491 | Molecular Function:oxidoreductase activity | K00011 | aldehyde reductase [EC:1.1.1.21] | IPR036812 | NADP-dependent oxidoreductase domain superfamily |
|  |  | Pd08 | 9209882 | 9217786 | overdominance | AX-586146007 | GO:0016491 | Molecular Function:oxidoreductase activity | K00011 | aldehyde reductase [EC:1.1.1.21] | IPR018170 | Aldo/keto reductase, conserved site |
|  |  | Pd08 | 9209882 | 9217786 | overdominance | AX-586146007 | GO:0016491 | Molecular Function:oxidoreductase activity | K00011 | aldehyde reductase [EC:1.1.1.21] | IPR020471 | Aldo/keto reductase |
|  |  | Pd08 | 9209882 | 9217786 | overdominance | AX-586146007 | GO:0016491 | Molecular Function:oxidoreductase activity | K00011 | aldehyde reductase [EC:1.1.1.21] | IPR023210 | NADP-dependent oxidoreductase domain |
|  |  | Pd08 | 9209882 | 9217786 | overdominance | AX-586146007 | GO:0055114 | Biological Process:oxidation-reduction process | K00011 | aldehyde reductase [EC:1.1.1.21] | IPR036812 | NADP-dependent oxidoreductase domain superfamily |
|  |  | Pd08 | 9209882 | 9217786 | overdominance | AX-586146007 | GO:0055114 | Biological Process:oxidation-reduction process | K00011 | aldehyde reductase [EC:1.1.1.21] | IPR018170 | Aldo/keto reductase, conserved site |
|  |  | Pd08 | 9209882 | 9217786 | overdominance | AX-586146007 | GO:0055114 | Biological Process:oxidation-reduction process | K00011 | aldehyde reductase [EC:1.1.1.21] | IPR020471 | Aldo/keto reductase |
|  |  | Pd08 | 9209882 | 9217786 | overdominance | AX-586146007 | GO:0055114 | Biological Process:oxidation-reduction process | K00011 | aldehyde reductase [EC:1.1.1.21] | IPR023210 | NADP-dependent oxidoreductase domain |
| **Prudul26A021417T1** |  | Pd08 | 9216952 | 9220557 | overdominance | AX-586146007 | GO:0006629 | Biological Process:lipid metabolic process | K12345 | 3-oxo-5-alpha-steroid 4-dehydrogenase 3 [EC:1.3.1.22 1.3.1.94] | IPR039698 | Polyprenol reductase |
|  |  | Pd08 | 9216952 | 9220557 | overdominance | AX-586146007 | GO:0006629 | Biological Process:lipid metabolic process | K12345 | 3-oxo-5-alpha-steroid 4-dehydrogenase 3 [EC:1.3.1.22 1.3.1.94] | IPR001104 | 3-oxo-5-alpha-steroid 4-dehydrogenase, C-terminal |
|  |  | Pd08 | 9216952 | 9220557 | overdominance | AX-586146007 | GO:0006488 | Biological Process:dolichol-linked oligosaccharide biosynthetic process | K12345 | 3-oxo-5-alpha-steroid 4-dehydrogenase 3 [EC:1.3.1.22 1.3.1.94] | IPR039698 | Polyprenol reductase |
|  |  | Pd08 | 9216952 | 9220557 | overdominance | AX-586146007 | GO:0006488 | Biological Process:dolichol-linked oligosaccharide biosynthetic process | K12345 | 3-oxo-5-alpha-steroid 4-dehydrogenase 3 [EC:1.3.1.22 1.3.1.94] | IPR001104 | 3-oxo-5-alpha-steroid 4-dehydrogenase, C-terminal |
|  |  | Pd08 | 9216952 | 9220557 | overdominance | AX-586146007 | GO:0016627 | Molecular Function:oxidoreductase activity, acting on the CH-CH group of donors | K12345 | 3-oxo-5-alpha-steroid 4-dehydrogenase 3 [EC:1.3.1.22 1.3.1.94] | IPR039698 | Polyprenol reductase |
|  |  | Pd08 | 9216952 | 9220557 | overdominance | AX-586146007 | GO:0016627 | Molecular Function:oxidoreductase activity, acting on the CH-CH group of donors | K12345 | 3-oxo-5-alpha-steroid 4-dehydrogenase 3 [EC:1.3.1.22 1.3.1.94] | IPR001104 | 3-oxo-5-alpha-steroid 4-dehydrogenase, C-terminal |
|  |  | Pd08 | 9216952 | 9220557 | overdominance | AX-586146007 | GO:0005737 | Cellular Component:cytoplasm | K12345 | 3-oxo-5-alpha-steroid 4-dehydrogenase 3 [EC:1.3.1.22 1.3.1.94] | IPR039698 | Polyprenol reductase |
|  |  | Pd08 | 9216952 | 9220557 | overdominance | AX-586146007 | GO:0005737 | Cellular Component:cytoplasm | K12345 | 3-oxo-5-alpha-steroid 4-dehydrogenase 3 [EC:1.3.1.22 1.3.1.94] | IPR001104 | 3-oxo-5-alpha-steroid 4-dehydrogenase, C-terminal |
|  |  | Pd08 | 9216952 | 9220557 | overdominance | AX-586146007 | GO:0003865 | Molecular Function:3-oxo-5-alpha-steroid 4-dehydrogenase activity | K12345 | 3-oxo-5-alpha-steroid 4-dehydrogenase 3 [EC:1.3.1.22 1.3.1.94] | IPR039698 | Polyprenol reductase |
|  |  | Pd08 | 9216952 | 9220557 | overdominance | AX-586146007 | GO:0003865 | Molecular Function:3-oxo-5-alpha-steroid 4-dehydrogenase activity | K12345 | 3-oxo-5-alpha-steroid 4-dehydrogenase 3 [EC:1.3.1.22 1.3.1.94] | IPR001104 | 3-oxo-5-alpha-steroid 4-dehydrogenase, C-terminal |
|  |  | Pd08 | 9216952 | 9220557 | overdominance | AX-586146007 | GO:0016021 | Cellular Component:integral component of membrane | K12345 | 3-oxo-5-alpha-steroid 4-dehydrogenase 3 [EC:1.3.1.22 1.3.1.94] | IPR039698 | Polyprenol reductase |
|  |  | Pd08 | 9216952 | 9220557 | overdominance | AX-586146007 | GO:0016021 | Cellular Component:integral component of membrane | K12345 | 3-oxo-5-alpha-steroid 4-dehydrogenase 3 [EC:1.3.1.22 1.3.1.94] | IPR001104 | 3-oxo-5-alpha-steroid 4-dehydrogenase, C-terminal |
| **Prudul26A030455T1** |  | Pd08 | 9220391 | 9227768 | overdominance | AX-586146007 | GO:0003824 | Molecular Function:catalytic activity | NA | NA | IPR013549 | Domain of unknown function DUF1731 |
|  |  | Pd08 | 9220391 | 9227768 | overdominance | AX-586146007 | GO:0003824 | Molecular Function:catalytic activity | NA | NA | IPR001509 | NAD-dependent epimerase/dehydratase |
|  |  | Pd08 | 9220391 | 9227768 | overdominance | AX-586146007 | GO:0003824 | Molecular Function:catalytic activity | NA | NA | IPR010099 | Epimerase family protein SDR39U1 |
|  |  | Pd08 | 9220391 | 9227768 | overdominance | AX-586146007 | GO:0003824 | Molecular Function:catalytic activity | NA | NA | IPR036291 | NAD(P)-binding domain superfamily |
|  |  | Pd08 | 9220391 | 9227768 | overdominance | AX-586146007 | GO:0050662 | Molecular Function:coenzyme binding | NA | NA | IPR013549 | Domain of unknown function DUF1731 |
|  |  | Pd08 | 9220391 | 9227768 | overdominance | AX-586146007 | GO:0050662 | Molecular Function:coenzyme binding | NA | NA | IPR001509 | NAD-dependent epimerase/dehydratase |
|  |  | Pd08 | 9220391 | 9227768 | overdominance | AX-586146007 | GO:0050662 | Molecular Function:coenzyme binding | NA | NA | IPR010099 | Epimerase family protein SDR39U1 |
|  |  | Pd08 | 9220391 | 9227768 | overdominance | AX-586146007 | GO:0050662 | Molecular Function:coenzyme binding | NA | NA | IPR036291 | NAD(P)-binding domain superfamily |
| **Prudul26A030455T2** |  | Pd08 | 9221486 | 9227921 | overdominance | AX-586146007 | GO:0050662 | Molecular Function:coenzyme binding | NA | NA | IPR013549 | Domain of unknown function DUF1731 |
|  |  | Pd08 | 9221486 | 9227921 | overdominance | AX-586146007 | GO:0050662 | Molecular Function:coenzyme binding | NA | NA | IPR001509 | NAD-dependent epimerase/dehydratase |
|  |  | Pd08 | 9221486 | 9227921 | overdominance | AX-586146007 | GO:0050662 | Molecular Function:coenzyme binding | NA | NA | IPR010099 | Epimerase family protein SDR39U1 |
|  |  | Pd08 | 9221486 | 9227921 | overdominance | AX-586146007 | GO:0050662 | Molecular Function:coenzyme binding | NA | NA | IPR036291 | NAD(P)-binding domain superfamily |
|  |  | Pd08 | 9221486 | 9227921 | overdominance | AX-586146007 | GO:0003824 | Molecular Function:catalytic activity | NA | NA | IPR013549 | Domain of unknown function DUF1731 |
|  |  | Pd08 | 9221486 | 9227921 | overdominance | AX-586146007 | GO:0003824 | Molecular Function:catalytic activity | NA | NA | IPR001509 | NAD-dependent epimerase/dehydratase |
|  |  | Pd08 | 9221486 | 9227921 | overdominance | AX-586146007 | GO:0003824 | Molecular Function:catalytic activity | NA | NA | IPR010099 | Epimerase family protein SDR39U1 |
|  |  | Pd08 | 9221486 | 9227921 | overdominance | AX-586146007 | GO:0003824 | Molecular Function:catalytic activity | NA | NA | IPR036291 | NAD(P)-binding domain superfamily |
| **Prudul26A030455T3** |  | Pd08 | 9221736 | 9227921 | overdominance | AX-586146007 | GO:0003824 | Molecular Function:catalytic activity | NA | NA | IPR036291 | NAD(P)-binding domain superfamily |
|  |  | Pd08 | 9221736 | 9227921 | overdominance | AX-586146007 | GO:0003824 | Molecular Function:catalytic activity | NA | NA | IPR001509 | NAD-dependent epimerase/dehydratase |
|  |  | Pd08 | 9221736 | 9227921 | overdominance | AX-586146007 | GO:0003824 | Molecular Function:catalytic activity | NA | NA | IPR010099 | Epimerase family protein SDR39U1 |
|  |  | Pd08 | 9221736 | 9227921 | overdominance | AX-586146007 | GO:0003824 | Molecular Function:catalytic activity | NA | NA | IPR013549 | Domain of unknown function DUF1731 |
|  |  | Pd08 | 9221736 | 9227921 | overdominance | AX-586146007 | GO:0050662 | Molecular Function:coenzyme binding | NA | NA | IPR036291 | NAD(P)-binding domain superfamily |
|  |  | Pd08 | 9221736 | 9227921 | overdominance | AX-586146007 | GO:0050662 | Molecular Function:coenzyme binding | NA | NA | IPR001509 | NAD-dependent epimerase/dehydratase |
|  |  | Pd08 | 9221736 | 9227921 | overdominance | AX-586146007 | GO:0050662 | Molecular Function:coenzyme binding | NA | NA | IPR010099 | Epimerase family protein SDR39U1 |
|  |  | Pd08 | 9221736 | 9227921 | overdominance | AX-586146007 | GO:0050662 | Molecular Function:coenzyme binding | NA | NA | IPR013549 | Domain of unknown function DUF1731 |
| **Prudul26A011770T1** |  | Pd08 | 9227998 | 9228575 | overdominance | AX-586146007 | NA | NA | NA | NA | NA | NA |
| **Prudul26A004990T1** |  | Pd08 | 1,20E+07 | 1,20E+07 | dominance | AX-586144793 | GO:0043531 | Molecular Function:ADP binding | NA | NA | IPR032675 | Leucine-rich repeat domain superfamily |
|  |  | Pd08 | 1,20E+07 | 1,20E+07 | dominance | AX-586144793 | GO:0043531 | Molecular Function:ADP binding | NA | NA | IPR000157 | Toll/interleukin-1 receptor homology (TIR) domain |
|  |  | Pd08 | 1,20E+07 | 1,20E+07 | dominance | AX-586144793 | GO:0043531 | Molecular Function:ADP binding | NA | NA | IPR035897 | Toll/interleukin-1 receptor homology (TIR) domain superfamily |
|  |  | Pd08 | 1,20E+07 | 1,20E+07 | dominance | AX-586144793 | GO:0043531 | Molecular Function:ADP binding | NA | NA | IPR002182 | NB-ARC |
|  |  | Pd08 | 1,20E+07 | 1,20E+07 | dominance | AX-586144793 | GO:0043531 | Molecular Function:ADP binding | NA | NA | IPR027417 | P-loop containing nucleoside triphosphate hydrolase |
|  |  | Pd08 | 1,20E+07 | 1,20E+07 | dominance | AX-586144793 | GO:0005515 | Molecular Function:protein binding | NA | NA | IPR032675 | Leucine-rich repeat domain superfamily |
|  |  | Pd08 | 1,20E+07 | 1,20E+07 | dominance | AX-586144793 | GO:0005515 | Molecular Function:protein binding | NA | NA | IPR000157 | Toll/interleukin-1 receptor homology (TIR) domain |
|  |  | Pd08 | 1,20E+07 | 1,20E+07 | dominance | AX-586144793 | GO:0005515 | Molecular Function:protein binding | NA | NA | IPR035897 | Toll/interleukin-1 receptor homology (TIR) domain superfamily |
|  |  | Pd08 | 1,20E+07 | 1,20E+07 | dominance | AX-586144793 | GO:0005515 | Molecular Function:protein binding | NA | NA | IPR002182 | NB-ARC |
|  |  | Pd08 | 1,20E+07 | 1,20E+07 | dominance | AX-586144793 | GO:0005515 | Molecular Function:protein binding | NA | NA | IPR027417 | P-loop containing nucleoside triphosphate hydrolase |
|  |  | Pd08 | 1,20E+07 | 1,20E+07 | dominance | AX-586144793 | GO:0007165 | Biological Process:signal transduction | NA | NA | IPR032675 | Leucine-rich repeat domain superfamily |
|  |  | Pd08 | 1,20E+07 | 1,20E+07 | dominance | AX-586144793 | GO:0007165 | Biological Process:signal transduction | NA | NA | IPR000157 | Toll/interleukin-1 receptor homology (TIR) domain |
|  |  | Pd08 | 1,20E+07 | 1,20E+07 | dominance | AX-586144793 | GO:0007165 | Biological Process:signal transduction | NA | NA | IPR035897 | Toll/interleukin-1 receptor homology (TIR) domain superfamily |
|  |  | Pd08 | 1,20E+07 | 1,20E+07 | dominance | AX-586144793 | GO:0007165 | Biological Process:signal transduction | NA | NA | IPR002182 | NB-ARC |
|  |  | Pd08 | 1,20E+07 | 1,20E+07 | dominance | AX-586144793 | GO:0007165 | Biological Process:signal transduction | NA | NA | IPR027417 | P-loop containing nucleoside triphosphate hydrolase |
| **Prudul26A021354T1** |  | Pd08 | 1,20E+07 | 1,20E+07 | dominance | AX-586144793 | GO:0043531 | Molecular Function:ADP binding | NA | NA | IPR002182 | NB-ARC |
|  |  | Pd08 | 1,20E+07 | 1,20E+07 | dominance | AX-586144793 | GO:0043531 | Molecular Function:ADP binding | NA | NA | IPR035897 | Toll/interleukin-1 receptor homology (TIR) domain superfamily |
|  |  | Pd08 | 1,20E+07 | 1,20E+07 | dominance | AX-586144793 | GO:0043531 | Molecular Function:ADP binding | NA | NA | IPR000157 | Toll/interleukin-1 receptor homology (TIR) domain |
|  |  | Pd08 | 1,20E+07 | 1,20E+07 | dominance | AX-586144793 | GO:0043531 | Molecular Function:ADP binding | NA | NA | IPR027417 | P-loop containing nucleoside triphosphate hydrolase |
|  |  | Pd08 | 1,20E+07 | 1,20E+07 | dominance | AX-586144793 | GO:0005515 | Molecular Function:protein binding | NA | NA | IPR002182 | NB-ARC |
|  |  | Pd08 | 1,20E+07 | 1,20E+07 | dominance | AX-586144793 | GO:0005515 | Molecular Function:protein binding | NA | NA | IPR035897 | Toll/interleukin-1 receptor homology (TIR) domain superfamily |
|  |  | Pd08 | 1,20E+07 | 1,20E+07 | dominance | AX-586144793 | GO:0005515 | Molecular Function:protein binding | NA | NA | IPR000157 | Toll/interleukin-1 receptor homology (TIR) domain |
|  |  | Pd08 | 1,20E+07 | 1,20E+07 | dominance | AX-586144793 | GO:0005515 | Molecular Function:protein binding | NA | NA | IPR027417 | P-loop containing nucleoside triphosphate hydrolase |
|  |  | Pd08 | 1,20E+07 | 1,20E+07 | dominance | AX-586144793 | GO:0007165 | Biological Process:signal transduction | NA | NA | IPR002182 | NB-ARC |
|  |  | Pd08 | 1,20E+07 | 1,20E+07 | dominance | AX-586144793 | GO:0007165 | Biological Process:signal transduction | NA | NA | IPR035897 | Toll/interleukin-1 receptor homology (TIR) domain superfamily |
|  |  | Pd08 | 1,20E+07 | 1,20E+07 | dominance | AX-586144793 | GO:0007165 | Biological Process:signal transduction | NA | NA | IPR000157 | Toll/interleukin-1 receptor homology (TIR) domain |
|  |  | Pd08 | 1,20E+07 | 1,20E+07 | dominance | AX-586144793 | GO:0007165 | Biological Process:signal transduction | NA | NA | IPR027417 | P-loop containing nucleoside triphosphate hydrolase |
| **Prudul26A005353T1** |  | Pd08 | 1,20E+07 | 1,20E+07 | dominance | AX-586144793 | GO:0005515 | Molecular Function:protein binding | K13414 | mitogen-activated protein kinase kinase kinase 1 [EC:2.7.11.25] | IPR000157 | Toll/interleukin-1 receptor homology (TIR) domain |
|  |  | Pd08 | 1,20E+07 | 1,20E+07 | dominance | AX-586144793 | GO:0005515 | Molecular Function:protein binding | K13414 | mitogen-activated protein kinase kinase kinase 1 [EC:2.7.11.25] | IPR035897 | Toll/interleukin-1 receptor homology (TIR) domain superfamily |
|  |  | Pd08 | 1,20E+07 | 1,20E+07 | dominance | AX-586144793 | GO:0007165 | Biological Process:signal transduction | K13414 | mitogen-activated protein kinase kinase kinase 1 [EC:2.7.11.25] | IPR000157 | Toll/interleukin-1 receptor homology (TIR) domain |
|  |  | Pd08 | 1,20E+07 | 1,20E+07 | dominance | AX-586144793 | GO:0007165 | Biological Process:signal transduction | K13414 | mitogen-activated protein kinase kinase kinase 1 [EC:2.7.11.25] | IPR035897 | Toll/interleukin-1 receptor homology (TIR) domain superfamily |
| **Prudul26A030733T1** |  | Pd08 | 1,20E+07 | 1,20E+07 | dominance | AX-586144793 | NA | NA | NA | NA | IPR026992 | Non-haem dioxygenase N-terminal domain |
|  |  | Pd08 | 1,20E+07 | 1,20E+07 | dominance | AX-586144793 | NA | NA | NA | NA | IPR027443 | Isopenicillin N synthase-like |
| **Prudul26A024148T1** |  | Pd08 | 1,20E+07 | 1,20E+07 | dominance | AX-586144793 | GO:0055114 | Biological Process:oxidation-reduction process | NA | NA | IPR027443 | Isopenicillin N synthase-like |
|  |  | Pd08 | 1,20E+07 | 1,20E+07 | dominance | AX-586144793 | GO:0055114 | Biological Process:oxidation-reduction process | NA | NA | IPR005123 | Oxoglutarate/iron-dependent dioxygenase |
|  |  | Pd08 | 1,20E+07 | 1,20E+07 | dominance | AX-586144793 | GO:0016491 | Molecular Function:oxidoreductase activity | NA | NA | IPR027443 | Isopenicillin N synthase-like |
|  |  | Pd08 | 1,20E+07 | 1,20E+07 | dominance | AX-586144793 | GO:0016491 | Molecular Function:oxidoreductase activity | NA | NA | IPR005123 | Oxoglutarate/iron-dependent dioxygenase |
| **Prudul26A014969T1** |  | Pd08 | 1,20E+07 | 1,20E+07 | dominance | AX-586144793 | GO:0016310 | Biological Process:phosphorylation | K08873 | serine/threonine-protein kinase SMG1 [EC:2.7.11.1] | IPR016024 | Armadillo-type fold |
|  |  | Pd08 | 1,20E+07 | 1,20E+07 | dominance | AX-586144793 | GO:0016310 | Biological Process:phosphorylation | K08873 | serine/threonine-protein kinase SMG1 [EC:2.7.11.1] | IPR014009 | PIK-related kinase |
|  |  | Pd08 | 1,20E+07 | 1,20E+07 | dominance | AX-586144793 | GO:0016310 | Biological Process:phosphorylation | K08873 | serine/threonine-protein kinase SMG1 [EC:2.7.11.1] | IPR011009 | Protein kinase-like domain superfamily |
|  |  | Pd08 | 1,20E+07 | 1,20E+07 | dominance | AX-586144793 | GO:0016310 | Biological Process:phosphorylation | K08873 | serine/threonine-protein kinase SMG1 [EC:2.7.11.1] | IPR031559 | Serine/threonine-protein kinase SMG1 |
|  |  | Pd08 | 1,20E+07 | 1,20E+07 | dominance | AX-586144793 | GO:0016310 | Biological Process:phosphorylation | K08873 | serine/threonine-protein kinase SMG1 [EC:2.7.11.1] | IPR003152 | FATC domain |
|  |  | Pd08 | 1,20E+07 | 1,20E+07 | dominance | AX-586144793 | GO:0016310 | Biological Process:phosphorylation | K08873 | serine/threonine-protein kinase SMG1 [EC:2.7.11.1] | IPR018936 | Phosphatidylinositol 3/4-kinase, conserved site |
|  |  | Pd08 | 1,20E+07 | 1,20E+07 | dominance | AX-586144793 | GO:0016310 | Biological Process:phosphorylation | K08873 | serine/threonine-protein kinase SMG1 [EC:2.7.11.1] | IPR036940 | Phosphatidylinositol 3-/4-kinase, catalytic domain superfamily |
|  |  | Pd08 | 1,20E+07 | 1,20E+07 | dominance | AX-586144793 | GO:0016310 | Biological Process:phosphorylation | K08873 | serine/threonine-protein kinase SMG1 [EC:2.7.11.1] | IPR000403 | Phosphatidylinositol 3-/4-kinase, catalytic domain |
|  |  | Pd08 | 1,20E+07 | 1,20E+07 | dominance | AX-586144793 | GO:0004674 | Molecular Function:protein serine/threonine kinase activity | K08873 | serine/threonine-protein kinase SMG1 [EC:2.7.11.1] | IPR016024 | Armadillo-type fold |
|  |  | Pd08 | 1,20E+07 | 1,20E+07 | dominance | AX-586144793 | GO:0004674 | Molecular Function:protein serine/threonine kinase activity | K08873 | serine/threonine-protein kinase SMG1 [EC:2.7.11.1] | IPR014009 | PIK-related kinase |
|  |  | Pd08 | 1,20E+07 | 1,20E+07 | dominance | AX-586144793 | GO:0004674 | Molecular Function:protein serine/threonine kinase activity | K08873 | serine/threonine-protein kinase SMG1 [EC:2.7.11.1] | IPR011009 | Protein kinase-like domain superfamily |
|  |  | Pd08 | 1,20E+07 | 1,20E+07 | dominance | AX-586144793 | GO:0004674 | Molecular Function:protein serine/threonine kinase activity | K08873 | serine/threonine-protein kinase SMG1 [EC:2.7.11.1] | IPR031559 | Serine/threonine-protein kinase SMG1 |
|  |  | Pd08 | 1,20E+07 | 1,20E+07 | dominance | AX-586144793 | GO:0004674 | Molecular Function:protein serine/threonine kinase activity | K08873 | serine/threonine-protein kinase SMG1 [EC:2.7.11.1] | IPR003152 | FATC domain |
|  |  | Pd08 | 1,20E+07 | 1,20E+07 | dominance | AX-586144793 | GO:0004674 | Molecular Function:protein serine/threonine kinase activity | K08873 | serine/threonine-protein kinase SMG1 [EC:2.7.11.1] | IPR018936 | Phosphatidylinositol 3/4-kinase, conserved site |
|  |  | Pd08 | 1,20E+07 | 1,20E+07 | dominance | AX-586144793 | GO:0004674 | Molecular Function:protein serine/threonine kinase activity | K08873 | serine/threonine-protein kinase SMG1 [EC:2.7.11.1] | IPR036940 | Phosphatidylinositol 3-/4-kinase, catalytic domain superfamily |
|  |  | Pd08 | 1,20E+07 | 1,20E+07 | dominance | AX-586144793 | GO:0004674 | Molecular Function:protein serine/threonine kinase activity | K08873 | serine/threonine-protein kinase SMG1 [EC:2.7.11.1] | IPR000403 | Phosphatidylinositol 3-/4-kinase, catalytic domain |
|  |  | Pd08 | 1,20E+07 | 1,20E+07 | dominance | AX-586144793 | GO:0000184 | Biological Process:nuclear-transcribed mRNA catabolic process, nonsense-mediated decay | K08873 | serine/threonine-protein kinase SMG1 [EC:2.7.11.1] | IPR016024 | Armadillo-type fold |
|  |  | Pd08 | 1,20E+07 | 1,20E+07 | dominance | AX-586144793 | GO:0000184 | Biological Process:nuclear-transcribed mRNA catabolic process, nonsense-mediated decay | K08873 | serine/threonine-protein kinase SMG1 [EC:2.7.11.1] | IPR014009 | PIK-related kinase |
|  |  | Pd08 | 1,20E+07 | 1,20E+07 | dominance | AX-586144793 | GO:0000184 | Biological Process:nuclear-transcribed mRNA catabolic process, nonsense-mediated decay | K08873 | serine/threonine-protein kinase SMG1 [EC:2.7.11.1] | IPR011009 | Protein kinase-like domain superfamily |
|  |  | Pd08 | 1,20E+07 | 1,20E+07 | dominance | AX-586144793 | GO:0000184 | Biological Process:nuclear-transcribed mRNA catabolic process, nonsense-mediated decay | K08873 | serine/threonine-protein kinase SMG1 [EC:2.7.11.1] | IPR031559 | Serine/threonine-protein kinase SMG1 |
|  |  | Pd08 | 1,20E+07 | 1,20E+07 | dominance | AX-586144793 | GO:0000184 | Biological Process:nuclear-transcribed mRNA catabolic process, nonsense-mediated decay | K08873 | serine/threonine-protein kinase SMG1 [EC:2.7.11.1] | IPR003152 | FATC domain |
|  |  | Pd08 | 1,20E+07 | 1,20E+07 | dominance | AX-586144793 | GO:0000184 | Biological Process:nuclear-transcribed mRNA catabolic process, nonsense-mediated decay | K08873 | serine/threonine-protein kinase SMG1 [EC:2.7.11.1] | IPR018936 | Phosphatidylinositol 3/4-kinase, conserved site |
|  |  | Pd08 | 1,20E+07 | 1,20E+07 | dominance | AX-586144793 | GO:0000184 | Biological Process:nuclear-transcribed mRNA catabolic process, nonsense-mediated decay | K08873 | serine/threonine-protein kinase SMG1 [EC:2.7.11.1] | IPR036940 | Phosphatidylinositol 3-/4-kinase, catalytic domain superfamily |
|  |  | Pd08 | 1,20E+07 | 1,20E+07 | dominance | AX-586144793 | GO:0000184 | Biological Process:nuclear-transcribed mRNA catabolic process, nonsense-mediated decay | K08873 | serine/threonine-protein kinase SMG1 [EC:2.7.11.1] | IPR000403 | Phosphatidylinositol 3-/4-kinase, catalytic domain |
|  |  | Pd08 | 1,20E+07 | 1,20E+07 | dominance | AX-586144793 | GO:0005515 | Molecular Function:protein binding | K08873 | serine/threonine-protein kinase SMG1 [EC:2.7.11.1] | IPR016024 | Armadillo-type fold |
|  |  | Pd08 | 1,20E+07 | 1,20E+07 | dominance | AX-586144793 | GO:0005515 | Molecular Function:protein binding | K08873 | serine/threonine-protein kinase SMG1 [EC:2.7.11.1] | IPR014009 | PIK-related kinase |
|  |  | Pd08 | 1,20E+07 | 1,20E+07 | dominance | AX-586144793 | GO:0005515 | Molecular Function:protein binding | K08873 | serine/threonine-protein kinase SMG1 [EC:2.7.11.1] | IPR011009 | Protein kinase-like domain superfamily |
|  |  | Pd08 | 1,20E+07 | 1,20E+07 | dominance | AX-586144793 | GO:0005515 | Molecular Function:protein binding | K08873 | serine/threonine-protein kinase SMG1 [EC:2.7.11.1] | IPR031559 | Serine/threonine-protein kinase SMG1 |
|  |  | Pd08 | 1,20E+07 | 1,20E+07 | dominance | AX-586144793 | GO:0005515 | Molecular Function:protein binding | K08873 | serine/threonine-protein kinase SMG1 [EC:2.7.11.1] | IPR003152 | FATC domain |
|  |  | Pd08 | 1,20E+07 | 1,20E+07 | dominance | AX-586144793 | GO:0005515 | Molecular Function:protein binding | K08873 | serine/threonine-protein kinase SMG1 [EC:2.7.11.1] | IPR018936 | Phosphatidylinositol 3/4-kinase, conserved site |
|  |  | Pd08 | 1,20E+07 | 1,20E+07 | dominance | AX-586144793 | GO:0005515 | Molecular Function:protein binding | K08873 | serine/threonine-protein kinase SMG1 [EC:2.7.11.1] | IPR036940 | Phosphatidylinositol 3-/4-kinase, catalytic domain superfamily |
|  |  | Pd08 | 1,20E+07 | 1,20E+07 | dominance | AX-586144793 | GO:0005515 | Molecular Function:protein binding | K08873 | serine/threonine-protein kinase SMG1 [EC:2.7.11.1] | IPR000403 | Phosphatidylinositol 3-/4-kinase, catalytic domain |
|  |  | Pd08 | 1,20E+07 | 1,20E+07 | dominance | AX-586144793 | GO:0016301 | Molecular Function:kinase activity | K08873 | serine/threonine-protein kinase SMG1 [EC:2.7.11.1] | IPR016024 | Armadillo-type fold |
|  |  | Pd08 | 1,20E+07 | 1,20E+07 | dominance | AX-586144793 | GO:0016301 | Molecular Function:kinase activity | K08873 | serine/threonine-protein kinase SMG1 [EC:2.7.11.1] | IPR014009 | PIK-related kinase |
|  |  | Pd08 | 1,20E+07 | 1,20E+07 | dominance | AX-586144793 | GO:0016301 | Molecular Function:kinase activity | K08873 | serine/threonine-protein kinase SMG1 [EC:2.7.11.1] | IPR011009 | Protein kinase-like domain superfamily |
|  |  | Pd08 | 1,20E+07 | 1,20E+07 | dominance | AX-586144793 | GO:0016301 | Molecular Function:kinase activity | K08873 | serine/threonine-protein kinase SMG1 [EC:2.7.11.1] | IPR031559 | Serine/threonine-protein kinase SMG1 |
|  |  | Pd08 | 1,20E+07 | 1,20E+07 | dominance | AX-586144793 | GO:0016301 | Molecular Function:kinase activity | K08873 | serine/threonine-protein kinase SMG1 [EC:2.7.11.1] | IPR003152 | FATC domain |
|  |  | Pd08 | 1,20E+07 | 1,20E+07 | dominance | AX-586144793 | GO:0016301 | Molecular Function:kinase activity | K08873 | serine/threonine-protein kinase SMG1 [EC:2.7.11.1] | IPR018936 | Phosphatidylinositol 3/4-kinase, conserved site |
|  |  | Pd08 | 1,20E+07 | 1,20E+07 | dominance | AX-586144793 | GO:0016301 | Molecular Function:kinase activity | K08873 | serine/threonine-protein kinase SMG1 [EC:2.7.11.1] | IPR036940 | Phosphatidylinositol 3-/4-kinase, catalytic domain superfamily |
|  |  | Pd08 | 1,20E+07 | 1,20E+07 | dominance | AX-586144793 | GO:0016301 | Molecular Function:kinase activity | K08873 | serine/threonine-protein kinase SMG1 [EC:2.7.11.1] | IPR000403 | Phosphatidylinositol 3-/4-kinase, catalytic domain |
| **Prudul26A014969T2** |  | Pd08 | 1,20E+07 | 1,20E+07 | dominance | AX-586144793 | GO:0005515 | Molecular Function:protein binding | K08873 | serine/threonine-protein kinase SMG1 [EC:2.7.11.1] | IPR018936 | Phosphatidylinositol 3/4-kinase, conserved site |
|  |  | Pd08 | 1,20E+07 | 1,20E+07 | dominance | AX-586144793 | GO:0005515 | Molecular Function:protein binding | K08873 | serine/threonine-protein kinase SMG1 [EC:2.7.11.1] | IPR031559 | Serine/threonine-protein kinase SMG1 |
|  |  | Pd08 | 1,20E+07 | 1,20E+07 | dominance | AX-586144793 | GO:0005515 | Molecular Function:protein binding | K08873 | serine/threonine-protein kinase SMG1 [EC:2.7.11.1] | IPR036940 | Phosphatidylinositol 3-/4-kinase, catalytic domain superfamily |
|  |  | Pd08 | 1,20E+07 | 1,20E+07 | dominance | AX-586144793 | GO:0005515 | Molecular Function:protein binding | K08873 | serine/threonine-protein kinase SMG1 [EC:2.7.11.1] | IPR016024 | Armadillo-type fold |
|  |  | Pd08 | 1,20E+07 | 1,20E+07 | dominance | AX-586144793 | GO:0005515 | Molecular Function:protein binding | K08873 | serine/threonine-protein kinase SMG1 [EC:2.7.11.1] | IPR000403 | Phosphatidylinositol 3-/4-kinase, catalytic domain |
|  |  | Pd08 | 1,20E+07 | 1,20E+07 | dominance | AX-586144793 | GO:0005515 | Molecular Function:protein binding | K08873 | serine/threonine-protein kinase SMG1 [EC:2.7.11.1] | IPR014009 | PIK-related kinase |
|  |  | Pd08 | 1,20E+07 | 1,20E+07 | dominance | AX-586144793 | GO:0005515 | Molecular Function:protein binding | K08873 | serine/threonine-protein kinase SMG1 [EC:2.7.11.1] | IPR011009 | Protein kinase-like domain superfamily |
|  |  | Pd08 | 1,20E+07 | 1,20E+07 | dominance | AX-586144793 | GO:0005515 | Molecular Function:protein binding | K08873 | serine/threonine-protein kinase SMG1 [EC:2.7.11.1] | IPR003152 | FATC domain |
|  |  | Pd08 | 1,20E+07 | 1,20E+07 | dominance | AX-586144793 | GO:0004674 | Molecular Function:protein serine/threonine kinase activity | K08873 | serine/threonine-protein kinase SMG1 [EC:2.7.11.1] | IPR018936 | Phosphatidylinositol 3/4-kinase, conserved site |
|  |  | Pd08 | 1,20E+07 | 1,20E+07 | dominance | AX-586144793 | GO:0004674 | Molecular Function:protein serine/threonine kinase activity | K08873 | serine/threonine-protein kinase SMG1 [EC:2.7.11.1] | IPR031559 | Serine/threonine-protein kinase SMG1 |
|  |  | Pd08 | 1,20E+07 | 1,20E+07 | dominance | AX-586144793 | GO:0004674 | Molecular Function:protein serine/threonine kinase activity | K08873 | serine/threonine-protein kinase SMG1 [EC:2.7.11.1] | IPR036940 | Phosphatidylinositol 3-/4-kinase, catalytic domain superfamily |
|  |  | Pd08 | 1,20E+07 | 1,20E+07 | dominance | AX-586144793 | GO:0004674 | Molecular Function:protein serine/threonine kinase activity | K08873 | serine/threonine-protein kinase SMG1 [EC:2.7.11.1] | IPR016024 | Armadillo-type fold |
|  |  | Pd08 | 1,20E+07 | 1,20E+07 | dominance | AX-586144793 | GO:0004674 | Molecular Function:protein serine/threonine kinase activity | K08873 | serine/threonine-protein kinase SMG1 [EC:2.7.11.1] | IPR000403 | Phosphatidylinositol 3-/4-kinase, catalytic domain |
|  |  | Pd08 | 1,20E+07 | 1,20E+07 | dominance | AX-586144793 | GO:0004674 | Molecular Function:protein serine/threonine kinase activity | K08873 | serine/threonine-protein kinase SMG1 [EC:2.7.11.1] | IPR014009 | PIK-related kinase |
|  |  | Pd08 | 1,20E+07 | 1,20E+07 | dominance | AX-586144793 | GO:0004674 | Molecular Function:protein serine/threonine kinase activity | K08873 | serine/threonine-protein kinase SMG1 [EC:2.7.11.1] | IPR011009 | Protein kinase-like domain superfamily |
|  |  | Pd08 | 1,20E+07 | 1,20E+07 | dominance | AX-586144793 | GO:0004674 | Molecular Function:protein serine/threonine kinase activity | K08873 | serine/threonine-protein kinase SMG1 [EC:2.7.11.1] | IPR003152 | FATC domain |
|  |  | Pd08 | 1,20E+07 | 1,20E+07 | dominance | AX-586144793 | GO:0016310 | Biological Process:phosphorylation | K08873 | serine/threonine-protein kinase SMG1 [EC:2.7.11.1] | IPR018936 | Phosphatidylinositol 3/4-kinase, conserved site |
|  |  | Pd08 | 1,20E+07 | 1,20E+07 | dominance | AX-586144793 | GO:0016310 | Biological Process:phosphorylation | K08873 | serine/threonine-protein kinase SMG1 [EC:2.7.11.1] | IPR031559 | Serine/threonine-protein kinase SMG1 |
|  |  | Pd08 | 1,20E+07 | 1,20E+07 | dominance | AX-586144793 | GO:0016310 | Biological Process:phosphorylation | K08873 | serine/threonine-protein kinase SMG1 [EC:2.7.11.1] | IPR036940 | Phosphatidylinositol 3-/4-kinase, catalytic domain superfamily |
|  |  | Pd08 | 1,20E+07 | 1,20E+07 | dominance | AX-586144793 | GO:0016310 | Biological Process:phosphorylation | K08873 | serine/threonine-protein kinase SMG1 [EC:2.7.11.1] | IPR016024 | Armadillo-type fold |
|  |  | Pd08 | 1,20E+07 | 1,20E+07 | dominance | AX-586144793 | GO:0016310 | Biological Process:phosphorylation | K08873 | serine/threonine-protein kinase SMG1 [EC:2.7.11.1] | IPR000403 | Phosphatidylinositol 3-/4-kinase, catalytic domain |
|  |  | Pd08 | 1,20E+07 | 1,20E+07 | dominance | AX-586144793 | GO:0016310 | Biological Process:phosphorylation | K08873 | serine/threonine-protein kinase SMG1 [EC:2.7.11.1] | IPR014009 | PIK-related kinase |
|  |  | Pd08 | 1,20E+07 | 1,20E+07 | dominance | AX-586144793 | GO:0016310 | Biological Process:phosphorylation | K08873 | serine/threonine-protein kinase SMG1 [EC:2.7.11.1] | IPR011009 | Protein kinase-like domain superfamily |
|  |  | Pd08 | 1,20E+07 | 1,20E+07 | dominance | AX-586144793 | GO:0016310 | Biological Process:phosphorylation | K08873 | serine/threonine-protein kinase SMG1 [EC:2.7.11.1] | IPR003152 | FATC domain |
|  |  | Pd08 | 1,20E+07 | 1,20E+07 | dominance | AX-586144793 | GO:0016301 | Molecular Function:kinase activity | K08873 | serine/threonine-protein kinase SMG1 [EC:2.7.11.1] | IPR018936 | Phosphatidylinositol 3/4-kinase, conserved site |
|  |  | Pd08 | 1,20E+07 | 1,20E+07 | dominance | AX-586144793 | GO:0016301 | Molecular Function:kinase activity | K08873 | serine/threonine-protein kinase SMG1 [EC:2.7.11.1] | IPR031559 | Serine/threonine-protein kinase SMG1 |
|  |  | Pd08 | 1,20E+07 | 1,20E+07 | dominance | AX-586144793 | GO:0016301 | Molecular Function:kinase activity | K08873 | serine/threonine-protein kinase SMG1 [EC:2.7.11.1] | IPR036940 | Phosphatidylinositol 3-/4-kinase, catalytic domain superfamily |
|  |  | Pd08 | 1,20E+07 | 1,20E+07 | dominance | AX-586144793 | GO:0016301 | Molecular Function:kinase activity | K08873 | serine/threonine-protein kinase SMG1 [EC:2.7.11.1] | IPR016024 | Armadillo-type fold |
|  |  | Pd08 | 1,20E+07 | 1,20E+07 | dominance | AX-586144793 | GO:0016301 | Molecular Function:kinase activity | K08873 | serine/threonine-protein kinase SMG1 [EC:2.7.11.1] | IPR000403 | Phosphatidylinositol 3-/4-kinase, catalytic domain |
|  |  | Pd08 | 1,20E+07 | 1,20E+07 | dominance | AX-586144793 | GO:0016301 | Molecular Function:kinase activity | K08873 | serine/threonine-protein kinase SMG1 [EC:2.7.11.1] | IPR014009 | PIK-related kinase |
|  |  | Pd08 | 1,20E+07 | 1,20E+07 | dominance | AX-586144793 | GO:0016301 | Molecular Function:kinase activity | K08873 | serine/threonine-protein kinase SMG1 [EC:2.7.11.1] | IPR011009 | Protein kinase-like domain superfamily |
|  |  | Pd08 | 1,20E+07 | 1,20E+07 | dominance | AX-586144793 | GO:0016301 | Molecular Function:kinase activity | K08873 | serine/threonine-protein kinase SMG1 [EC:2.7.11.1] | IPR003152 | FATC domain |
|  |  | Pd08 | 1,20E+07 | 1,20E+07 | dominance | AX-586144793 | GO:0000184 | Biological Process:nuclear-transcribed mRNA catabolic process, nonsense-mediated decay | K08873 | serine/threonine-protein kinase SMG1 [EC:2.7.11.1] | IPR018936 | Phosphatidylinositol 3/4-kinase, conserved site |
|  |  | Pd08 | 1,20E+07 | 1,20E+07 | dominance | AX-586144793 | GO:0000184 | Biological Process:nuclear-transcribed mRNA catabolic process, nonsense-mediated decay | K08873 | serine/threonine-protein kinase SMG1 [EC:2.7.11.1] | IPR031559 | Serine/threonine-protein kinase SMG1 |
|  |  | Pd08 | 1,20E+07 | 1,20E+07 | dominance | AX-586144793 | GO:0000184 | Biological Process:nuclear-transcribed mRNA catabolic process, nonsense-mediated decay | K08873 | serine/threonine-protein kinase SMG1 [EC:2.7.11.1] | IPR036940 | Phosphatidylinositol 3-/4-kinase, catalytic domain superfamily |
|  |  | Pd08 | 1,20E+07 | 1,20E+07 | dominance | AX-586144793 | GO:0000184 | Biological Process:nuclear-transcribed mRNA catabolic process, nonsense-mediated decay | K08873 | serine/threonine-protein kinase SMG1 [EC:2.7.11.1] | IPR016024 | Armadillo-type fold |
|  |  | Pd08 | 1,20E+07 | 1,20E+07 | dominance | AX-586144793 | GO:0000184 | Biological Process:nuclear-transcribed mRNA catabolic process, nonsense-mediated decay | K08873 | serine/threonine-protein kinase SMG1 [EC:2.7.11.1] | IPR000403 | Phosphatidylinositol 3-/4-kinase, catalytic domain |
|  |  | Pd08 | 1,20E+07 | 1,20E+07 | dominance | AX-586144793 | GO:0000184 | Biological Process:nuclear-transcribed mRNA catabolic process, nonsense-mediated decay | K08873 | serine/threonine-protein kinase SMG1 [EC:2.7.11.1] | IPR014009 | PIK-related kinase |
|  |  | Pd08 | 1,20E+07 | 1,20E+07 | dominance | AX-586144793 | GO:0000184 | Biological Process:nuclear-transcribed mRNA catabolic process, nonsense-mediated decay | K08873 | serine/threonine-protein kinase SMG1 [EC:2.7.11.1] | IPR011009 | Protein kinase-like domain superfamily |
|  |  | Pd08 | 1,20E+07 | 1,20E+07 | dominance | AX-586144793 | GO:0000184 | Biological Process:nuclear-transcribed mRNA catabolic process, nonsense-mediated decay | K08873 | serine/threonine-protein kinase SMG1 [EC:2.7.11.1] | IPR003152 | FATC domain |
| **Prudul26A027410T1** | Downstream gene variant | Pd08 | 1,20E+07 | 1,20E+07 | dominance | AX-586144793 | NA | NA | K15455 | diphthamide biosynthesis protein 3 | IPR036671 | DPH Zinc finger superfamily |
|  |  | Pd08 | 1,20E+07 | 1,20E+07 | dominance | AX-586144793 | NA | NA | K15455 | diphthamide biosynthesis protein 3 | IPR007872 | Zinc finger, DPH-type |
| **Prudul26A018525T1** | Upstream gene variant | Pd08 | 1,20E+07 | 1,20E+07 | dominance | AX-586144793 | NA | NA | NA | NA | IPR006904 | Protein of unknown function DUF716 (TMEM45) |
| **Prudul26A020986T1** |  | Pd08 | 1,20E+07 | 1,20E+07 | dominance | AX-586144793 | GO:0009512 | Cellular Component:cytochrome b6f complex | NA | NA | IPR012595 | PetM of cytochrome b6/f complex subunit 7 |
| **Prudul26A003860T1** |  | Pd08 | 1,20E+07 | 1,20E+07 | dominance | AX-586144793 | GO:0003677 | Molecular Function:DNA binding | NA | NA | IPR003340 | B3 DNA binding domain |
|  |  | Pd08 | 1,20E+07 | 1,20E+07 | dominance | AX-586144793 | GO:0003677 | Molecular Function:DNA binding | NA | NA | IPR015300 | DNA-binding pseudobarrel domain superfamily |
| **Prudul26A003860T2** |  | Pd08 | 1,20E+07 | 1,20E+07 | dominance | AX-586144793 | GO:0003677 | Molecular Function:DNA binding | NA | NA | IPR003340 | B3 DNA binding domain |
|  |  | Pd08 | 1,20E+07 | 1,20E+07 | dominance | AX-586144793 | GO:0003677 | Molecular Function:DNA binding | NA | NA | IPR015300 | DNA-binding pseudobarrel domain superfamily |
| **Prudul26A011889T1** |  | Pd08 | 1,20E+07 | 1,20E+07 | dominance | AX-586144793 | GO:0003677 | Molecular Function:DNA binding | NA | NA | IPR003340 | B3 DNA binding domain |
|  |  | Pd08 | 1,20E+07 | 1,20E+07 | dominance | AX-586144793 | GO:0003677 | Molecular Function:DNA binding | NA | NA | IPR015300 | DNA-binding pseudobarrel domain superfamily |
| **Prudul26A020321T1** |  | Pd08 | 1,20E+07 | 1,20E+07 | dominance | AX-586144793 | NA | NA | NA | NA | NA | NA |
| **Prudul26A033100T1** |  | Pd08 | 1,20E+07 | 1,20E+07 | dominance | AX-586144793 | NA | NA | NA | NA | IPR018866 | Zinc-finger domain of monoamine-oxidase A repressor R1 |
| **Prudul26A000091T1** |  | Pd08 | 1,20E+07 | 1,20E+07 | dominance | AX-586144793 | GO:0035091 | Molecular Function:phosphatidylinositol binding | NA | NA | IPR036871 | PX domain superfamily |
|  |  | Pd08 | 1,20E+07 | 1,20E+07 | dominance | AX-586144793 | GO:0035091 | Molecular Function:phosphatidylinositol binding | NA | NA | IPR013937 | Sorting nexin, C-terminal |
|  |  | Pd08 | 1,20E+07 | 1,20E+07 | dominance | AX-586144793 | GO:0035091 | Molecular Function:phosphatidylinositol binding | NA | NA | IPR003114 | Phox-associated domain |
|  |  | Pd08 | 1,20E+07 | 1,20E+07 | dominance | AX-586144793 | GO:0035091 | Molecular Function:phosphatidylinositol binding | NA | NA | IPR001683 | Phox homologous domain |
| **Prudul26A000091T2** |  | Pd08 | 1,20E+07 | 1,20E+07 | dominance | AX-586144793 | GO:0035091 | Molecular Function:phosphatidylinositol binding | NA | NA | IPR036871 | PX domain superfamily |
|  |  | Pd08 | 1,20E+07 | 1,20E+07 | dominance | AX-586144793 | GO:0035091 | Molecular Function:phosphatidylinositol binding | NA | NA | IPR001683 | Phox homologous domain |
|  |  | Pd08 | 1,20E+07 | 1,20E+07 | dominance | AX-586144793 | GO:0035091 | Molecular Function:phosphatidylinositol binding | NA | NA | IPR003114 | Phox-associated domain |
|  |  | Pd08 | 1,20E+07 | 1,20E+07 | dominance | AX-586144793 | GO:0035091 | Molecular Function:phosphatidylinositol binding | NA | NA | IPR013937 | Sorting nexin, C-terminal |
| **Prudul26A010132T1** |  | Pd08 | 1,20E+07 | 1,20E+07 | dominance | AX-586144793 | NA | NA | NA | NA | NA | NA |
| **Prudul26A006094T1** |  | Pd08 | 1,70E+07 | 1,70E+07 | recessive | AX-586150343 | NA | NA | NA | NA | NA | NA |
| **Prudul26A020405T1** |  | Pd08 | 1,70E+07 | 1,70E+07 | recessive | AX-586150343 | NA | NA | NA | NA | IPR021883 | Protein LOW PSII ACCUMULATION 1-like |
| **Prudul26A027804T1** |  | Pd08 | 1,70E+07 | 1,70E+07 | recessive | AX-586150343 | GO:0009522 | Cellular Component:photosystem I | K02693 | photosystem I subunit IV | IPR008990 | Electron transport accessory-like domain superfamily |
|  |  | Pd08 | 1,70E+07 | 1,70E+07 | recessive | AX-586150343 | GO:0009522 | Cellular Component:photosystem I | K02693 | photosystem I subunit IV | IPR003375 | Photosystem I PsaE, reaction centre subunit IV |
|  |  | Pd08 | 1,70E+07 | 1,70E+07 | recessive | AX-586150343 | GO:0015979 | Biological Process:photosynthesis | K02693 | photosystem I subunit IV | IPR008990 | Electron transport accessory-like domain superfamily |
|  |  | Pd08 | 1,70E+07 | 1,70E+07 | recessive | AX-586150343 | GO:0015979 | Biological Process:photosynthesis | K02693 | photosystem I subunit IV | IPR003375 | Photosystem I PsaE, reaction centre subunit IV |
|  |  | Pd08 | 1,70E+07 | 1,70E+07 | recessive | AX-586150343 | GO:0009538 | Cellular Component:photosystem I reaction center | K02693 | photosystem I subunit IV | IPR008990 | Electron transport accessory-like domain superfamily |
|  |  | Pd08 | 1,70E+07 | 1,70E+07 | recessive | AX-586150343 | GO:0009538 | Cellular Component:photosystem I reaction center | K02693 | photosystem I subunit IV | IPR003375 | Photosystem I PsaE, reaction centre subunit IV |
| **Prudul26A027804T2** |  | Pd08 | 1,70E+07 | 1,70E+07 | recessive | AX-586150343 | GO:0009522 | Cellular Component:photosystem I | K02693 | photosystem I subunit IV | IPR008990 | Electron transport accessory-like domain superfamily |
|  |  | Pd08 | 1,70E+07 | 1,70E+07 | recessive | AX-586150343 | GO:0009522 | Cellular Component:photosystem I | K02693 | photosystem I subunit IV | IPR003375 | Photosystem I PsaE, reaction centre subunit IV |
|  |  | Pd08 | 1,70E+07 | 1,70E+07 | recessive | AX-586150343 | GO:0015979 | Biological Process:photosynthesis | K02693 | photosystem I subunit IV | IPR008990 | Electron transport accessory-like domain superfamily |
|  |  | Pd08 | 1,70E+07 | 1,70E+07 | recessive | AX-586150343 | GO:0015979 | Biological Process:photosynthesis | K02693 | photosystem I subunit IV | IPR003375 | Photosystem I PsaE, reaction centre subunit IV |
|  |  | Pd08 | 1,70E+07 | 1,70E+07 | recessive | AX-586150343 | GO:0009538 | Cellular Component:photosystem I reaction center | K02693 | photosystem I subunit IV | IPR008990 | Electron transport accessory-like domain superfamily |
|  |  | Pd08 | 1,70E+07 | 1,70E+07 | recessive | AX-586150343 | GO:0009538 | Cellular Component:photosystem I reaction center | K02693 | photosystem I subunit IV | IPR003375 | Photosystem I PsaE, reaction centre subunit IV |
| **Prudul26A020405T2** |  | Pd08 | 1,70E+07 | 1,70E+07 | recessive | AX-586150343 | NA | NA | NA | NA | IPR021883 | Protein LOW PSII ACCUMULATION 1-like |
| **Prudul26A000377T1** |  | Pd08 | 1,70E+07 | 1,70E+07 | recessive | AX-586150343 | GO:0005515 | Molecular Function:protein binding | K00799 | glutathione S-transferase [EC:2.5.1.18] | IPR004046 | Glutathione S-transferase, C-terminal |
|  |  | Pd08 | 1,70E+07 | 1,70E+07 | recessive | AX-586150343 | GO:0005515 | Molecular Function:protein binding | K00799 | glutathione S-transferase [EC:2.5.1.18] | IPR036282 | Glutathione S-transferase, C-terminal domain superfamily |
|  |  | Pd08 | 1,70E+07 | 1,70E+07 | recessive | AX-586150343 | GO:0005515 | Molecular Function:protein binding | K00799 | glutathione S-transferase [EC:2.5.1.18] | IPR004045 | Glutathione S-transferase, N-terminal |
|  |  | Pd08 | 1,70E+07 | 1,70E+07 | recessive | AX-586150343 | GO:0005515 | Molecular Function:protein binding | K00799 | glutathione S-transferase [EC:2.5.1.18] | IPR036249 | Thioredoxin-like superfamily |
|  |  | Pd08 | 1,70E+07 | 1,70E+07 | recessive | AX-586150343 | GO:0005515 | Molecular Function:protein binding | K00799 | glutathione S-transferase [EC:2.5.1.18] | IPR010987 | Glutathione S-transferase, C-terminal-like |
| **Prudul26A026311T1** |  | Pd08 | 1,70E+07 | 1,70E+07 | recessive | AX-586150343 | GO:0005515 | Molecular Function:protein binding | K00799 | glutathione S-transferase [EC:2.5.1.18] | IPR036282 | Glutathione S-transferase, C-terminal domain superfamily |
|  |  | Pd08 | 1,70E+07 | 1,70E+07 | recessive | AX-586150343 | GO:0005515 | Molecular Function:protein binding | K00799 | glutathione S-transferase [EC:2.5.1.18] | IPR010987 | Glutathione S-transferase, C-terminal-like |
|  |  | Pd08 | 1,70E+07 | 1,70E+07 | recessive | AX-586150343 | GO:0005515 | Molecular Function:protein binding | K00799 | glutathione S-transferase [EC:2.5.1.18] | IPR004045 | Glutathione S-transferase, N-terminal |
|  |  | Pd08 | 1,70E+07 | 1,70E+07 | recessive | AX-586150343 | GO:0005515 | Molecular Function:protein binding | K00799 | glutathione S-transferase [EC:2.5.1.18] | IPR036249 | Thioredoxin-like superfamily |
|  |  | Pd08 | 1,70E+07 | 1,70E+07 | recessive | AX-586150343 | GO:0005515 | Molecular Function:protein binding | K00799 | glutathione S-transferase [EC:2.5.1.18] | IPR004046 | Glutathione S-transferase, C-terminal |
| **Prudul26A032833T1** |  | Pd08 | 1,70E+07 | 1,70E+07 | recessive | AX-586150343 | GO:0005515 | Molecular Function:protein binding | K00799 | glutathione S-transferase [EC:2.5.1.18] | IPR004046 | Glutathione S-transferase, C-terminal |
|  |  | Pd08 | 1,70E+07 | 1,70E+07 | recessive | AX-586150343 | GO:0005515 | Molecular Function:protein binding | K00799 | glutathione S-transferase [EC:2.5.1.18] | IPR036249 | Thioredoxin-like superfamily |
|  |  | Pd08 | 1,70E+07 | 1,70E+07 | recessive | AX-586150343 | GO:0005515 | Molecular Function:protein binding | K00799 | glutathione S-transferase [EC:2.5.1.18] | IPR036282 | Glutathione S-transferase, C-terminal domain superfamily |
|  |  | Pd08 | 1,70E+07 | 1,70E+07 | recessive | AX-586150343 | GO:0005515 | Molecular Function:protein binding | K00799 | glutathione S-transferase [EC:2.5.1.18] | IPR004045 | Glutathione S-transferase, N-terminal |
|  |  | Pd08 | 1,70E+07 | 1,70E+07 | recessive | AX-586150343 | GO:0005515 | Molecular Function:protein binding | K00799 | glutathione S-transferase [EC:2.5.1.18] | IPR010987 | Glutathione S-transferase, C-terminal-like |
| **Prudul26A009004T1** |  | Pd08 | 1,70E+07 | 1,70E+07 | recessive | AX-586150343 | GO:0005515 | Molecular Function:protein binding | K00799 | glutathione S-transferase [EC:2.5.1.18] | IPR036282 | Glutathione S-transferase, C-terminal domain superfamily |
|  |  | Pd08 | 1,70E+07 | 1,70E+07 | recessive | AX-586150343 | GO:0005515 | Molecular Function:protein binding | K00799 | glutathione S-transferase [EC:2.5.1.18] | IPR004046 | Glutathione S-transferase, C-terminal |
|  |  | Pd08 | 1,70E+07 | 1,70E+07 | recessive | AX-586150343 | GO:0005515 | Molecular Function:protein binding | K00799 | glutathione S-transferase [EC:2.5.1.18] | IPR036249 | Thioredoxin-like superfamily |
|  |  | Pd08 | 1,70E+07 | 1,70E+07 | recessive | AX-586150343 | GO:0005515 | Molecular Function:protein binding | K00799 | glutathione S-transferase [EC:2.5.1.18] | IPR010987 | Glutathione S-transferase, C-terminal-like |
|  |  | Pd08 | 1,70E+07 | 1,70E+07 | recessive | AX-586150343 | GO:0005515 | Molecular Function:protein binding | K00799 | glutathione S-transferase [EC:2.5.1.18] | IPR004045 | Glutathione S-transferase, N-terminal |
| **Prudul26A009004T2** |  | Pd08 | 1,70E+07 | 1,70E+07 | recessive | AX-586150343 | GO:0005515 | Molecular Function:protein binding | K00799 | glutathione S-transferase [EC:2.5.1.18] | IPR004046 | Glutathione S-transferase, C-terminal |
|  |  | Pd08 | 1,70E+07 | 1,70E+07 | recessive | AX-586150343 | GO:0005515 | Molecular Function:protein binding | K00799 | glutathione S-transferase [EC:2.5.1.18] | IPR036249 | Thioredoxin-like superfamily |
|  |  | Pd08 | 1,70E+07 | 1,70E+07 | recessive | AX-586150343 | GO:0005515 | Molecular Function:protein binding | K00799 | glutathione S-transferase [EC:2.5.1.18] | IPR004045 | Glutathione S-transferase, N-terminal |
|  |  | Pd08 | 1,70E+07 | 1,70E+07 | recessive | AX-586150343 | GO:0005515 | Molecular Function:protein binding | K00799 | glutathione S-transferase [EC:2.5.1.18] | IPR010987 | Glutathione S-transferase, C-terminal-like |
|  |  | Pd08 | 1,70E+07 | 1,70E+07 | recessive | AX-586150343 | GO:0005515 | Molecular Function:protein binding | K00799 | glutathione S-transferase [EC:2.5.1.18] | IPR036282 | Glutathione S-transferase, C-terminal domain superfamily |
| **Prudul26A000876T1** |  | Pd08 | 1,70E+07 | 1,70E+07 | recessive | AX-586150343 | GO:0045454 | Biological Process:cell redox homeostasis | NA | NA | IPR011899 | Glutaredoxin, eukaryotic/virial |
|  |  | Pd08 | 1,70E+07 | 1,70E+07 | recessive | AX-586150343 | GO:0045454 | Biological Process:cell redox homeostasis | NA | NA | IPR036249 | Thioredoxin-like superfamily |
|  |  | Pd08 | 1,70E+07 | 1,70E+07 | recessive | AX-586150343 | GO:0045454 | Biological Process:cell redox homeostasis | NA | NA | IPR002109 | Glutaredoxin |
|  |  | Pd08 | 1,70E+07 | 1,70E+07 | recessive | AX-586150343 | GO:0045454 | Biological Process:cell redox homeostasis | NA | NA | IPR014025 | Glutaredoxin subgroup |
|  |  | Pd08 | 1,70E+07 | 1,70E+07 | recessive | AX-586150343 | GO:0009055 | Molecular Function:electron transfer activity | NA | NA | IPR011899 | Glutaredoxin, eukaryotic/virial |
|  |  | Pd08 | 1,70E+07 | 1,70E+07 | recessive | AX-586150343 | GO:0009055 | Molecular Function:electron transfer activity | NA | NA | IPR036249 | Thioredoxin-like superfamily |
|  |  | Pd08 | 1,70E+07 | 1,70E+07 | recessive | AX-586150343 | GO:0009055 | Molecular Function:electron transfer activity | NA | NA | IPR002109 | Glutaredoxin |
|  |  | Pd08 | 1,70E+07 | 1,70E+07 | recessive | AX-586150343 | GO:0009055 | Molecular Function:electron transfer activity | NA | NA | IPR014025 | Glutaredoxin subgroup |
|  |  | Pd08 | 1,70E+07 | 1,70E+07 | recessive | AX-586150343 | GO:0015035 | Molecular Function:protein disulfide oxidoreductase activity | NA | NA | IPR011899 | Glutaredoxin, eukaryotic/virial |
|  |  | Pd08 | 1,70E+07 | 1,70E+07 | recessive | AX-586150343 | GO:0015035 | Molecular Function:protein disulfide oxidoreductase activity | NA | NA | IPR036249 | Thioredoxin-like superfamily |
|  |  | Pd08 | 1,70E+07 | 1,70E+07 | recessive | AX-586150343 | GO:0015035 | Molecular Function:protein disulfide oxidoreductase activity | NA | NA | IPR002109 | Glutaredoxin |
|  |  | Pd08 | 1,70E+07 | 1,70E+07 | recessive | AX-586150343 | GO:0015035 | Molecular Function:protein disulfide oxidoreductase activity | NA | NA | IPR014025 | Glutaredoxin subgroup |
| **Prudul26A004172T1** |  | Pd08 | 1,70E+07 | 1,70E+07 | recessive | AX-586150343 | GO:0016491 | Molecular Function:oxidoreductase activity | K11816 | indole-3-pyruvate monooxygenase [EC:1.14.13.168] | IPR000103 | Pyridine nucleotide-disulphide oxidoreductase, class-II |
|  |  | Pd08 | 1,70E+07 | 1,70E+07 | recessive | AX-586150343 | GO:0016491 | Molecular Function:oxidoreductase activity | K11816 | indole-3-pyruvate monooxygenase [EC:1.14.13.168] | IPR036188 | FAD/NAD(P)-binding domain superfamily |
|  |  | Pd08 | 1,70E+07 | 1,70E+07 | recessive | AX-586150343 | GO:0016491 | Molecular Function:oxidoreductase activity | K11816 | indole-3-pyruvate monooxygenase [EC:1.14.13.168] | IPR020946 | Flavin monooxygenase-like |
|  |  | Pd08 | 1,70E+07 | 1,70E+07 | recessive | AX-586150343 | GO:0050661 | Molecular Function:NADP binding | K11816 | indole-3-pyruvate monooxygenase [EC:1.14.13.168] | IPR000103 | Pyridine nucleotide-disulphide oxidoreductase, class-II |
|  |  | Pd08 | 1,70E+07 | 1,70E+07 | recessive | AX-586150343 | GO:0050661 | Molecular Function:NADP binding | K11816 | indole-3-pyruvate monooxygenase [EC:1.14.13.168] | IPR036188 | FAD/NAD(P)-binding domain superfamily |
|  |  | Pd08 | 1,70E+07 | 1,70E+07 | recessive | AX-586150343 | GO:0050661 | Molecular Function:NADP binding | K11816 | indole-3-pyruvate monooxygenase [EC:1.14.13.168] | IPR020946 | Flavin monooxygenase-like |
|  |  | Pd08 | 1,70E+07 | 1,70E+07 | recessive | AX-586150343 | GO:0055114 | Biological Process:oxidation-reduction process | K11816 | indole-3-pyruvate monooxygenase [EC:1.14.13.168] | IPR000103 | Pyridine nucleotide-disulphide oxidoreductase, class-II |
|  |  | Pd08 | 1,70E+07 | 1,70E+07 | recessive | AX-586150343 | GO:0055114 | Biological Process:oxidation-reduction process | K11816 | indole-3-pyruvate monooxygenase [EC:1.14.13.168] | IPR036188 | FAD/NAD(P)-binding domain superfamily |
|  |  | Pd08 | 1,70E+07 | 1,70E+07 | recessive | AX-586150343 | GO:0055114 | Biological Process:oxidation-reduction process | K11816 | indole-3-pyruvate monooxygenase [EC:1.14.13.168] | IPR020946 | Flavin monooxygenase-like |
|  |  | Pd08 | 1,70E+07 | 1,70E+07 | recessive | AX-586150343 | GO:0050660 | Molecular Function:flavin adenine dinucleotide binding | K11816 | indole-3-pyruvate monooxygenase [EC:1.14.13.168] | IPR000103 | Pyridine nucleotide-disulphide oxidoreductase, class-II |
|  |  | Pd08 | 1,70E+07 | 1,70E+07 | recessive | AX-586150343 | GO:0050660 | Molecular Function:flavin adenine dinucleotide binding | K11816 | indole-3-pyruvate monooxygenase [EC:1.14.13.168] | IPR036188 | FAD/NAD(P)-binding domain superfamily |
|  |  | Pd08 | 1,70E+07 | 1,70E+07 | recessive | AX-586150343 | GO:0050660 | Molecular Function:flavin adenine dinucleotide binding | K11816 | indole-3-pyruvate monooxygenase [EC:1.14.13.168] | IPR020946 | Flavin monooxygenase-like |
|  |  | Pd08 | 1,70E+07 | 1,70E+07 | recessive | AX-586150343 | GO:0004499 | Molecular Function:N,N-dimethylaniline monooxygenase activity | K11816 | indole-3-pyruvate monooxygenase [EC:1.14.13.168] | IPR000103 | Pyridine nucleotide-disulphide oxidoreductase, class-II |
|  |  | Pd08 | 1,70E+07 | 1,70E+07 | recessive | AX-586150343 | GO:0004499 | Molecular Function:N,N-dimethylaniline monooxygenase activity | K11816 | indole-3-pyruvate monooxygenase [EC:1.14.13.168] | IPR036188 | FAD/NAD(P)-binding domain superfamily |
|  |  | Pd08 | 1,70E+07 | 1,70E+07 | recessive | AX-586150343 | GO:0004499 | Molecular Function:N,N-dimethylaniline monooxygenase activity | K11816 | indole-3-pyruvate monooxygenase [EC:1.14.13.168] | IPR020946 | Flavin monooxygenase-like |
| **Prudul26A001570T1** | Splice region variant | Pd08 | 1,70E+07 | 1,70E+07 | recessive | AX-586150343 | GO:0005515 | Molecular Function:protein binding | K10357 | myosin V | IPR001609 | Myosin head, motor domain |
|  |  | Pd08 | 1,70E+07 | 1,70E+07 | recessive | AX-586150343 | GO:0005515 | Molecular Function:protein binding | K10357 | myosin V | IPR036961 | Kinesin motor domain superfamily |
|  |  | Pd08 | 1,70E+07 | 1,70E+07 | recessive | AX-586150343 | GO:0005515 | Molecular Function:protein binding | K10357 | myosin V | IPR027417 | P-loop containing nucleoside triphosphate hydrolase |
|  |  | Pd08 | 1,70E+07 | 1,70E+07 | recessive | AX-586150343 | GO:0005515 | Molecular Function:protein binding | K10357 | myosin V | IPR004009 | Myosin, N-terminal, SH3-like |
|  |  | Pd08 | 1,70E+07 | 1,70E+07 | recessive | AX-586150343 | GO:0005515 | Molecular Function:protein binding | K10357 | myosin V | IPR002710 | Dilute domain |
|  |  | Pd08 | 1,70E+07 | 1,70E+07 | recessive | AX-586150343 | GO:0005515 | Molecular Function:protein binding | K10357 | myosin V | IPR000048 | IQ motif, EF-hand binding site |
|  |  | Pd08 | 1,70E+07 | 1,70E+07 | recessive | AX-586150343 | GO:0016459 | Cellular Component:myosin complex | K10357 | myosin V | IPR001609 | Myosin head, motor domain |
|  |  | Pd08 | 1,70E+07 | 1,70E+07 | recessive | AX-586150343 | GO:0016459 | Cellular Component:myosin complex | K10357 | myosin V | IPR036961 | Kinesin motor domain superfamily |
|  |  | Pd08 | 1,70E+07 | 1,70E+07 | recessive | AX-586150343 | GO:0016459 | Cellular Component:myosin complex | K10357 | myosin V | IPR027417 | P-loop containing nucleoside triphosphate hydrolase |
|  |  | Pd08 | 1,70E+07 | 1,70E+07 | recessive | AX-586150343 | GO:0016459 | Cellular Component:myosin complex | K10357 | myosin V | IPR004009 | Myosin, N-terminal, SH3-like |
|  |  | Pd08 | 1,70E+07 | 1,70E+07 | recessive | AX-586150343 | GO:0016459 | Cellular Component:myosin complex | K10357 | myosin V | IPR002710 | Dilute domain |
|  |  | Pd08 | 1,70E+07 | 1,70E+07 | recessive | AX-586150343 | GO:0016459 | Cellular Component:myosin complex | K10357 | myosin V | IPR000048 | IQ motif, EF-hand binding site |
|  |  | Pd08 | 1,70E+07 | 1,70E+07 | recessive | AX-586150343 | GO:0005524 | Molecular Function:ATP binding | K10357 | myosin V | IPR001609 | Myosin head, motor domain |
|  |  | Pd08 | 1,70E+07 | 1,70E+07 | recessive | AX-586150343 | GO:0005524 | Molecular Function:ATP binding | K10357 | myosin V | IPR036961 | Kinesin motor domain superfamily |
|  |  | Pd08 | 1,70E+07 | 1,70E+07 | recessive | AX-586150343 | GO:0005524 | Molecular Function:ATP binding | K10357 | myosin V | IPR027417 | P-loop containing nucleoside triphosphate hydrolase |
|  |  | Pd08 | 1,70E+07 | 1,70E+07 | recessive | AX-586150343 | GO:0005524 | Molecular Function:ATP binding | K10357 | myosin V | IPR004009 | Myosin, N-terminal, SH3-like |
|  |  | Pd08 | 1,70E+07 | 1,70E+07 | recessive | AX-586150343 | GO:0005524 | Molecular Function:ATP binding | K10357 | myosin V | IPR002710 | Dilute domain |
|  |  | Pd08 | 1,70E+07 | 1,70E+07 | recessive | AX-586150343 | GO:0005524 | Molecular Function:ATP binding | K10357 | myosin V | IPR000048 | IQ motif, EF-hand binding site |
|  |  | Pd08 | 1,70E+07 | 1,70E+07 | recessive | AX-586150343 | GO:0003774 | Molecular Function:motor activity | K10357 | myosin V | IPR001609 | Myosin head, motor domain |
|  |  | Pd08 | 1,70E+07 | 1,70E+07 | recessive | AX-586150343 | GO:0003774 | Molecular Function:motor activity | K10357 | myosin V | IPR036961 | Kinesin motor domain superfamily |
|  |  | Pd08 | 1,70E+07 | 1,70E+07 | recessive | AX-586150343 | GO:0003774 | Molecular Function:motor activity | K10357 | myosin V | IPR027417 | P-loop containing nucleoside triphosphate hydrolase |
|  |  | Pd08 | 1,70E+07 | 1,70E+07 | recessive | AX-586150343 | GO:0003774 | Molecular Function:motor activity | K10357 | myosin V | IPR004009 | Myosin, N-terminal, SH3-like |
|  |  | Pd08 | 1,70E+07 | 1,70E+07 | recessive | AX-586150343 | GO:0003774 | Molecular Function:motor activity | K10357 | myosin V | IPR002710 | Dilute domain |
|  |  | Pd08 | 1,70E+07 | 1,70E+07 | recessive | AX-586150343 | GO:0003774 | Molecular Function:motor activity | K10357 | myosin V | IPR000048 | IQ motif, EF-hand binding site |
| **Prudul26A025459T1** |  | Pd08 | 1,70E+07 | 1,70E+07 | recessive | AX-586150343 | GO:0030150 | Biological Process:protein import into mitochondrial matrix | K17796 | mitochondrial import inner membrane translocase subunit TIM21 | IPR038552 | Tim21 IMS domain superfamily |
|  |  | Pd08 | 1,70E+07 | 1,70E+07 | recessive | AX-586150343 | GO:0030150 | Biological Process:protein import into mitochondrial matrix | K17796 | mitochondrial import inner membrane translocase subunit TIM21 | IPR013261 | Mitochondrial import inner membrane translocase subunit Tim21 |
|  |  | Pd08 | 1,70E+07 | 1,70E+07 | recessive | AX-586150343 | GO:0005744 | Cellular Component:TIM23 mitochondrial import inner membrane translocase complex | K17796 | mitochondrial import inner membrane translocase subunit TIM21 | IPR038552 | Tim21 IMS domain superfamily |
|  |  | Pd08 | 1,70E+07 | 1,70E+07 | recessive | AX-586150343 | GO:0005744 | Cellular Component:TIM23 mitochondrial import inner membrane translocase complex | K17796 | mitochondrial import inner membrane translocase subunit TIM21 | IPR013261 | Mitochondrial import inner membrane translocase subunit Tim21 |
| **Prudul26A025459T2** |  | Pd08 | 1,70E+07 | 1,70E+07 | recessive | AX-586150343 | GO:0005744 | Cellular Component:TIM23 mitochondrial import inner membrane translocase complex | K17796 | mitochondrial import inner membrane translocase subunit TIM21 | IPR038552 | Tim21 IMS domain superfamily |
|  |  | Pd08 | 1,70E+07 | 1,70E+07 | recessive | AX-586150343 | GO:0005744 | Cellular Component:TIM23 mitochondrial import inner membrane translocase complex | K17796 | mitochondrial import inner membrane translocase subunit TIM21 | IPR013261 | Mitochondrial import inner membrane translocase subunit Tim21 |
|  |  | Pd08 | 1,70E+07 | 1,70E+07 | recessive | AX-586150343 | GO:0030150 | Biological Process:protein import into mitochondrial matrix | K17796 | mitochondrial import inner membrane translocase subunit TIM21 | IPR038552 | Tim21 IMS domain superfamily |
|  |  | Pd08 | 1,70E+07 | 1,70E+07 | recessive | AX-586150343 | GO:0030150 | Biological Process:protein import into mitochondrial matrix | K17796 | mitochondrial import inner membrane translocase subunit TIM21 | IPR013261 | Mitochondrial import inner membrane translocase subunit Tim21 |
| **Prudul26A015859T1** |  | Pd08 | 1,70E+07 | 1,70E+07 | recessive | AX-586150343 | NA | NA | K15078 | structure-specific endonuclease subunit SLX1 [EC:3.6.1.-] | IPR027417 | P-loop containing nucleoside triphosphate hydrolase |
| **Prudul26A006689T1** |  | Pd08 | 1,70E+07 | 1,70E+07 | recessive | AX-586150343 | NA | NA | K15078 | structure-specific endonuclease subunit SLX1 [EC:3.6.1.-] | IPR032675 | Leucine-rich repeat domain superfamily |
| **Prudul26A002617T1** |  | Pd08 | 1,70E+07 | 1,70E+07 | recessive | AX-586150343 | NA | NA | K11982 | E3 ubiquitin-protein ligase RNF115/126 [EC:2.3.2.27] | IPR013083 | Zinc finger, RING/FYVE/PHD-type |
|  |  | Pd08 | 1,70E+07 | 1,70E+07 | recessive | AX-586150343 | NA | NA | K11982 | E3 ubiquitin-protein ligase RNF115/126 [EC:2.3.2.27] | IPR001841 | Zinc finger, RING-type |
| **Prudul26A011855T1** |  | Pd08 | 1,70E+07 | 1,70E+07 | recessive | AX-586150343 | NA | NA | NA | NA | IPR035892 | C2 domain superfamily |
|  |  | Pd08 | 1,70E+07 | 1,70E+07 | recessive | AX-586150343 | NA | NA | NA | NA | IPR013583 | Phosphoribosyltransferase C-terminal |
|  |  | Pd08 | 1,70E+07 | 1,70E+07 | recessive | AX-586150343 | NA | NA | NA | NA | IPR000008 | C2 domain |
| **Prudul26A021313T1** |  | Pd08 | 1,70E+07 | 1,70E+07 | recessive | AX-586150343 | GO:0005515 | Molecular Function:protein binding | NA | NA | IPR017451 | F-box associated interaction domain |
|  |  | Pd08 | 1,70E+07 | 1,70E+07 | recessive | AX-586150343 | GO:0005515 | Molecular Function:protein binding | NA | NA | IPR036047 | F-box-like domain superfamily |
|  |  | Pd08 | 1,70E+07 | 1,70E+07 | recessive | AX-586150343 | GO:0005515 | Molecular Function:protein binding | NA | NA | IPR001810 | F-box domain |
| **Prudul26A030627T1** |  | Pd08 | 1,70E+07 | 1,70E+07 | recessive | AX-586150343 | GO:0005515 | Molecular Function:protein binding | NA | NA | IPR036047 | F-box-like domain superfamily |
|  |  | Pd08 | 1,70E+07 | 1,70E+07 | recessive | AX-586150343 | GO:0005515 | Molecular Function:protein binding | NA | NA | IPR001810 | F-box domain |
|  |  | Pd08 | 1,70E+07 | 1,70E+07 | recessive | AX-586150343 | GO:0005515 | Molecular Function:protein binding | NA | NA | IPR032675 | Leucine-rich repeat domain superfamily |
| **Prudul26A030093T1** |  | Pd08 | 1,70E+07 | 1,70E+07 | recessive | AX-586150343 | GO:0003677 | Molecular Function:DNA binding | K11292 | transcription elongation factor SPT6 | IPR028231 | Transcription elongation factor Spt6, YqgF domain |
|  |  | Pd08 | 1,70E+07 | 1,70E+07 | recessive | AX-586150343 | GO:0003677 | Molecular Function:DNA binding | K11292 | transcription elongation factor SPT6 | IPR036860 | SH2 domain superfamily |
|  |  | Pd08 | 1,70E+07 | 1,70E+07 | recessive | AX-586150343 | GO:0003677 | Molecular Function:DNA binding | K11292 | transcription elongation factor SPT6 | IPR023319 | Tex-like protein, HTH domain superfamily |
|  |  | Pd08 | 1,70E+07 | 1,70E+07 | recessive | AX-586150343 | GO:0003677 | Molecular Function:DNA binding | K11292 | transcription elongation factor SPT6 | IPR012340 | Nucleic acid-binding, OB-fold |
|  |  | Pd08 | 1,70E+07 | 1,70E+07 | recessive | AX-586150343 | GO:0003677 | Molecular Function:DNA binding | K11292 | transcription elongation factor SPT6 | IPR023323 | Tex-like domain superfamily |
|  |  | Pd08 | 1,70E+07 | 1,70E+07 | recessive | AX-586150343 | GO:0003677 | Molecular Function:DNA binding | K11292 | transcription elongation factor SPT6 | IPR027999 | Death-like domain of Spt6 |
|  |  | Pd08 | 1,70E+07 | 1,70E+07 | recessive | AX-586150343 | GO:0003677 | Molecular Function:DNA binding | K11292 | transcription elongation factor SPT6 | IPR017072 | Transcription elongation factor Spt6 |
|  |  | Pd08 | 1,70E+07 | 1,70E+07 | recessive | AX-586150343 | GO:0003677 | Molecular Function:DNA binding | K11292 | transcription elongation factor SPT6 | IPR028088 | Helix-turn-helix DNA-binding domain of Spt6 |
|  |  | Pd08 | 1,70E+07 | 1,70E+07 | recessive | AX-586150343 | GO:0003677 | Molecular Function:DNA binding | K11292 | transcription elongation factor SPT6 | IPR032706 | Transcription elongation factor Spt6, helix-hairpin-helix motif |
|  |  | Pd08 | 1,70E+07 | 1,70E+07 | recessive | AX-586150343 | GO:0003677 | Molecular Function:DNA binding | K11292 | transcription elongation factor SPT6 | IPR035420 | Spt6, SH2 domain |
|  |  | Pd08 | 1,70E+07 | 1,70E+07 | recessive | AX-586150343 | GO:0003677 | Molecular Function:DNA binding | K11292 | transcription elongation factor SPT6 | IPR028083 | Spt6 acidic, N-terminal domain |
|  |  | Pd08 | 1,70E+07 | 1,70E+07 | recessive | AX-586150343 | GO:0003677 | Molecular Function:DNA binding | K11292 | transcription elongation factor SPT6 | IPR003029 | S1 domain |
|  |  | Pd08 | 1,70E+07 | 1,70E+07 | recessive | AX-586150343 | GO:0003677 | Molecular Function:DNA binding | K11292 | transcription elongation factor SPT6 | IPR010994 | RuvA domain 2-like |
|  |  | Pd08 | 1,70E+07 | 1,70E+07 | recessive | AX-586150343 | GO:0003677 | Molecular Function:DNA binding | K11292 | transcription elongation factor SPT6 | IPR012337 | Ribonuclease H-like superfamily |
|  |  | Pd08 | 1,70E+07 | 1,70E+07 | recessive | AX-586150343 | GO:0003677 | Molecular Function:DNA binding | K11292 | transcription elongation factor SPT6 | IPR037027 | YqgF/RNase H-like domain superfamily |
|  |  | Pd08 | 1,70E+07 | 1,70E+07 | recessive | AX-586150343 | GO:0003676 | Molecular Function:nucleic acid binding | K11292 | transcription elongation factor SPT6 | IPR028231 | Transcription elongation factor Spt6, YqgF domain |
|  |  | Pd08 | 1,70E+07 | 1,70E+07 | recessive | AX-586150343 | GO:0003676 | Molecular Function:nucleic acid binding | K11292 | transcription elongation factor SPT6 | IPR036860 | SH2 domain superfamily |
|  |  | Pd08 | 1,70E+07 | 1,70E+07 | recessive | AX-586150343 | GO:0003676 | Molecular Function:nucleic acid binding | K11292 | transcription elongation factor SPT6 | IPR023319 | Tex-like protein, HTH domain superfamily |
|  |  | Pd08 | 1,70E+07 | 1,70E+07 | recessive | AX-586150343 | GO:0003676 | Molecular Function:nucleic acid binding | K11292 | transcription elongation factor SPT6 | IPR012340 | Nucleic acid-binding, OB-fold |
|  |  | Pd08 | 1,70E+07 | 1,70E+07 | recessive | AX-586150343 | GO:0003676 | Molecular Function:nucleic acid binding | K11292 | transcription elongation factor SPT6 | IPR023323 | Tex-like domain superfamily |
|  |  | Pd08 | 1,70E+07 | 1,70E+07 | recessive | AX-586150343 | GO:0003676 | Molecular Function:nucleic acid binding | K11292 | transcription elongation factor SPT6 | IPR027999 | Death-like domain of Spt6 |
|  |  | Pd08 | 1,70E+07 | 1,70E+07 | recessive | AX-586150343 | GO:0003676 | Molecular Function:nucleic acid binding | K11292 | transcription elongation factor SPT6 | IPR017072 | Transcription elongation factor Spt6 |
|  |  | Pd08 | 1,70E+07 | 1,70E+07 | recessive | AX-586150343 | GO:0003676 | Molecular Function:nucleic acid binding | K11292 | transcription elongation factor SPT6 | IPR028088 | Helix-turn-helix DNA-binding domain of Spt6 |
|  |  | Pd08 | 1,70E+07 | 1,70E+07 | recessive | AX-586150343 | GO:0003676 | Molecular Function:nucleic acid binding | K11292 | transcription elongation factor SPT6 | IPR032706 | Transcription elongation factor Spt6, helix-hairpin-helix motif |
|  |  | Pd08 | 1,70E+07 | 1,70E+07 | recessive | AX-586150343 | GO:0003676 | Molecular Function:nucleic acid binding | K11292 | transcription elongation factor SPT6 | IPR035420 | Spt6, SH2 domain |
|  |  | Pd08 | 1,70E+07 | 1,70E+07 | recessive | AX-586150343 | GO:0003676 | Molecular Function:nucleic acid binding | K11292 | transcription elongation factor SPT6 | IPR028083 | Spt6 acidic, N-terminal domain |
|  |  | Pd08 | 1,70E+07 | 1,70E+07 | recessive | AX-586150343 | GO:0003676 | Molecular Function:nucleic acid binding | K11292 | transcription elongation factor SPT6 | IPR003029 | S1 domain |
|  |  | Pd08 | 1,70E+07 | 1,70E+07 | recessive | AX-586150343 | GO:0003676 | Molecular Function:nucleic acid binding | K11292 | transcription elongation factor SPT6 | IPR010994 | RuvA domain 2-like |
|  |  | Pd08 | 1,70E+07 | 1,70E+07 | recessive | AX-586150343 | GO:0003676 | Molecular Function:nucleic acid binding | K11292 | transcription elongation factor SPT6 | IPR012337 | Ribonuclease H-like superfamily |
|  |  | Pd08 | 1,70E+07 | 1,70E+07 | recessive | AX-586150343 | GO:0003676 | Molecular Function:nucleic acid binding | K11292 | transcription elongation factor SPT6 | IPR037027 | YqgF/RNase H-like domain superfamily |
|  |  | Pd08 | 1,70E+07 | 1,70E+07 | recessive | AX-586150343 | GO:0006139 | Biological Process:nucleobase-containing compound metabolic process | K11292 | transcription elongation factor SPT6 | IPR028231 | Transcription elongation factor Spt6, YqgF domain |
|  |  | Pd08 | 1,70E+07 | 1,70E+07 | recessive | AX-586150343 | GO:0006139 | Biological Process:nucleobase-containing compound metabolic process | K11292 | transcription elongation factor SPT6 | IPR036860 | SH2 domain superfamily |
|  |  | Pd08 | 1,70E+07 | 1,70E+07 | recessive | AX-586150343 | GO:0006139 | Biological Process:nucleobase-containing compound metabolic process | K11292 | transcription elongation factor SPT6 | IPR023319 | Tex-like protein, HTH domain superfamily |
|  |  | Pd08 | 1,70E+07 | 1,70E+07 | recessive | AX-586150343 | GO:0006139 | Biological Process:nucleobase-containing compound metabolic process | K11292 | transcription elongation factor SPT6 | IPR012340 | Nucleic acid-binding, OB-fold |
|  |  | Pd08 | 1,70E+07 | 1,70E+07 | recessive | AX-586150343 | GO:0006139 | Biological Process:nucleobase-containing compound metabolic process | K11292 | transcription elongation factor SPT6 | IPR023323 | Tex-like domain superfamily |
|  |  | Pd08 | 1,70E+07 | 1,70E+07 | recessive | AX-586150343 | GO:0006139 | Biological Process:nucleobase-containing compound metabolic process | K11292 | transcription elongation factor SPT6 | IPR027999 | Death-like domain of Spt6 |
|  |  | Pd08 | 1,70E+07 | 1,70E+07 | recessive | AX-586150343 | GO:0006139 | Biological Process:nucleobase-containing compound metabolic process | K11292 | transcription elongation factor SPT6 | IPR017072 | Transcription elongation factor Spt6 |
|  |  | Pd08 | 1,70E+07 | 1,70E+07 | recessive | AX-586150343 | GO:0006139 | Biological Process:nucleobase-containing compound metabolic process | K11292 | transcription elongation factor SPT6 | IPR028088 | Helix-turn-helix DNA-binding domain of Spt6 |
|  |  | Pd08 | 1,70E+07 | 1,70E+07 | recessive | AX-586150343 | GO:0006139 | Biological Process:nucleobase-containing compound metabolic process | K11292 | transcription elongation factor SPT6 | IPR032706 | Transcription elongation factor Spt6, helix-hairpin-helix motif |
|  |  | Pd08 | 1,70E+07 | 1,70E+07 | recessive | AX-586150343 | GO:0006139 | Biological Process:nucleobase-containing compound metabolic process | K11292 | transcription elongation factor SPT6 | IPR035420 | Spt6, SH2 domain |
|  |  | Pd08 | 1,70E+07 | 1,70E+07 | recessive | AX-586150343 | GO:0006139 | Biological Process:nucleobase-containing compound metabolic process | K11292 | transcription elongation factor SPT6 | IPR028083 | Spt6 acidic, N-terminal domain |
|  |  | Pd08 | 1,70E+07 | 1,70E+07 | recessive | AX-586150343 | GO:0006139 | Biological Process:nucleobase-containing compound metabolic process | K11292 | transcription elongation factor SPT6 | IPR003029 | S1 domain |
|  |  | Pd08 | 1,70E+07 | 1,70E+07 | recessive | AX-586150343 | GO:0006139 | Biological Process:nucleobase-containing compound metabolic process | K11292 | transcription elongation factor SPT6 | IPR010994 | RuvA domain 2-like |
|  |  | Pd08 | 1,70E+07 | 1,70E+07 | recessive | AX-586150343 | GO:0006139 | Biological Process:nucleobase-containing compound metabolic process | K11292 | transcription elongation factor SPT6 | IPR012337 | Ribonuclease H-like superfamily |
|  |  | Pd08 | 1,70E+07 | 1,70E+07 | recessive | AX-586150343 | GO:0006139 | Biological Process:nucleobase-containing compound metabolic process | K11292 | transcription elongation factor SPT6 | IPR037027 | YqgF/RNase H-like domain superfamily |
|  |  | Pd08 | 1,70E+07 | 1,70E+07 | recessive | AX-586150343 | GO:0032968 | Biological Process:positive regulation of transcription elongation from RNA polymerase II promoter | K11292 | transcription elongation factor SPT6 | IPR028231 | Transcription elongation factor Spt6, YqgF domain |
|  |  | Pd08 | 1,70E+07 | 1,70E+07 | recessive | AX-586150343 | GO:0032968 | Biological Process:positive regulation of transcription elongation from RNA polymerase II promoter | K11292 | transcription elongation factor SPT6 | IPR036860 | SH2 domain superfamily |
|  |  | Pd08 | 1,70E+07 | 1,70E+07 | recessive | AX-586150343 | GO:0032968 | Biological Process:positive regulation of transcription elongation from RNA polymerase II promoter | K11292 | transcription elongation factor SPT6 | IPR023319 | Tex-like protein, HTH domain superfamily |
|  |  | Pd08 | 1,70E+07 | 1,70E+07 | recessive | AX-586150343 | GO:0032968 | Biological Process:positive regulation of transcription elongation from RNA polymerase II promoter | K11292 | transcription elongation factor SPT6 | IPR012340 | Nucleic acid-binding, OB-fold |
|  |  | Pd08 | 1,70E+07 | 1,70E+07 | recessive | AX-586150343 | GO:0032968 | Biological Process:positive regulation of transcription elongation from RNA polymerase II promoter | K11292 | transcription elongation factor SPT6 | IPR023323 | Tex-like domain superfamily |
|  |  | Pd08 | 1,70E+07 | 1,70E+07 | recessive | AX-586150343 | GO:0032968 | Biological Process:positive regulation of transcription elongation from RNA polymerase II promoter | K11292 | transcription elongation factor SPT6 | IPR027999 | Death-like domain of Spt6 |
|  |  | Pd08 | 1,70E+07 | 1,70E+07 | recessive | AX-586150343 | GO:0032968 | Biological Process:positive regulation of transcription elongation from RNA polymerase II promoter | K11292 | transcription elongation factor SPT6 | IPR017072 | Transcription elongation factor Spt6 |
|  |  | Pd08 | 1,70E+07 | 1,70E+07 | recessive | AX-586150343 | GO:0032968 | Biological Process:positive regulation of transcription elongation from RNA polymerase II promoter | K11292 | transcription elongation factor SPT6 | IPR028088 | Helix-turn-helix DNA-binding domain of Spt6 |
|  |  | Pd08 | 1,70E+07 | 1,70E+07 | recessive | AX-586150343 | GO:0032968 | Biological Process:positive regulation of transcription elongation from RNA polymerase II promoter | K11292 | transcription elongation factor SPT6 | IPR032706 | Transcription elongation factor Spt6, helix-hairpin-helix motif |
|  |  | Pd08 | 1,70E+07 | 1,70E+07 | recessive | AX-586150343 | GO:0032968 | Biological Process:positive regulation of transcription elongation from RNA polymerase II promoter | K11292 | transcription elongation factor SPT6 | IPR035420 | Spt6, SH2 domain |
|  |  | Pd08 | 1,70E+07 | 1,70E+07 | recessive | AX-586150343 | GO:0032968 | Biological Process:positive regulation of transcription elongation from RNA polymerase II promoter | K11292 | transcription elongation factor SPT6 | IPR028083 | Spt6 acidic, N-terminal domain |
|  |  | Pd08 | 1,70E+07 | 1,70E+07 | recessive | AX-586150343 | GO:0032968 | Biological Process:positive regulation of transcription elongation from RNA polymerase II promoter | K11292 | transcription elongation factor SPT6 | IPR003029 | S1 domain |
|  |  | Pd08 | 1,70E+07 | 1,70E+07 | recessive | AX-586150343 | GO:0032968 | Biological Process:positive regulation of transcription elongation from RNA polymerase II promoter | K11292 | transcription elongation factor SPT6 | IPR010994 | RuvA domain 2-like |
|  |  | Pd08 | 1,70E+07 | 1,70E+07 | recessive | AX-586150343 | GO:0032968 | Biological Process:positive regulation of transcription elongation from RNA polymerase II promoter | K11292 | transcription elongation factor SPT6 | IPR012337 | Ribonuclease H-like superfamily |
|  |  | Pd08 | 1,70E+07 | 1,70E+07 | recessive | AX-586150343 | GO:0032968 | Biological Process:positive regulation of transcription elongation from RNA polymerase II promoter | K11292 | transcription elongation factor SPT6 | IPR037027 | YqgF/RNase H-like domain superfamily |
| **Prudul26A025233T1** |  | Pd08 | 1,70E+07 | 1,70E+07 | recessive | AX-586150343 | GO:0016702 | Molecular Function:oxidoreductase activity, acting on single donors with incorporation of molecular oxygen, incorporation of two atoms of oxygen | K10712 | cysteamine dioxygenase [EC:1.13.11.19] | IPR012864 | Cysteine oxygenase/2-aminoethanethiol dioxygenase |
|  |  | Pd08 | 1,70E+07 | 1,70E+07 | recessive | AX-586150343 | GO:0055114 | Biological Process:oxidation-reduction process | K10712 | cysteamine dioxygenase [EC:1.13.11.19] | IPR012864 | Cysteine oxygenase/2-aminoethanethiol dioxygenase |
| **Prudul26A025733T1** |  | Pd08 | 1,70E+07 | 1,70E+07 | recessive | AX-586150343 | GO:0055114 | Biological Process:oxidation-reduction process | K10712 | cysteamine dioxygenase [EC:1.13.11.19] | IPR011051 | RmlC-like cupin domain superfamily |
|  |  | Pd08 | 1,70E+07 | 1,70E+07 | recessive | AX-586150343 | GO:0055114 | Biological Process:oxidation-reduction process | K10712 | cysteamine dioxygenase [EC:1.13.11.19] | IPR012864 | Cysteine oxygenase/2-aminoethanethiol dioxygenase |
|  |  | Pd08 | 1,70E+07 | 1,70E+07 | recessive | AX-586150343 | GO:0016702 | Molecular Function:oxidoreductase activity, acting on single donors with incorporation of molecular oxygen, incorporation of two atoms of oxygen | K10712 | cysteamine dioxygenase [EC:1.13.11.19] | IPR011051 | RmlC-like cupin domain superfamily |
|  |  | Pd08 | 1,70E+07 | 1,70E+07 | recessive | AX-586150343 | GO:0016702 | Molecular Function:oxidoreductase activity, acting on single donors with incorporation of molecular oxygen, incorporation of two atoms of oxygen | K10712 | cysteamine dioxygenase [EC:1.13.11.19] | IPR012864 | Cysteine oxygenase/2-aminoethanethiol dioxygenase |
| **Prudul26A018376T1** |  | Pd08 | 1,70E+07 | 1,70E+07 | recessive | AX-586150343 | NA | NA | K03136 | transcription initiation factor TFIIE subunit alpha | IPR036390 | Winged helix DNA-binding domain superfamily |
|  |  | Pd08 | 1,70E+07 | 1,70E+07 | recessive | AX-586150343 | NA | NA | K03136 | transcription initiation factor TFIIE subunit alpha | IPR039997 | Transcription factor E |
| **Prudul26A011107T1** |  | Pd08 | 1,70E+07 | 1,70E+07 | recessive | AX-586150343 | GO:0016773 | Molecular Function:phosphotransferase activity, alcohol group as acceptor | NA | NA | IPR002173 | Carbohydrate/puine kinase, PfkB, conserved site |
|  |  | Pd08 | 1,70E+07 | 1,70E+07 | recessive | AX-586150343 | GO:0016773 | Molecular Function:phosphotransferase activity, alcohol group as acceptor | NA | NA | IPR002139 | Ribokinase/fructokinase |
|  |  | Pd08 | 1,70E+07 | 1,70E+07 | recessive | AX-586150343 | GO:0016773 | Molecular Function:phosphotransferase activity, alcohol group as acceptor | NA | NA | IPR029056 | Ribokinase-like |
|  |  | Pd08 | 1,70E+07 | 1,70E+07 | recessive | AX-586150343 | GO:0016773 | Molecular Function:phosphotransferase activity, alcohol group as acceptor | NA | NA | IPR011611 | Carbohydrate kinase PfkB |
|  |  | Pd08 | 1,70E+07 | 1,70E+07 | recessive | AX-586150343 | GO:0016301 | Molecular Function:kinase activity | NA | NA | IPR002173 | Carbohydrate/puine kinase, PfkB, conserved site |
|  |  | Pd08 | 1,70E+07 | 1,70E+07 | recessive | AX-586150343 | GO:0016301 | Molecular Function:kinase activity | NA | NA | IPR002139 | Ribokinase/fructokinase |
|  |  | Pd08 | 1,70E+07 | 1,70E+07 | recessive | AX-586150343 | GO:0016301 | Molecular Function:kinase activity | NA | NA | IPR029056 | Ribokinase-like |
|  |  | Pd08 | 1,70E+07 | 1,70E+07 | recessive | AX-586150343 | GO:0016301 | Molecular Function:kinase activity | NA | NA | IPR011611 | Carbohydrate kinase PfkB |
